# Soft Matrix Promotes Cardiac Reprogramming via Inhibition of YAP/TAZ and Suppression of Fibroblast Signatures

**DOI:** 10.1016/j.stemcr.2020.07.022

**Published:** 2020-08-27

**Authors:** Shota Kurotsu, Taketaro Sadahiro, Ryo Fujita, Hidenori Tani, Hiroyuki Yamakawa, Fumiya Tamura, Mari Isomi, Hidenori Kojima, Yu Yamada, Yuto Abe, Yoshiko Murakata, Tatsuya Akiyama, Naoto Muraoka, Ichiro Harada, Takeshi Suzuki, Keiichi Fukuda, Masaki Ieda

**Affiliations:** 1 Department of Cardiology, Keio University School of Medicine, 35 Shinanomachi, Shinjuku-ku, Tokyo 160-8582, Japan; 2 Department of Cardiology, Faculty of Medicine, University of Tsukuba, 1-1-1 Tennoudai, Tsukuba City, Ibaraki 305-8575, Japan; 3 Division of Regenerative Medicine, Transborder Medical Research Center, University of Tsukuba, 1-1-1 Tennoudai, Tsukuba City, Ibaraki 305-8575, Japan; 4 Department of Respiratory Medicine, Faculty of Medicine, University of Tsukuba, 1-1-1 Tennoudai, Tsukuba City, Ibaraki 305-8575, Japan; 5 Medical Products Technology Development Center, R&D Headquarters, Canon Inc., 3-30-2 Shimomaruko, Ohta-ku, Tokyo 146-8501, Japan; 6 Division of Basic Biological Sciences, Faculty of Pharmacy, Keio University, 1-5-30 Shibakoen, Minato-ku, Tokyo 105-8512, Japan

**Keywords:** soft matrix, YAP/TAZ, cardiac reprogramming, mechanotransduction, myocardium

## Abstract

Direct cardiac reprogramming holds great potential for regenerative medicine. However, it remains inefficient, and induced cardiomyocytes (iCMs) generated *in vitro* are less mature than those *in vivo*, suggesting that undefined extrinsic factors may regulate cardiac reprogramming. Previous *in vitro* studies mainly used hard polystyrene dishes, yet the effect of substrate rigidity on cardiac reprogramming remains unclear. Thus, we developed a Matrigel-based hydrogel culture system to determine the roles of matrix stiffness and mechanotransduction in cardiac reprogramming. We found that soft matrix comparable with native myocardium promoted the efficiency and quality of cardiac reprogramming. Mechanistically, soft matrix enhanced cardiac reprogramming via inhibition of integrin, Rho/ROCK, actomyosin, and YAP/TAZ signaling and suppression of fibroblast programs, which were activated on rigid substrates. Soft substrate further enhanced cardiac reprogramming with Sendai virus vectors via YAP/TAZ suppression, increasing the reprogramming efficiency up to ∼15%. Thus, mechanotransduction could provide new targets for improving cardiac reprogramming.

## Highlights

• Hydrogel culture reveals the role of mechanotransduction in cardiac reprogramming
• Soft ECM comparable with native myocardium promotes cardiac reprogramming
• Soft ECM promotes cardiac reprogramming via YAP/TAZ/fibroblast signaling inhibition
• Soft ECM promotes Sendai virus vector-mediated cardiac reprogramming

## Graphical Abstract

**Figure:**
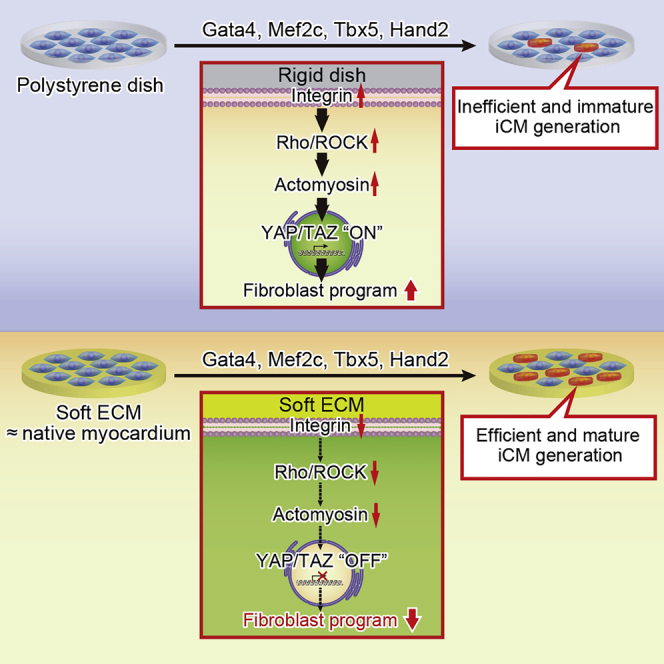

## Teaser

In this article, Ieda and colleagues showed that a soft matrix, which is comparable with native myocardium, efficiently promoted cardiac reprogramming. This soft matrix enhanced cardiac reprogramming via inhibition of integrin, Rho/ROCK, actomyosin, and YAP/TAZ signaling and subsequent suppression of fibroblast programs, which were activated on conventional rigid substrates, thus demonstrating that mechanotransduction plays a critical role in cardiac reprogramming.

## Introduction

Direct reprogramming generates the desired cell types from fibroblasts without passing through a stem cell state by overexpressing tissue-specific transcription factors (Sadahiro et al., 2015). This new technology has wide applications in disease modeling, drug discovery, and regenerative medicine (Isomi et al., 2019; Sadahiro et al., 2018; Srivastava and DeWitt, 2016). We and others reported the generation of induced cardiomyocytes (iCMs) from fibroblasts using cardiac transcription factors, including GATA4, MEF2C, and TBX5 (GMT) or GMT plus HAND2 (GHMT) (Ieda et al., 2010; Song et al., 2012). *In vivo* cardiac reprogramming by gene transfer of GMT or GHMT into mouse hearts reprogrammed resident cardiac fibroblasts (CFs) into iCMs, improved cardiac function, and reduced fibrosis after myocardial infarction (MI) (Inagawa et al., 2012; Miyamoto et al., 2018; Qian et al., 2012; Song et al., 2012). However, the cardiac reprogramming process remains inefficient and iCMs generated *in vitro* are immature, suggesting that undefined extrinsic factors may regulate the efficiency and quality of cardiac reprogramming (Qian et al., 2012; Song et al., 2012; Srivastava and DeWitt, 2016; Yamakawa et al., 2015; Zhao et al., 2015). Previous cardiac reprogramming studies have mainly used conventional rigid polystyrene dishes (PS; ∼GPa) that exhibit distinct physical properties from native myocardium (10–20 kPa) (Berry et al., 2006; Engler et al., 2006); thus, the effects of substrate stiffness and mechanotransduction on cardiac reprogramming remain elusive.

Cells sense underlying matrix rigidity and propagate biochemical and biophysical signals along the cytoskeleton to the nucleus, altering gene expression and cellular function (Crowder et al., 2016; Dupont, 2016; Lyon et al., 2015). Previous studies demonstrated that stiffness of the extracellular matrix (ECM) changes cell adhesion, proliferation, and differentiation (Crowder et al., 2016; Engler et al., 2006, 2008; Lyon et al., 2015; Yahalom-Ronen et al., 2015). Engler et al., 2006 demonstrated that mesenchymal stem cells (MSCs) cultured on polyacrylamide gels differentiated into specific cell types that corresponded to the stiffness of their incorporated tissues; soft matrices specified the MSCs to the adipogenic lineage, while stiff matrices differentiated them toward the osteogenic lineage. Further mechanistic studies revealed that two highly related transcriptional coactivators, Yes-associated protein (YAP) and transcriptional coactivator with PDZ-binding domain (TAZ), were responsible for sensing ECM rigidity and acted as key regulators for mechanotransduction (Dupont, 2016; Dupont et al., 2011). It has been reported that YAP activation promotes induced pluripotent stem cell (iPSC) reprogramming (Lian et al., 2010); however, the roles of YAP/TAZ in direct cardiac reprogramming remain unknown. Given that the myocardium becomes stiffer than healthy myocardium after MI via accumulation of ECM and fibrosis, understanding the effects of matrix stiffness and mechanotransduction in cardiac reprogramming would be clinically relevant (Berry et al., 2006; Engler et al., 2008). Therefore, in this study, we developed a Matrigel-based hydrogel culture system to investigate the mechanobiology in cardiac reprogramming and the signaling pathway involved.

## Results

### Soft ECM Comparable with the Myocardium Promotes Cardiac Reprogramming

**Figure 1:**
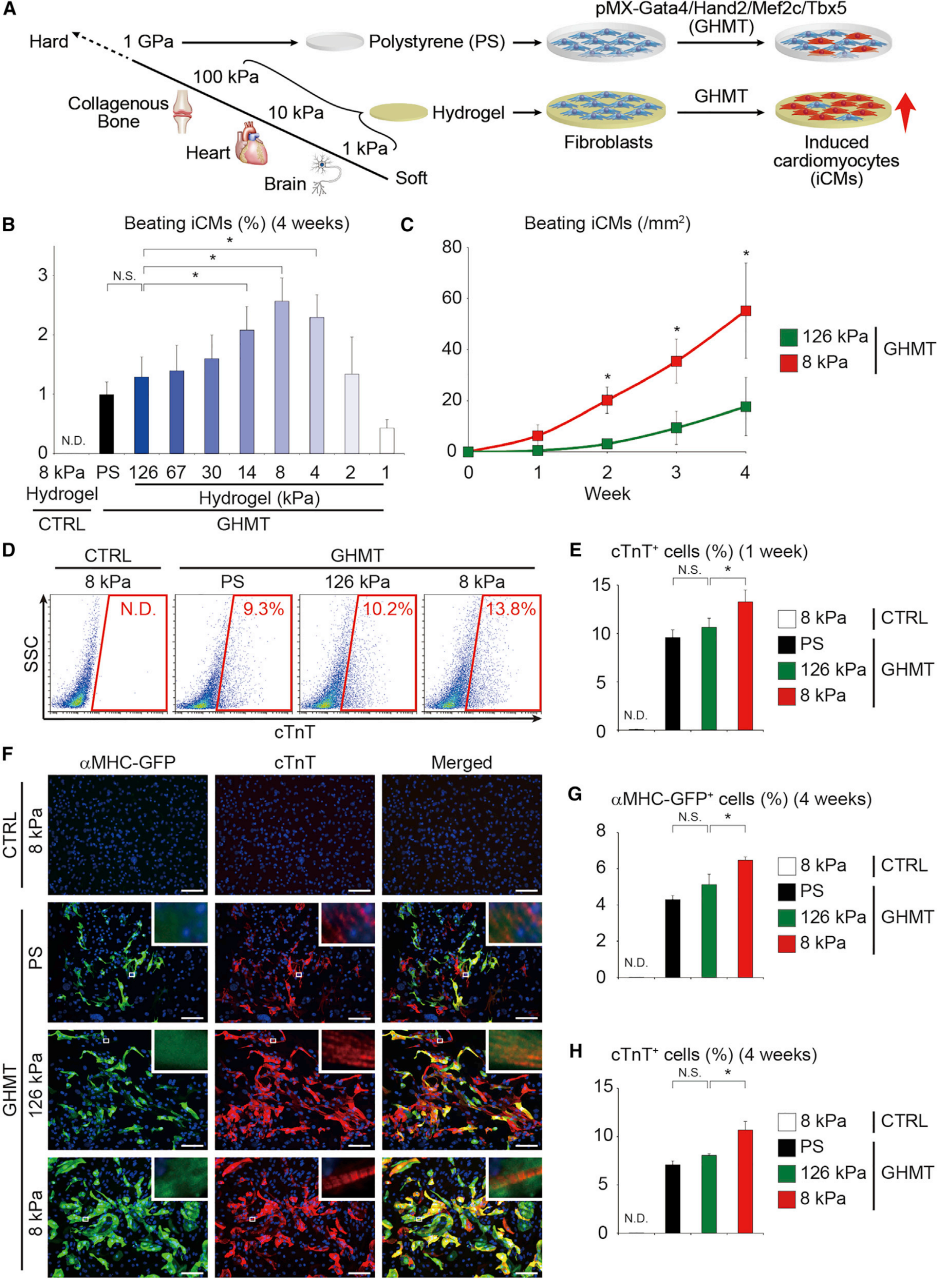
Soft Matrices Promote Cardiac Reprogramming (A) Solid tissues exhibit a range of stiffness, as measured by the elastic modulus. Conventional PS dishes and dishes with Matrigel-based hydrogels of different elasticities (1–126 kPa) were used for cardiac reprogramming. αMHC-GFP mouse fibroblasts were transduced with pMX-GHMT to generate induced cardiomyocytes (iCMs). (B) Fibroblasts were seeded on PS and hydrogels of various elasticities and transduced with GHMT. The effect of substrate elasticity on cardiac reprogramming was analyzed by counting spontaneously beating cells after 4 weeks; n = 3 independent triplicate experiments. (C) Time course of cardiac reprogramming on 126 kPa and 8 kPa hydrogel culture; n = 3 independent triplicate experiments. (D and E) FACS analysis of cTnT expression after 1 week. Fibroblasts were transduced with GHMT and cultured on PS or 126 kPa and 8 kPa substrates. (E) Quantitative data; n = 3 independent triplicate experiments. (F–H) Immunocytochemistry of αMHC-GFP, cTnT, and DAPI after 4 weeks. Cells were treated as described in (D). High-magnification views, as insets, show sarcomeric organization. (G and H) Quantitative data; n = 3 independent triplicate experiments. All data are presented as the means ± SD. ^∗^p < 0.05 versus the relevant control. NS, not significant; ND, not detected; CTRL, control. Scale bars represent 100 μm.

Given that tissue elasticity of the myocardium (10–20 kPa) is much softer than that of PS dishes (∼GPa), we studied the effect of matrix stiffness on cardiac reprogramming (Berry et al., 2006; Engler et al., 2006). We plated fibroblasts from alpha myosin heavy chain (αMHC)-GFP transgenic mice, in which only cardiomyocytes express GFP (Ieda et al., 2010), on Matrigel-coated PS dishes or Matrigel-based hydrogels with different elasticities ranging from 1 kPa (similar to brain) to 126 kPa (similar to collagenous bone). The following day, we transduced the fibroblasts with the retroviral GHMT (pMX-GHMT) to induce cardiac reprogramming (Figure 1A). To identify the effect of ECM stiffness on cardiac reprogramming, we analyzed the number of spontaneously beating iCMs after 4 weeks. We found that soft substrates (4–14 kPa) resulted in a significantly higher number of beating iCMs with a peak at 8 kPa (comparable with the stiffness of native myocardium; Figure 1B, Video S1). Cardiac reprogramming was not induced without GHMT transduction on 8 kPa hydrogels. Consistent with a previous report (Herum et al., 2017), softer substrates (1–2 kPa) altered cell morphology into rounder and smaller shapes, leading to cell detachment and death (Figures 1B and S1A–S1C). Sequential analyses revealed that iCMs cultured on 8 kPa hydrogels started to contract earlier just after 1 week and that the number of beating iCMs increased by ∼3-fold compared with the number of cells on 126 kPa hydrogels after 4 weeks (Figure 1C). Next, we evaluated contraction/relaxation velocities of beating iCMs using the new high-speed video microscopy and motion vector analysis to determine detailed functional properties of iCMs. The contraction/relaxation velocities of iCMs on 8 kPa hydrogels were higher than the velocities on rigid substrates, demonstrating functional maturation of iCMs on soft substrates (Figure S1D). The number of Ca^2+^ transient^+^ cells on 8 kPa hydrogel also increased by ∼2-fold relative to the cells cultured on rigid substrates after 4 weeks (Figure S1E). It was previously reported that all three subtypes of iCMs, such as pacemaker-, atrial-, and ventricular-like cardiomyocytes, were generated by direct cardiac reprogramming (Nam et al., 2014). Therefore, it is possible that soft matrix may induce the pacemaker cells to increase the number of beating cells. qRT-PCR analysis revealed that the expression of *Hcn4*, a marker for the pacemaker cells, was suppressed during culture on 8 kPa hydrogels, suggesting that soft matrix did not increase the induction of pacemaker cells (Figure S1F).

Video S1. Spontaneously Beating GHMT-Induced iCMs Cultured on 8 kPa Hydrogels for 4 Weeks, Related to Figure 1B

Fluorescence-activated cell sorting (FACS) analyses demonstrated that 8 kPa hydrogels induced more cardiac troponin T (cTnT)^+^ cells than did PS or 126 kPa substrates after 1 and 4 weeks (Figures 1D, 1E, and S1H). Immunocytochemistry also revealed that, after 1 week, the number of cTnT^+^ cells increased on 8 kPa substrates relative to that on PS or 126 kPa hydrogels; however, only a few cells exhibited sarcomeric structures at this early stage, consistent with the results of previous studies (Figure S1G) (Ieda et al., 2010; Song et al., 2012). The iCMs showed well-defined sarcomeric structures, and, after 4 weeks, more αMHC-GFP^+^ and cTnT^+^ cells were induced on 8 kPa hydrogels than on PS dishes or 126 kPa hydrogels (Figures 1F–1H). The relative increase in cTnT^+^ cells was ∼1.5-fold, which was less than the increase in beating iCM generation, suggesting that soft substrates promote the degree of iCM maturation. These results demonstrated that soft matrix similar to the myocardial stiffness improved the efficiency and quality of cardiac reprogramming compared with the ECM stiffness of non-cardiac tissue or conventional PS dishes.

### Soft ECM Induces Cardiac Programs and Suppresses YAP/TAZ Signaling

**Figure 2:**
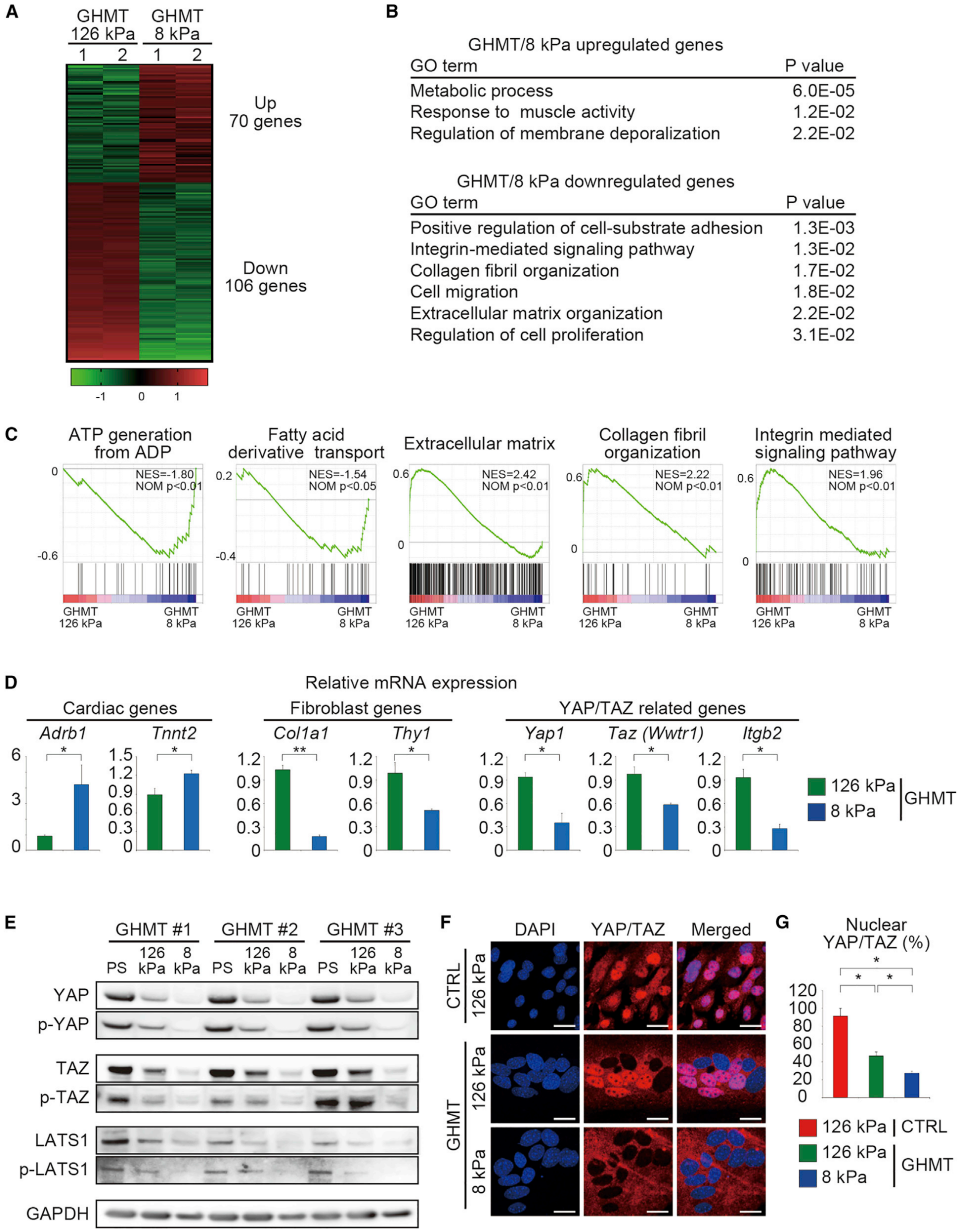
Soft Matrix Suppresses Fibroblast Signature and YAP/TAZ during Cardiac Reprogramming (A) Heatmap of microarray data illustrating the differentially expressed genes between the GHMT-transduced fibroblasts cultured on 126 kPa (GHMT/126 kPa) and 8 kPa (GHMT/8 kPa) hydrogels for 2 weeks; n = 2 independent biological replicates. (B) GO term analysis of the upregulated and downregulated genes in GHMT/8 kPa compared with those of GHMT/126 kPa. Cardiac-related genes were upregulated, whereas fibroblast-related genes were downregulated in GHMT/8 kPa. (C) GSEA for GHMT/8 kPa and GHMT/126 kPa. (D) qRT-PCR analysis to determine relative mRNA expression of cardiomyocyte-, fibroblast-, and YAP/TAZ-related genes in GHMT/8 kPa-cultured cells compared with the GHMT/126 kPa-cultured cells; n = 3 independent triplicate experiments. (E) Western blotting of total YAP, TAZ, LATS1, and phosphorylated YAP (pYAP), TAZ (pTAZ), and LATS1 (pLATS1) protein levels in GHMT-transduced fibroblasts on PS dishes or Matrigel-based hydrogels with different elasticities; n = 3 independent biological replicates. (F and G) Immunocytochemistry of YAP/TAZ and DAPI after 72 h. (G) Quantitative data (n = 3 independent triplicate experiments). Nuclear YAP/TAZ expression was reduced in the presence of GHMT and under 8 kPa culture conditions. All data are presented as the means ± SD. ^∗^p < 0.05; ^∗∗^p < 0.01 versus the relevant control. Scale bars represent 20 μm.

Next, to investigate the underlying mechanisms resulting in significant differences in cardiac reprogramming on 8 kPa and 126 kPa hydrogels, we performed microarray analyses to identify differentially expressed genes between the two groups. Differential gene expression analyses revealed that 70 genes were upregulated in GHMT-transduced fibroblasts on 8 kPa hydrogels, while 106 genes were downregulated at least 2-fold compared with those in the 126 kPa group (Figure 2A). Gene ontology (GO) analyses demonstrated that upregulated genes in cells grown on 8 kPa hydrogels were enriched for GO terms associated with cardiac function, while the downregulated genes were enriched for GO terms associated with fibroblast signatures (such as cell substrate adhesion, migration, and proliferation), ECM organization, and integrin-mediated signaling pathway (Figure 2B). Gene set enrichment analysis (GSEA) also demonstrated that genes related to the ATP generation and fatty acid transport were upregulated in GHMT-transduced fibroblasts cultured on 8 kPa hydrogels, whereas fibroblast-related genes were strongly downregulated in cells cultured on 8 kPa compared with those on 126 kPa hydrogels (Figure 2C). qRT-PCR results confirmed that cardiac genes, such as β1-adrenergic receptor (*Adrb1*) and sarcomeric structure (*Tnnt2*), were upregulated, whereas fibroblast genes (*Col1a1*, *Thy1*) were significantly downregulated in the 8 kPa group (Figure 2D). These results suggested that soft matrices induced cardiac gene programs concomitant with repression of fibroblast signatures.

YAP and TAZ are transcriptional coactivators that are imported into the nuclei when cells are cultured on rigid substrates, whereas they are exported to the cytoplasm, phosphorylated, and degraded via the proteasome system on elastic substrates (Dupont, 2016; Dupont et al., 2011; Ohgushi et al., 2015). Western blotting revealed that protein levels of total YAP and TAZ (nuclear and cytoplasmic localization) and phosphorylated YAP and TAZ (cytoplasmic localization) were lower in the GHMT-transduced cells cultured on soft ECM than on rigid substrates (Figure 2E). Consistent with these findings, qRT-PCR showed that gene expression levels of *Yap1*, *Taz (Wwtr1)*, and the YAP/TAZ target gene *Itgb2* were significantly lower in the GHMT-transduced cells cultured on 8 kPa than on 126 kPa hydrogels (Figure 2D). The canonical Hippo pathway, activated by contact inhibition, suppresses YAP and TAZ through the phosphorylation of large tumor suppressor homolog 1 (LATS1) (Zhao et al., 2007). Therefore, if the canonical Hippo signaling was responsible for YAP/TAZ suppression on 8 kPa substrate, LATS1 would be phosphorylated. However, western blotting demonstrated that the levels of both total and phosphorylated LATS1 decreased on 8 kPa hydrogel, suggesting that the canonical Hippo signaling might not be the primary regulator of YAP/TAZ in this particular model (Figure 2E). IHC revealed that most fibroblasts expressed YAP/TAZ in the nuclei on 126 kPa substrates under basal conditions (Figure 2F). After 3 days on 126 kPa substrate and with GHMT transduction, nuclear YAP/TAZ expression was reduced by ∼50%, which was further reduced on 8 kPa culture in the presence of GHMT (Figures 2F and 2G). These results suggest that elastic matrices similar to the myocardium promote cardiac reprogramming concomitant with suppression of fibroblast signatures and YAP/TAZ signaling.

### Inhibition of YAP/TAZ Promotes Cardiac Reprogramming and Suppresses Fibroblast Signatures

**Figure 3:**
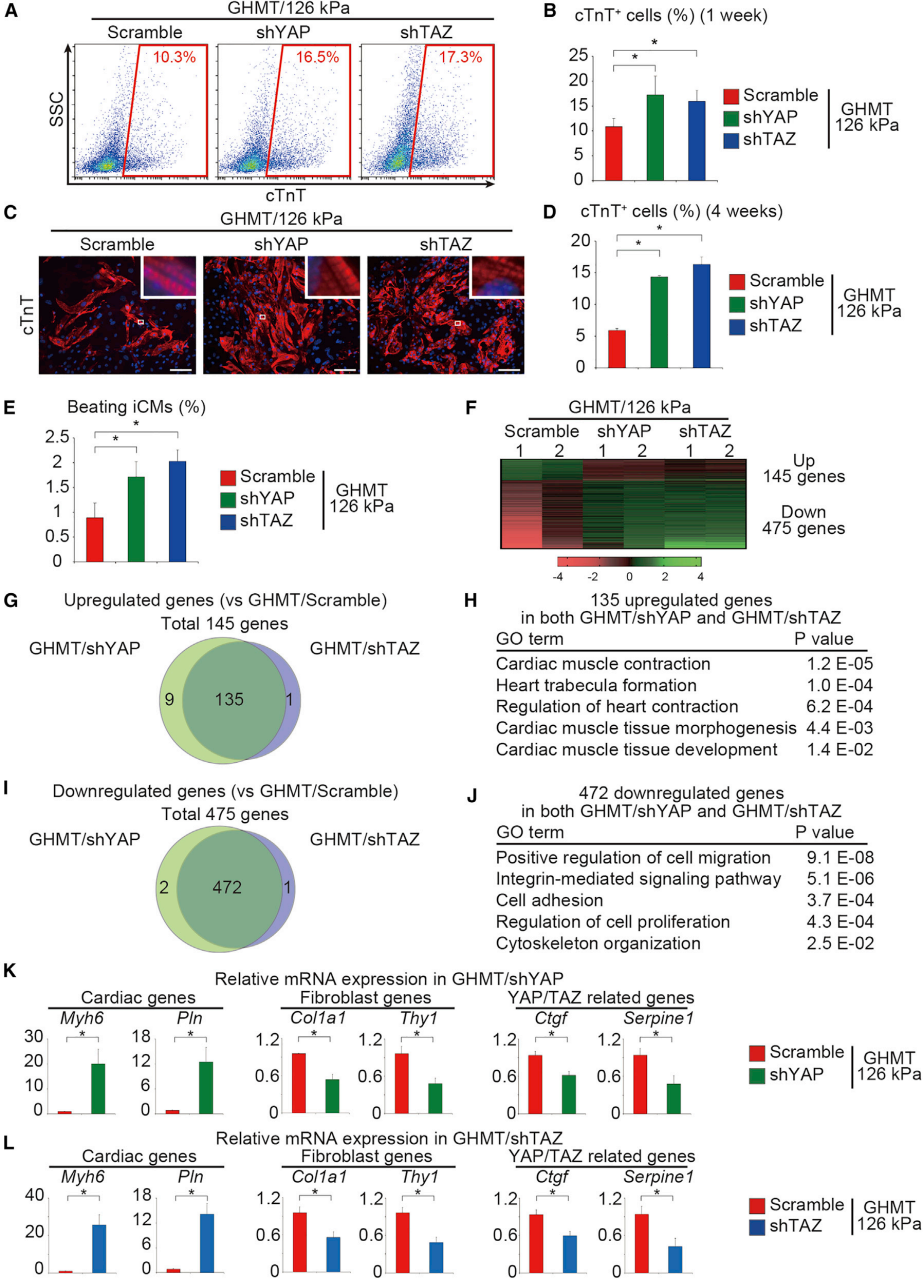
Inhibition of YAP/TAZ Enhances Cardiac Reprogramming and Suppresses Fibroblast Signature (A and B) FACS analysis of cTnT expression after 1 week. GHMT-transduced fibroblasts cultured on 126 kPa hydrogels were transduced with Scramble shRNA and YAP and TAZ-specific shRNA (shYAP and shTAZ). (B) Quantitative data; n = 3 independent triplicate experiments. (C and D) Immunocytochemistry of cTnT and DAPI after 4 weeks. Cells were treated as described in (A). High-magnification views, as insets, show sarcomeric organization. (D) Quantitative data; n = 3 independent triplicate experiments. (E) Quantitative data for the number of spontaneously beating cells after 4 weeks; n = 3 independent triplicate experiments. (F) Heatmap of microarray data illustrating the global gene expression pattern of fibroblasts 2 weeks after transduction with GHMT/scramble shRNA, GHMT/shYAP, or GHMT/shTAZ cultured on 126 kPa hydrogels. Differentially expressed genes are shown; n = 2 independent biological replicates. (G and H) Venn diagram showing genes upregulated after GHMT/shYAP and GHMT/shTAZ transduction by more than 2-fold compared with that of GHMT/scramble shRNA. (H) GO analysis of the 135 upregulated genes of the GHMT/shYAP and GHMT/shTAZ groups. Cardiac-related GO terms are shown. (I and J) Venn diagram showing genes downregulated after GHMT/shYAP and GHMT/shTAZ transduction by more than 2-fold compared with that of GHMT/scramble shRNA. (J) GO analysis of the 472 downregulated genes in both GHMT/shYAP and GHMT/shTAZ groups. Fibroblast-related GO terms are shown. (K and L) qRT-PCR analysis to determine relative mRNA expression of cardiomyocyte, fibroblast, and YAP/TAZ target genes in the (K) GHMT/shYAP and (L) GHMT/shTAZ groups compared with GHMT/scramble shRNA groups; n = 3 independent triplicate experiments. All experiments were performed on 126 kPa hydrogels. All data are presented as the means ± SD. ^∗^p < 0.05 versus the relevant control. Scale bars represent 100 μm.

Our results showed that YAP/TAZ signaling was inhibited in soft ECM-mediated cardiac reprogramming; however, it is unknown whether this suppression improves iCM reprogramming. To test this, we suppressed YAP and TAZ expression with specific short hairpin RNAs (shRNAs) (shYAP and shTAZ) in GHMT-transduced fibroblasts on 126 kPa hydrogels (Table S1). Downregulation of YAP and TAZ expression by shYAP and shTAZ, respectively, was confirmed by qRT-PCR and western blotting (Figures S2A and S2B). FACS analyses revealed that induction of cTnT^+^ cells significantly increased after treatment with shYAP or shTAZ more than with scramble shRNA on 126 kPa and PS dishes after 1 week (Figures 3A, 3B, S2C, and S2D). Knockdown of both YAP and TAZ did not further increase cardiac reprogramming (data not shown). Other shRNAs (shYAP#2 or shTAZ#2) also increased cardiac reprogramming, demonstrating the specific and reproducible effects of shYAP and shTAZ on cardiac reprogramming (Figures S2E and S2F, and Table S1). Immunocytochemistry of cTnT also demonstrated that YAP and TAZ knockdown increased the number of cTnT^+^ cells with well-defined sarcomeric structures after 4 weeks (Figures 3C and 3D). Moreover, the number of beating iCMs also increased after shYAP and shTAZ treatment (Figure 3E).

We next performed microarray analyses to determine the molecular mechanism of shYAP and shTAZ-mediated cardiac reprogramming on 126 kPa hydrogels (Figure 3F). Compared with scramble shRNA, differential gene expression analyses revealed that, after either shYAP or shTAZ treatment, 145 genes were upregulated and 475 genes were downregulated with significant overlaps between the two shRNA groups, suggesting that shYAP and shTAZ largely target the same pathways (Figures 3F–3J). GO analyses showed that upregulated genes in the shYAP and shTAZ treatments were enriched for GO terms associated with cardiac function and development, while downregulated genes were enriched for GO terms associated with fibroblast signatures (cell migration, adhesion, and proliferation; Figures 3H and 3J). qRT-PCR analysis confirmed that inhibition of YAP and TAZ expression upregulated a panel of cardiac genes and significantly downregulated fibroblast and YAP/TAZ target genes, such as *Ctgf* and *Serpine1* (Figures 3K and 3L) (Dupont et al., 2011). Thus, inhibition of YAP/TAZ promoted cardiac reprogramming concomitant with suppression of fibroblast signatures, recapitulating the effect of soft substrate-mediated cardiac reprogramming.

### Activation of YAP/TAZ Suppresses Soft Substrate-Mediated Cardiac Reprogramming

**Figure 4:**
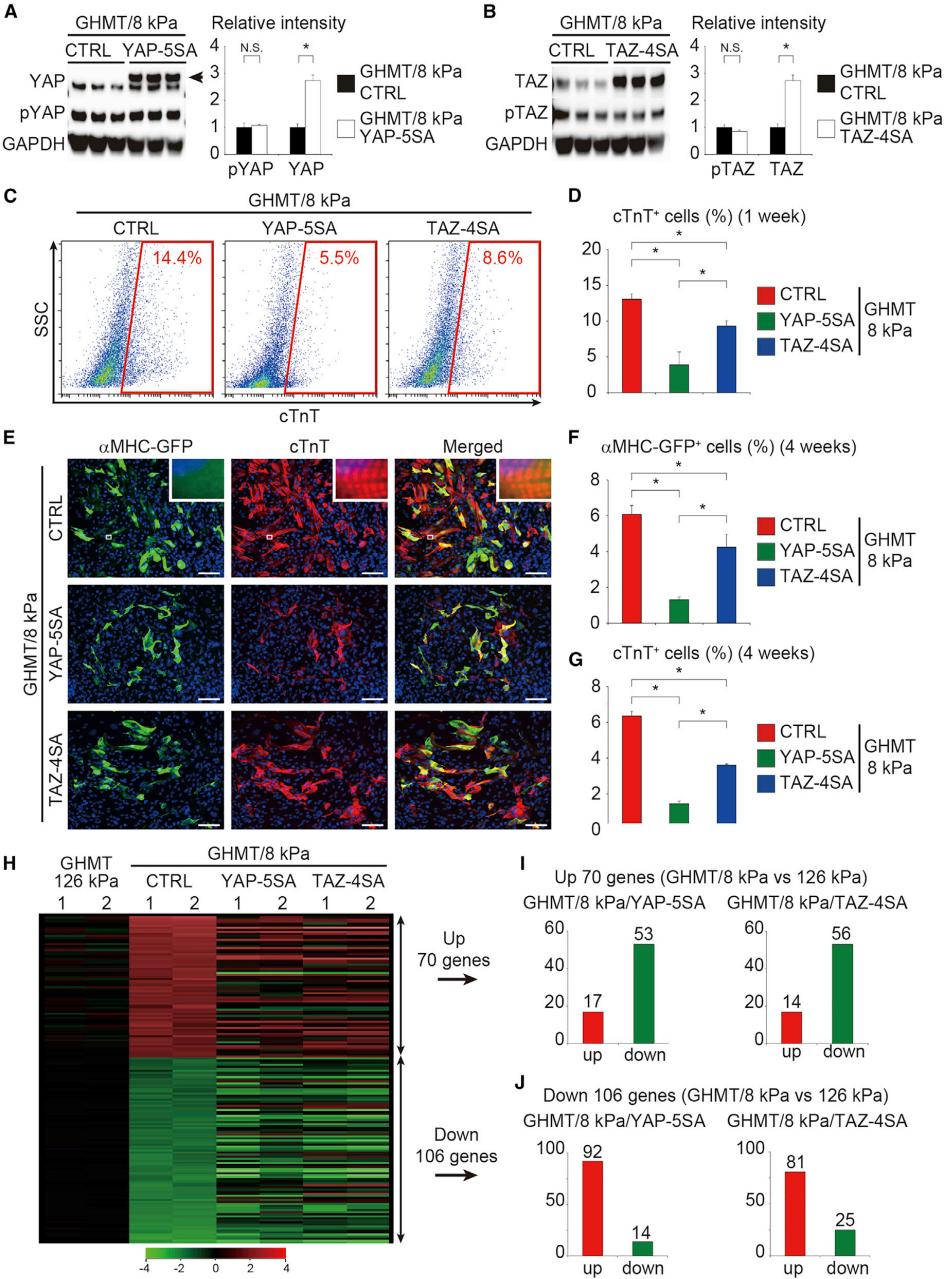
Activation of YAP/TAZ Inhibits Soft Matrix-Mediated Cardiac Reprogramming (A and B) Western blot analysis of total YAP and TAZ, pYAP, and pTAZ levels in GHMT-transduced fibroblasts treated with (A) YAP-5SA and (B) TAZ-4SA for 72 h (n = 3 independent triplicate experiments). Arrow indicates exogenous YAP expression. Relative intensity was normalized to the levels in GHMT-transduced fibroblasts cultured on 8 kPa hydrogels. (C and D) FACS analysis of cTnT expression after 1 week. GHMT-induced fibroblasts cultured on 8 kPa hydrogels were transduced with YAP-5SA and TAZ-4SA. YAP-5SA and TAZ-4SA suppressed the generation of iCMs. (D) Quantitative data; n = 3 independent triplicate experiments. (E–G) Immunocytochemistry of αMHC-GFP, cTnT, and DAPI after 4 weeks. High-magnification views, as insets, show sarcomeric organization. (F and G) Quantitative data; n = 3 independent triplicate experiments. (H–J) Heatmap of microarray data illustrating the global gene expression pattern of fibroblasts 2 weeks after transduction with GHMT on 126 kPa (GHMT/126 kPa), GHMT on 8 kPa (GHMT/8 kPa/CTRL), GHMT/YAP-5SA on 8 kPa (GHMT/8 kPa/YAP-5SA), and GHMT/TAZ-4SA on 8 kPa (GHMT/8 kPa/TAZ-4SA) hydrogels (n = 2 independent biological replicates). Gene expression changes induced by culturing on 8 kPa hydrogels were largely reversed by overexpression of (I) YAP-5SA and (J)TAZ-4SA. All data are presented as the means ± SD. ^∗^p < 0.05 versus the relevant control. Scale bars represent 100 μm.

To determine whether soft substrates promote cardiac reprogramming through inhibition of YAP/TAZ, we tested whether activation of YAP/TAZ could counteract the effect of soft ECM-mediated cardiac reprogramming. We overexpressed YAP-5SA and TAZ-4SA, which are constitutive active mutants of YAP and TAZ, in GHMT-transduced fibroblasts plated on soft substrates (8 kPa) (Dupont et al., 2011; Moroishi et al., 2016; Zhao et al., 2007). Western blot analyses confirmed that total YAP and TAZ protein expression was significantly upregulated in the cells transduced with YAP-5SA and TAZ-4SA, respectively, whereas phosphorylated YAP and TAZ expression was unaltered, indicating activation of YAP/TAZ signaling in nuclei with these mutants (Figures 4A, 4B, and S2B). FACS analyses demonstrated that activation of YAP/TAZ strongly decreased the induction of cTnT^+^ cells on 8 kPa hydrogels after 1 week (Figures 4C and 4D). Immunocytochemistry of αMHC-GFP and cTnT also demonstrated that overexpression of YAP-5SA and TAZ-4SA suppressed the induction of αMHC-GFP^+^ and cTnT^+^ cells after 4 weeks (Figures 4E–4G). Furthermore, expression of activated YAP/TAZ inhibited the generation of beating iCMs after 4 weeks, overriding the positive effects of soft ECM-mediated cardiac reprogramming (Figure S3A). Next, we performed the 5-ethynyl-2′-deoxyuridine (EdU) incorporation assay to determine cell proliferation during cardiac reprogramming. A 2-week pulse labeling with EdU demonstrated that almost all iCMs were post-mitotic and YAP-5SA and TAZ-4SA did not augment iCM proliferation (Figures S3B and S3C). We next examined whether overexpression of YAP-5SA and TAZ-4SA can globally counteract soft ECM-mediated gene regulation. Microarray analyses revealed that 53 and 56 out of 70 genes upregulated by 8 kPa hydrogels were suppressed, while 92 and 81 out of 106 downregulated genes were upregulated by the overexpression of YAP-5SA and TAZ-4SA, respectively (Figures 4H–4J); this suggests that transcriptional changes affected by soft ECM were largely mediated via suppression of YAP/TAZ signaling. These results indicate that soft ECM promotes cardiac reprogramming at least in part via suppression of YAP/TAZ signaling.

### Integrin, Rho/ROCK, Actomyosin, and YAP/TAZ Signaling Are Suppressed in iCMs Cultured on Soft Substrates

We next investigated the upstream mechanotransduction pathway regulating YAP/TAZ activity in soft ECM-induced cardiac reprogramming. It has been reported that integrin receptors, Rho/Rho-associated protein kinase (ROCK), actomyosin organization, and YAP/TAZ signaling are activated when cells are cultured on hard substrates, yet are suppressed when cells are cultured on soft substrates (Dupont, 2016; Dupont et al., 2011). Consistent with this, microarray and qRT-PCR analyses revealed that genes related to integrin-mediated signaling and *Itgb2* were downregulated in GHMT-transduced cells cultured on 8 kPa hydrogels more than those on 126 kPa (Figures 2B and 2D). Furthermore, formation of phalloidin^+^ stress fibers (F-actin, activated actin) was disrupted in cells cultured on 8 kPa hydrogels, concomitant with a reduction of YAP/TAZ nuclear localization (Figure S4A). Thus, integrin signaling, actomyosin organization, and YAP/TAZ activity were suppressed during cardiac reprogramming by culturing the cells on 8 kPa soft ECM.

**Figure 5:**
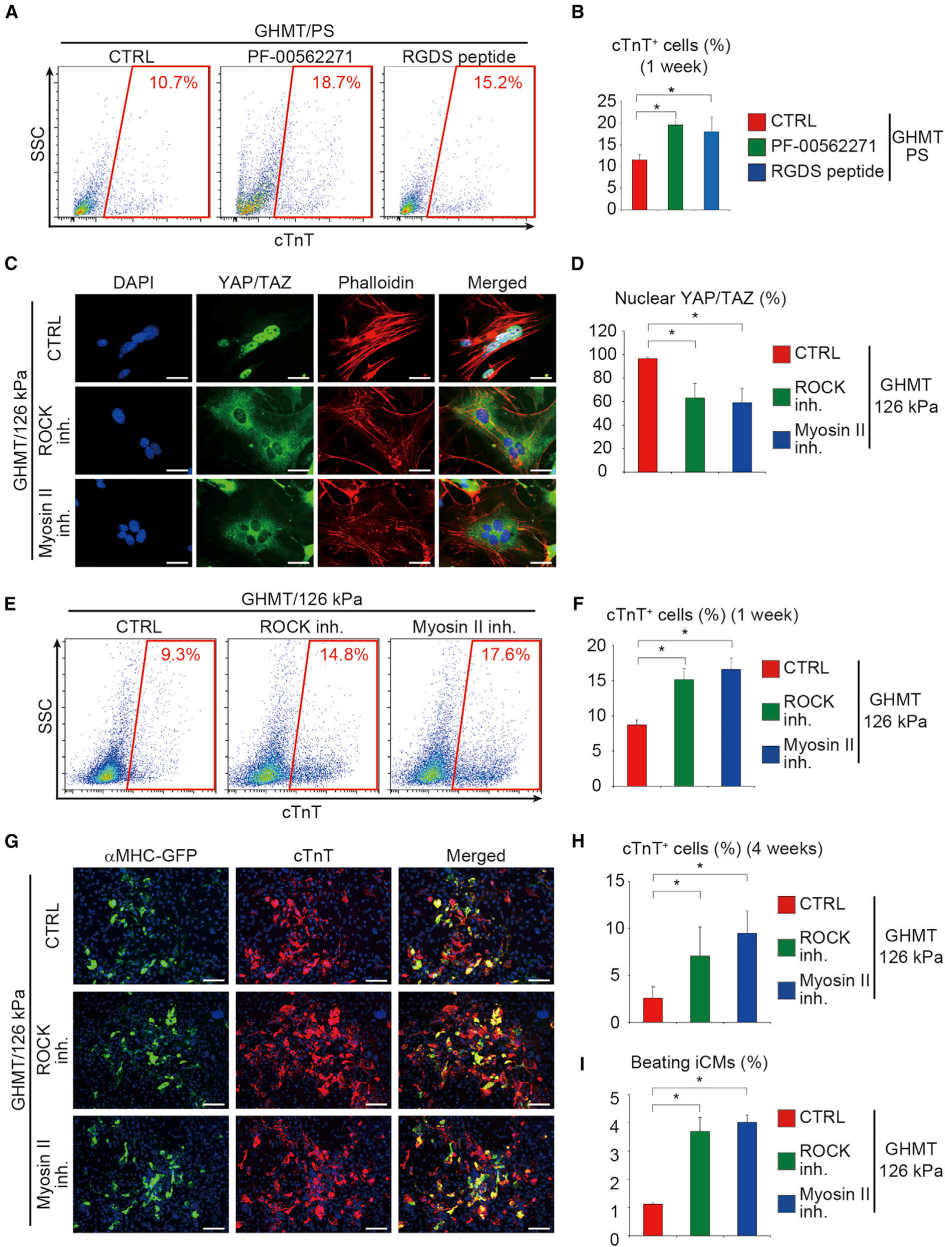
Inhibition of Integrin Signaling, ROCK, and Myosin II Promotes Cardiac Reprogramming on Rigid Substrates (A and B) FACS analysis of cTnT expression after 1 week of culture. GHMT-induced fibroblasts cultured on PS dishes were treated with integrin signaling inhibitors. (B) Quantification of data presented in (A); n = 3 independent triplicate experiments. (C and D) Immunocytochemistry of YAP/TAZ, phalloidin, and DAPI. GHMT-transduced fibroblasts were treated with ROCK and myosin II inhibitors, cultured on 126 kPa hydrogels, and analyzed 3 h after inhibitor treatment. (D) Quantitative data; n = 4 independent triplicate experiments. (E and F) FACS analysis of cTnT expression after 1 week. Cells were treated as described in (C). (F) Quantitative data; n = 3 independent triplicate experiments. (G and H) Immunocytochemistry of αMHC-GFP, cTnT, and DAPI after 4 weeks. The ROCK inhibitor Y-27632 was used to supplement the medium for 4 weeks, whereas the myosin II inhibitor blebbistatin was used to supplement the medium for the first 2 weeks. (H) Quantitative data; n = 3 independent triplicate experiments. (I) Quantitative data of the number of spontaneously beating cells after 4 weeks. Cells were treated as described in (C); n = 4 independent triplicate experiments. All data are presented as the means ± SD. ^∗^p < 0.05 versus the relevant control. inh, inhibitor. Scale bars represent 20 μm (C), 100 μm (G).

Next, to determine the role of integrin, Rho/ROCK, and actomyosin signaling in cardiac reprogramming, we treated GHMT-transduced fibroblasts on 126 kPa substrates with specific inhibitors of integrin signaling (RGDS peptide, PF-00562271), ROCK (Y-27632), and myosin II (blebbistatin). Inhibition of integrin signaling with RGDS peptide and PF-00562271 significantly increased the number of cTnT^+^ cells, as shown by FACS analysis (Figures 5A and 5B). Blebbistatin is a reversible inhibitor of all myosin II isoforms that are expressed in striated muscle (including CMs), smooth muscle, and non-muscle cells (including fibroblasts) (Yahalom-Ronen et al., 2015). Consistent with the critical function of Rho/ROCK and myosin II in cytoskeleton tension, both inhibitors disrupted phalloidin^+^ stress fibers and led to reduced YAP/TAZ nuclear localization in GHMT-transduced fibroblasts (Figures 5C and 5D). FACS analyses demonstrated that cTnT^+^ cells were significantly increased with the inhibitor treatment after 1 week, suggesting that inhibition of the Rho/ROCK and actomyosin pathways promotes cardiac reprogramming on 126 kPa and PS substrates (Figures 5E, 5F, S4B, and S4C). In contrast, these inhibitors did not significantly increase cardiac reprogramming on 8 kPa hydrogels, suggesting that the effects of these inhibitors are mainly dependent on matrix stiffness (Figures S4D and S4E). Inhibition of ROCK and myosin II for the first 2 weeks was sufficient to increase the generation of αMHC-GFP^+^ and cTnT^+^ cells and beating iCMs by ∼2- to 3-fold after 4 weeks on 126 kPa hydrogels (Figures 5G–5I). EdU assay showed that inhibition of ROCK and myosin II did not increase iCM proliferation (Figures S4F and S4G), suggesting that these inhibitors promoted cardiac reprogramming mainly by modifying the iCM differentiation process. Sustained blebbistatin treatment for 4 weeks suppressed the generation of beating iCMs, consistent with the fact that cardiomyocyte contraction requires active cardiac myosin II (Lyon et al., 2015; Yahalom-Ronen et al., 2015). Thus, transient blebbistatin treatment targeted non-muscle myosin II in fibroblasts and promoted cardiac reprogramming. These results suggest that inhibition of the integrin, Rho/ROCK, and actomyosin pathway promoted cardiac reprogramming with suppression of YAP/TAZ signaling.

### Inhibition of Rho/ROCK and Actomyosin Promotes Cardiac Reprogramming via Suppression of YAP/TAZ

**Figure 6:**
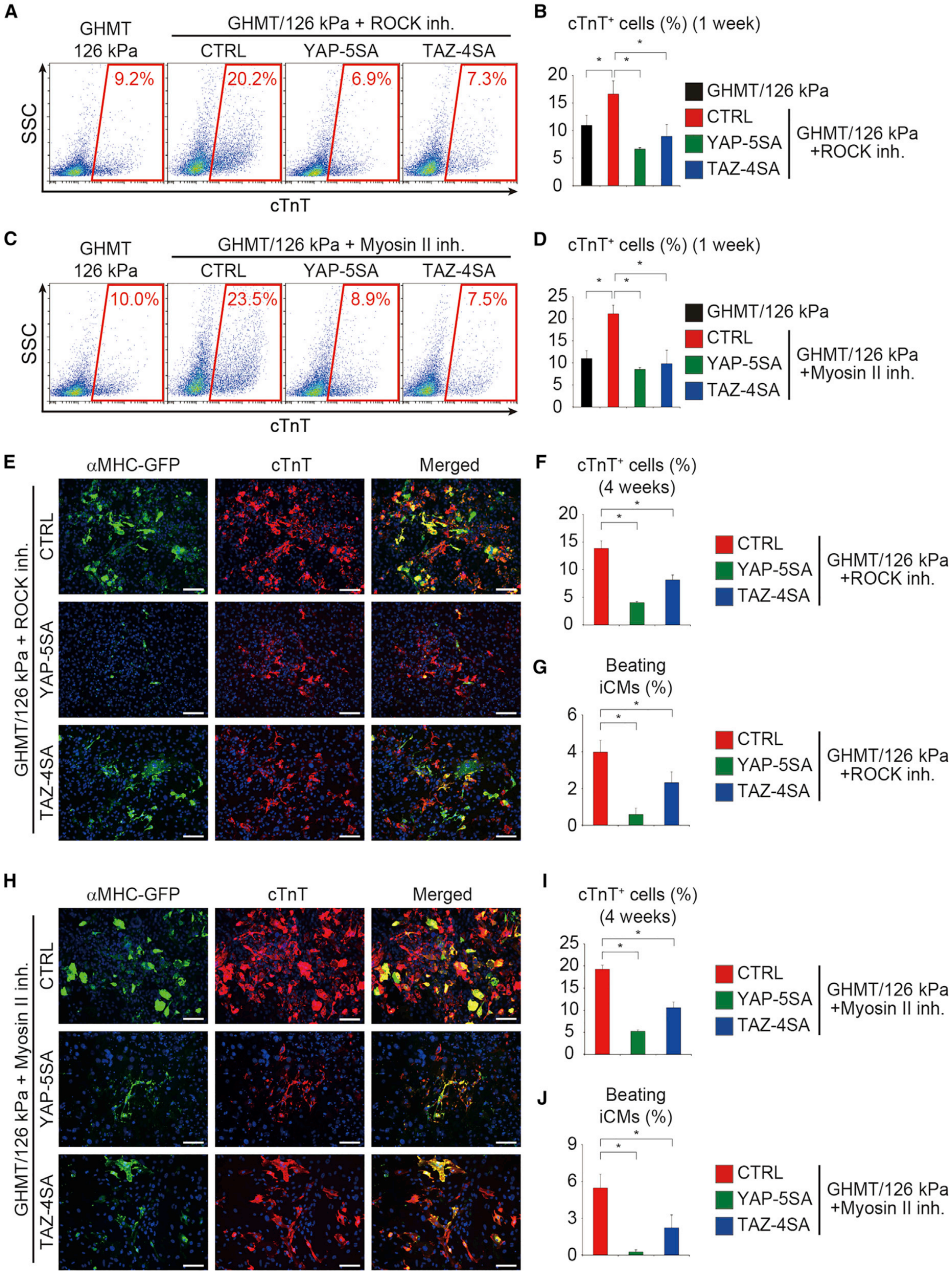
Activation of YAP/TAZ Abolished the Effects of ROCK and Myosin II Inhibitors on Cardiac Reprogramming (A and B) FACS analysis of cTnT expression after 1 week. GHMT-transduced cells cultured on 126 kPa hydrogels and treated with ROCK inhibitor were transduced with YAP-5SA and TAZ-4SA. Activation of YAP/TAZ suppressed ROCK inhibitor-mediated cardiac reprogramming. (B) Quantitative data; n = 3 independent triplicate experiments. (C and D) FACS analysis of cTnT expression after 1 week. GHMT-transduced cells cultured on 126 kPa hydrogels and treated with myosin II inhibitor were transduced with YAP-5SA and TAZ-4SA. Activation of YAP/TAZ suppressed myosin II inhibitor-mediated cardiac reprogramming. (D) Quantitative data; n = 3 independent triplicate experiments. (E, F, H, and I) Immunocytochemistry of αMHC-GFP, cTnT, and DAPI after 4 weeks. GHMT-fibroblasts cultured on 126 kPa hydrogels and treated with ROCK inhibitor for 4 weeks (E and F) and myosin II inhibitor for first 2 weeks (H and I) were transduced with YAP-5SA and TAZ-4SA. (F and I) Quantitative data; n = 3 independent triplicate experiments. (G and J) Quantitative data of the number of spontaneously beating cells after 4 weeks (n = 4 independent triplicate experiments). Activation of YAP/TAZ suppressed ROCK (G) and myosin II inhibitor (J)-mediated cardiac reprogramming. All experiments were performed on 126 kPa hydrogels. All data are presented as the means ± SD. ^∗^p < 0.05 versus the relevant control. Scale bars represent 100 μm.

Next, to investigate whether inhibition of Rho/ROCK and actomyosin promotes cardiac reprogramming through suppression of the downstream targets YAP/TAZ, we asked whether constitutive activation of YAP/TAZ overrides the positive effects of ROCK or myosin II inhibitor-induced cardiac reprogramming. FACS analyses demonstrated that expression of activated YAP and TAZ suppressed the generation of cTnT^+^ cells in either ROCK or myosin II inhibitor-treated cells on 126 kPa after 1 week (Figures 6A–6D). Immunocytochemistry also demonstrated that activation of YAP or TAZ suppressed ROCK or myosin II inhibitor-mediated cardiac reprogramming after 4 weeks (Figures 6E, 6F, 6H, and 6I). Consistently, the number of beating iCMs was also reduced after transduction with YAP-5SA or TAZ-4SA, overriding the positive effects of ROCK and myosin II inhibitors on cardiac reprogramming (Figures 6G and 6J). Expression of the constitutively active YAP/TAZ also inhibited cardiac reprogramming of fibroblasts cultured on 8 kPa hydrogels and treated with ROCK and myosin II inhibitors (Figures S5A–S5J). These results demonstrate that inhibition of the Rho/ROCK and actomyosin pathways promotes cardiac reprogramming via suppression of YAP/TAZ.

### Soft ECM Promoted Sendai Virus Vector-Mediated Cardiac Reprogramming via Suppression of YAP/TAZ

**Figure 7:**
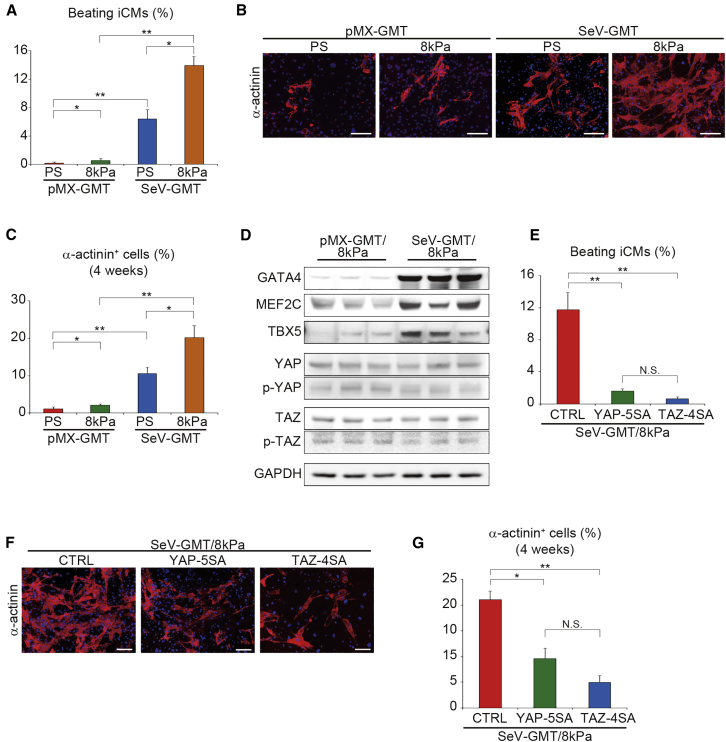
Soft Matrix Promoted SeV-Mediated Cardiac Reprogramming via YAP/TAZ Suppression (A) Quantification of the spontaneously beating iCMs after 4 weeks of culture (n = 4 independent triplicate experiments). Fibroblasts were seeded on PS or 8 kPa hydrogels and transduced with pMX-GMT or SeV-GMT. Soft substrate enhanced SeV-mediated cardiac reprogramming. (B) Immunocytochemistry of α-actinin and DAPI after 4 weeks. Cell were treated as shown in (A). (C) Quantification of data presented in (B); n = 3 independent triplicate experiments. (D) Western blot analysis for GMT, YAP/TAZ, and pYAP/pTAZ protein expression in pMX-GMT or SeV-GMT-transduced fibroblasts cultured on 8 kPa hydrogels; n = 3 independent biological replicates. (E) Quantification of the number of spontaneously beating cells after 4 weeks; n = 4 independent triplicate experiments. Activation of YAP/TAZ suppressed SeV-mediated cardiac reprogramming. (F) Immunocytochemistry of α-actinin and DAPI after 4 weeks. (G) Quantification of data presented in (F); n = 3 independent triplicate experiments. All data are presented as the mean ± SD. ^∗^p < 0.05; ^∗∗^p < 0.01 versus the corresponding control. Scale bars represent 100 μm.

Thus far, we demonstrated that soft ECM and suppression of YAP/TAZ promoted cardiac reprogramming induced by the retroviral vectors expressing GHMT. It has been reported that Sendai virus (SeV) vectors reprogrammed fibroblasts on rigid PS dishes into integration-free iCMs more efficiently than the pMX retrovirus vectors (Miyamoto et al., 2018). Therefore, to test the universality of soft substrate effects and YAP/TAZ signaling on cardiac reprogramming, we investigated whether 8 kPa soft ECM would promote cardiac reprogramming using the SeV vector system expressing GMT (SeV-GMT). We found that, on PS dishes, SeV-GMT induced a higher number of spontaneously beating iCMs than pMX-GMT, and the 8 kPa substrate further increased the SeV-mediated cardiac reprogramming efficiency up to ∼15% (Figure 7A). Immunocytochemistry also demonstrated that, on PS dishes, SeV-GMT generated more α-actinin^+^ cells than pMX-GMT, and this increase was further enhanced by culturing on soft ECM (Figures 7B and 7C). Western blotting confirmed that fibroblasts cultured on 8 kPa hydrogels and infected with SeV-GMT had higher GMT protein levels than the cells infected with pMX-GMT. The total and phosphorylated YAP and TAZ protein expression was suppressed in both groups compared with the cells on PS dishes (Figures 2E and 7D). Next, to determine whether soft ECM promoted SeV-GMT-mediated cardiac reprogramming by suppressing YAP/TAZ, we overexpressed YAP-5SA and TAZ-4SA in SeV-GMT-transduced fibroblasts plated on 8 kPa hydrogel. After 4 weeks, constitutively active YAP/TAZ inhibited the generation of beating iCMs, overriding the positive effects of soft ECM-mediated cardiac reprogramming by SeV-GMT (Figure 7E). Immunocytochemistry for α-actinin further confirmed that overexpression of YAP-5SA and TAZ-4SA suppressed the generation of iCMs (Figures 7F and 7G). Therefore, soft ECM promoted SeV-GMT-mediated cardiac programming by suppressing YAP/TAZ signaling.

## Discussion

We developed a Matrigel-based hydrogel culture system that recapitulates the stiffness of native tissues to dissect the role of biophysical cues in cardiac reprogramming. Our results demonstrated, for the first time, that soft matrices comparable with native myocardium improved cardiac reprogramming by suppressing the YAP/TAZ signaling and silencing fibroblast gene programs, which were activated by conventional culture on rigid substrates. Thus, we identified a new link between mechanobiology and direct cardiac reprogramming, which may advance our understanding of cellular biology and optimize biomaterials for clinical translation.

Previous *in vitro* cardiac reprogramming studies mostly used PS dishes, which were much stiffer than native myocardium (Addis et al., 2013; Ieda et al., 2010; Ifkovits et al., 2014; Jayawardena et al., 2012; Mohamed et al., 2017; Muraoka et al., 2014; Sadahiro et al., 2015; Song et al., 2012; Srivastava and Ieda, 2012; Wada et al., 2013; Yamakawa et al., 2015; Zhao et al., 2015; Zhou et al., 2015). Thus, our hydrogel culture system was necessary for identifying optimal matrix stiffness and revealing the roles of biophysical factors in cardiac reprogramming. The preferred modulus for cardiac reprogramming identified—8 kPa—is near the value of the elastic modulus of adult rat heart (∼10 kPa) (Berry et al., 2006; Engler et al., 2008; Herum et al., 2017), perhaps indicating that physiologically relevant substrate is most appropriate for cardiac reprogramming. Indeed, softer substrates (1–2 kPa) detached the GHMT-transduced iCMs from culture dishes, whereas harder substrates (∼100 kPa) suppressed cardiac reprogramming via activation of YAP/TAZ and fibroblastic programs (Herum et al., 2017). We anticipate that our culture system will be equally useful in identifying optimal elasticity and key factors for other direct lineage conversions from fibroblasts, such as hepatocytes, neurons, and chondrocytes (Sadahiro et al., 2015; Srivastava and DeWitt, 2016).

Mechanistically, we found that soft substrates promoted cardiac reprogramming, at least in part, through inhibition of integrin, Rho/ROCK, actomyosin, and YAP/TAZ signaling and subsequent suppression of fibroblast programs. We also found that the canonical Hippo/LATS cascade does not appear to be involved in regulating YAP/TAZ activity in soft ECM-mediated cardiac reprogramming. Nuclear YAP/TAZ form complexes with the major transcription factor partner TEADs (the TEAD/TEF family transcription factors) to coactivate gene expression (Lin et al., 2017). Consistent with our results, a recent genome-wide study revealed that chromatin signatures at TEADs-binding loci rapidly changed from open to closed status during cardiac reprogramming (Stone et al., 2019), suggesting that the YAP/TAZ and TEADs pathway is suppressed during cardiac reprogramming. The experiments using SeV vectors revealed that soft ECM improved the efficiency of cardiac reprogramming up to ∼15%, in part by suppressing YAP/TAZ, demonstrating the universality of the effects of soft ECM and YAP/TAZ signaling on cardiac reprogramming. Robust transgene expression also contributed to the efficient cardiac reprogramming using the SeV vectors, suggesting that the balance between the cardiogenic and fibroblast programs determines the efficiency of cardiac reprogramming. It is also possible that mechanical stimulation, actomyosin tension, and Rho/ROCK signaling regulate cardiac reprogramming not only via YAP/TAZ but also through other mechanisms, including myocardin-related transcription factors/serum response factor (SRF) and epigenetic remodeling (Crowder et al., 2016; Dupont et al., 2011; Olson and Nordheim, 2010; Sia et al., 2016). Further studies may provide new insights into the mechanism of iCM generation and improve the efficiency and quality of cardiac reprogramming.

A recent paper showed that, compared with GMT alone, delivery of small molecules (transforming growth factor β and Wnt inhibitors) in the presence of GMT improved *in vivo* cardiac reprogramming and enhanced cardiac repair after MI in mice (Mohamed et al., 2017). The stiffness of infarcted myocardium becomes several-fold harder (∼50 kPa) than healthy myocardium due to the accumulation of newly synthesized ECM and fibrosis (Berry et al., 2006; Engler et al., 2008). Our results suggest that a matrix stiffer than 8 kPa may inhibit cardiac reprogramming. Thus, it would be interesting to determine whether inhibition of Rho/ROCK-actomyosin-YAP/TAZ signaling and fibrolytic treatment may promote cardiac reprogramming *in vivo* and improve cardiac function after MI.

In summary, we developed a Matrigel-based hydrogel culture system to determine the effects of physical cues and mechanotransduction on cardiac reprogramming. Recapitulating *in vivo* environments using biomaterials will allow for identification of new targets to advance cardiac reprogramming technology toward clinical applications.

## Experimental Procedures

### Matrigel-Based Hydrogel Culture System

Polyacrylamide gel solutions were prepared with different concentrations of acrylamide (AAm; 00809-85; Nacalai Tesque, Kyoto, Japan) and N,N′-methylenebisacrylamide (BIS; 22402-02; Nacalai Tesque) to vary elasticity(Yip et al., 2013). For conjugation of Matrigel (356230; Corning Inc., Corning, NY) components, 6-acrylamidohexanoic acid (ACA; A1896; Tokyo Chemical Industry, Tokyo, Japan) solution (500 mM, pH 7) was added to the AAm-BIS mixture to a final concentration of 100 mM. AAm and BIS concentrations and the corresponding Young's modulus are shown in Table S3. Polymerization was initiated with 0.05% ammonium peroxodisulfate (APS; 02627-21; Nacalai Tesque) and catalyzed with 0.2% N,N,N′,N′-tetramethylethylenediamine (TEMED; 33401-72; Nacalai Tesque). The polymerizing solutions (1 mL) were gently poured into the gap of slide glasses (76 mm × 52 mm; S092240; Matsunami; Osaka, Japan) spaced with a 0.3-mm silicone membrane (SR-50; Tigers Polymer Association, Osaka, Japan). After polymerization, the gels were fully hydrated in 0.1 M 2-(N-morpholino) ethanesulfonic acid (MES) buffer (pH 6.1; 21623-26; Nacalai Tesque) overnight. Next, the gels were cut to the same size as PS dish bottoms. To conjugate the Matrigel components to the gel surface, carboxyl groups of the copolymerized ACA were activated with 0.5 M N-hydroxysuccinimide (NHS; 18948-44; Nacalai Tesque) and 0.2 M 1-ethyl-3-(3-dimethylaminopropyl) carbodiimide hydrochloride (EDAC; 346-03632; Wako) in 0.1 M MES buffer (pH 6.1) for 30 min at room temperature, washed with cold 60% methanol/PBS for 2 h at 4°C, and reacted overnight with 0.05 mg/mL Matrigel diluted in PBS at 4°C. After washing three times with cold PBS overnight at 4°C, the gels were placed on the bottom of PS dishes and exposed to UV light in a sterile hood for 30 min, during which the gels were tightly attached to the dish bottoms. Before plating the cells, gel-attached dishes were equilibrated in DMEM supplemented with 10% fetal bovine serum for at least 30 min at 37°C. Conventional PS dishes were coated with Matrigel according to manufacturer's instructions.

### Data and Code Availability

The data that support the findings of this study are available from the corresponding author upon request. The microarray data reported in this paper have been uploaded to the NCBI GEO under the accession number GSE125811.

## Author Contributions

S.K., T. Sadahiro, and M. Ieda designed the experiments. S.K., T. Sadahiro, R.F., H.T., H.Y., F.T., M. Isomi, H.K., Y.Y., Y.A., Y.M., T.A., and N.M. carried out the experiments. S.K., T. Sadahiro, I.H., T. Suzuki, and K.F. analyzed the data. S.K., T. Sadahiro, and M. Ieda wrote the paper.

## Conflicts of Interests

S.K. is the employee of Otsuka Pharmaceutical Co., Ltd. I.H. is the employee of Canon Inc. M. Ieda holds a patent related to this work: U.S. Patent 9,517,250 entitled “Methods for Generating Cardiomyocytes,” issued on October 19, 2012. Inventors: Deepak Srivastava and Masaki Ieda.

## Supplemental Information

Document S1. Supplemental Experimental Procedures, Figures S1–S5, and Tables S1–S3

Document S2. Article plus Supplemental Information
