# Supplementary material for: Soft Matrix Promotes Cardiac Reprogramming via Inhibition of YAP/TAZ and Suppression of Fibroblast Signatures
Source: Stem Cell Reports. 2020 Aug 27;15(3):612–28. doi: 10.1016/j.stemcr.2020.07.022 (PMC7486305; doi:10.1016/j.stemcr.2020.07.022)
Supplement: Document S1. Supplemental Experimental Procedures, Figures S1–S5, and Tables S1–S3 [file mmc1.pdf]

Stem Cell Reports, Volume *15*

Supplemental Information

Soft Matrix Promotes Cardiac Reprogramming via Inhibition of YAP/TAZ and Suppression of Fibroblast Signatures

Shota Kurotsu, Taketaro Sadahiro, Ryo Fujita, Hidenori Tani, Hiroyuki Yamakawa, Fumiya Tamura, Mari Isomi, Hidenori Kojima, Yu Yamada, Yuto Abe, Yoshiko Murakata, Tatsuya Akiyama, Naoto Muraoka, Ichiro Harada, Takeshi Suzuki, Keiichi Fukuda, and Masaki Ieda

Figure S1

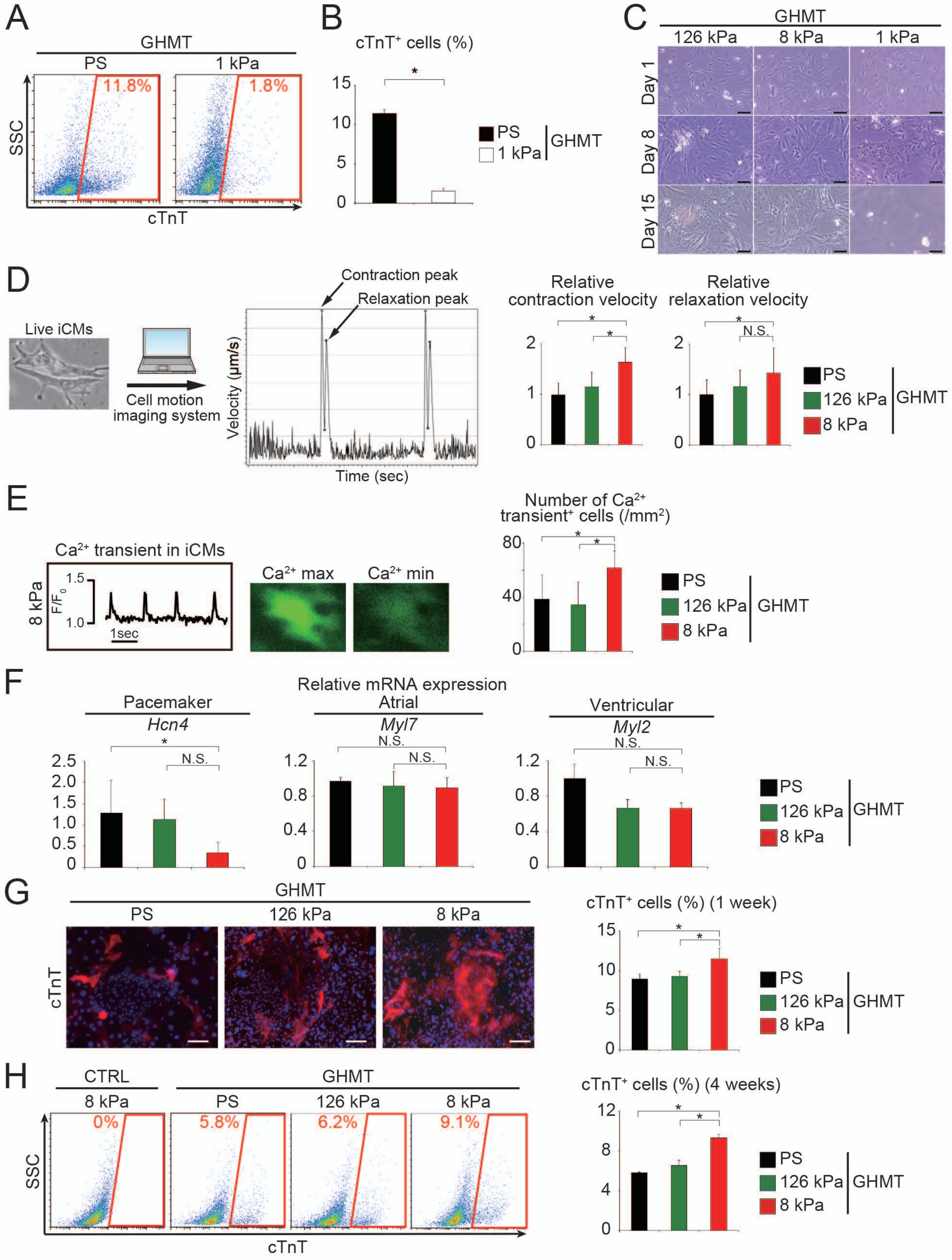

A
GHMT
PS
1 kPa
11.8%
1.8%
SSC
cTnT
B
cTnT+ cells (%)
15
10
5
0
*
PS
1 kPa
GHMT
C
GHMT
126 kPa
8 kPa
1 kPa
Day 1
Day 8
Day 15
D
Live iCMs
Cell motion imaging system
Contraction peak
Relaxation peak
Velocity (μm/s)
Time (sec)
Relative contraction velocity
Relative relaxation velocity
N.S.
PS
126 kPa
8 kPa
GHMT
E
Ca2+ transient in iCMs
8 kPa
F/F0
1.5
1.0
1sec
Ca2+ max
Ca2+ min
Number of Ca2+ transient+ cells (/mm2)
80
40
0
F
Relative mRNA expression
Pacemaker
Hcn4
Atrial
Myl7
Ventricular
Myl2
2.5
2.0
1.5
1.0
0.5
1.2
0.8
0.4
G
cTnT
cTnT+ cells (%) (1 week)
H
CTRL
0%
5.8%
6.2%
9.1%
cTnT+ cells (%) (4 weeks)
12
8
4

Figure S2

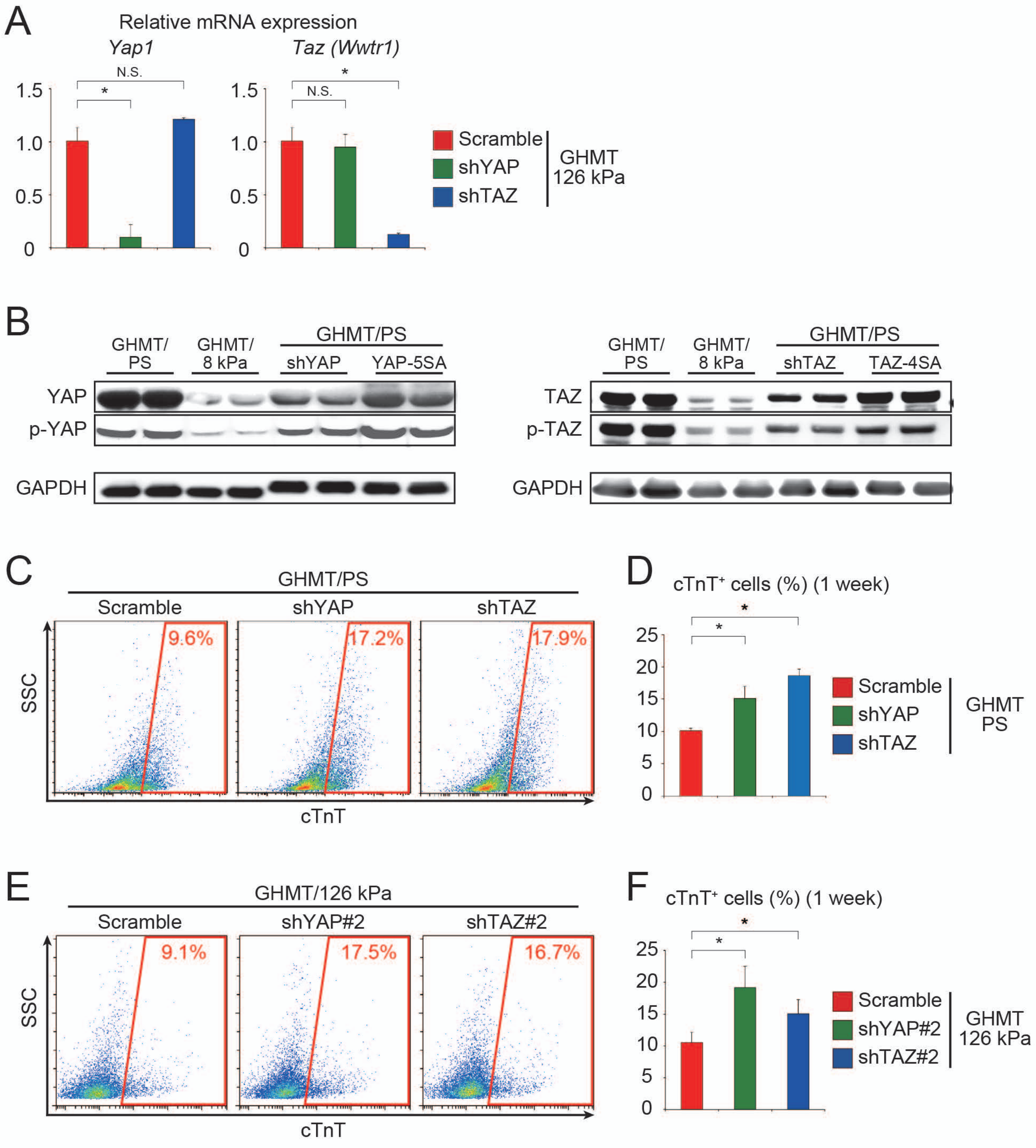
A
Relative mRNA expression
Yap1
Taz (Wwtr1)
N.S.
*
1.5
1.0
0.5
0
Scramble
shYAP
shTAZ
GHMT
126 kPa
B
GHMT/
PS
GHMT/
8 kPa
GHMT/PS
shYAP
YAP-5SA
YAP
p-YAP
GAPDH
shTAZ
TAZ-4SA
TAZ
p-TAZ
C
GHMT/PS
Scramble
shYAP
shTAZ
9.6%
17.2%
17.9%
SSC
cTnT
D
cTnT+ cells (%) (1 week)
25
20
15
10
5
0
GHMT
PS
E
GHMT/126 kPa
shYAP#2
shTAZ#2
9.1%
17.5%
16.7%
F
cTnT+ cells (%) (1 week)
shYAP#2
shTAZ#2

Figure S3

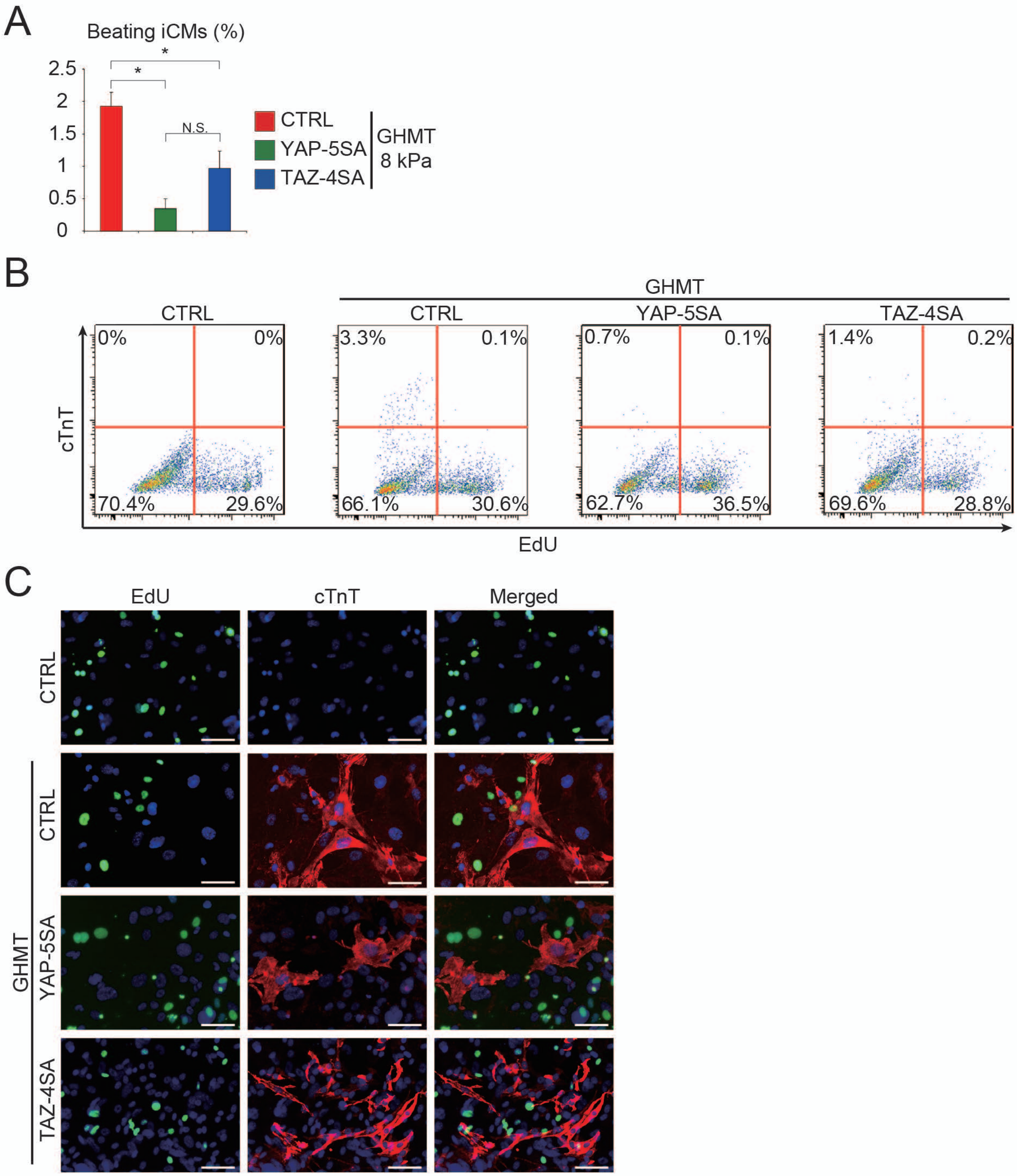
A
Beating iCMs (%)
2.5
2
1.5
1
0.5
0
*
*
N.S.
CTRL
YAP-5SA
TAZ-4SA
GHMT
8 kPa
B
GHMT
CTRL
CTRL
YAP-5SA
TAZ-4SA
cTnT
EdU
0%
0%
70.4%
29.6%
3.3%
0.1%
66.1%
30.6%
0.7%
0.1%
62.7%
36.5%
1.4%
0.2%
69.6%
28.8%
C
EdU
cTnT
Merged
CTRL
GHMT
CTRL
YAP-5SA
TAZ-4SA

Figure S4

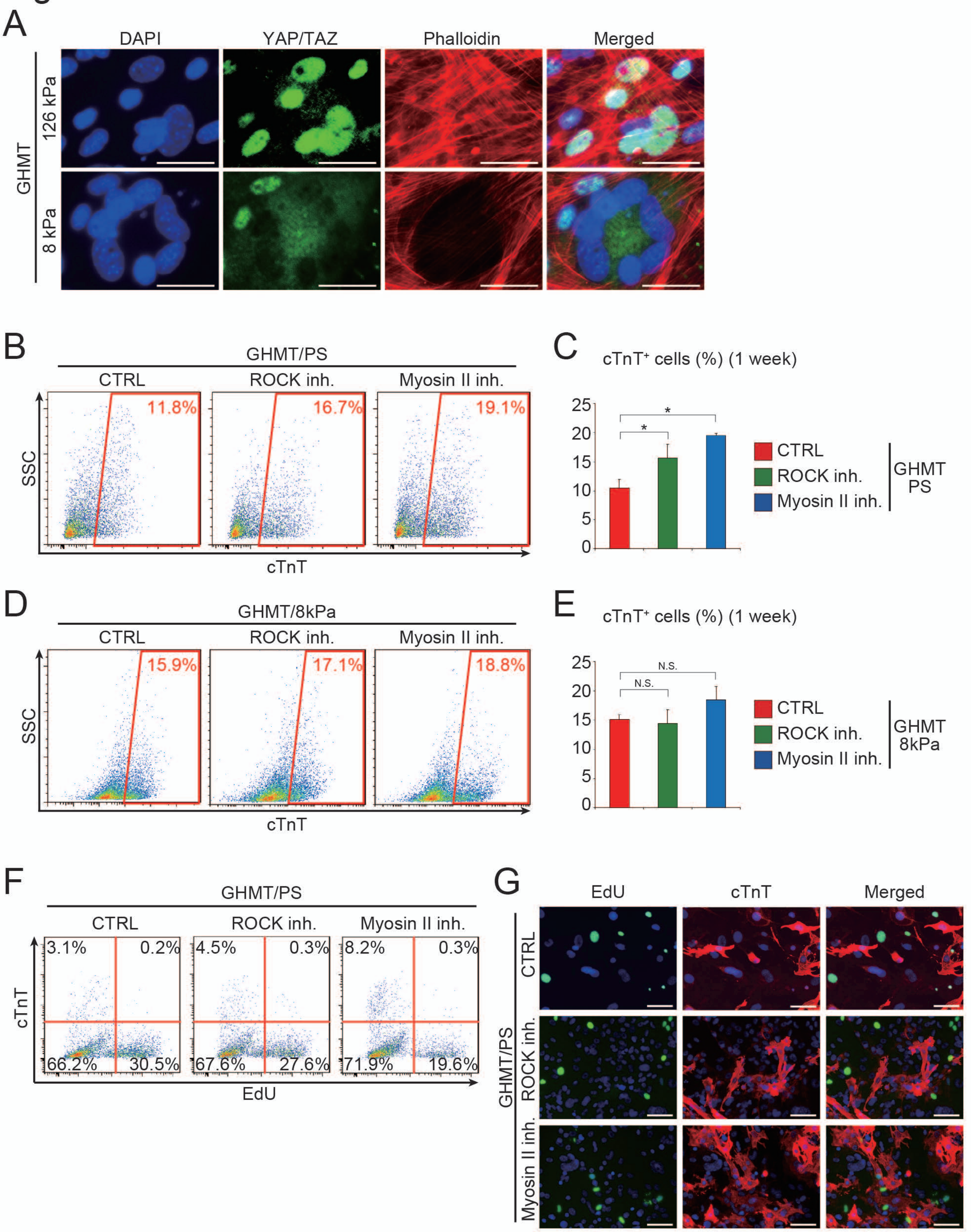
A
DAPI
YAP/TAZ
Phalloidin
Merged
GHMT
126 kPa
8 kPa
B
GHMT/PS
CTRL
ROCK inh.
Myosin II inh.
11.8%
16.7%
19.1%
SSC
cTnT
C
cTnT+ cells (%) (1 week)
25
20
15
10
5
0
*
*
CTRL
ROCK inh.
Myosin II inh.
GHMT
PS
D
GHMT/8kPa
CTRL
ROCK inh.
Myosin II inh.
15.9%
17.1%
18.8%
SSC
cTnT
E
cTnT+ cells (%) (1 week)
N.S.
N.S.
CTRL
ROCK inh.
Myosin II inh.
GHMT
8kPa
F
GHMT/PS
CTRL
ROCK inh.
Myosin II inh.
3.1%
0.2%
66.2%
30.5%
4.5%
0.3%
67.6%
27.6%
8.2%
0.3%
71.9%
19.6%
cTnT
EdU
G
EdU
cTnT
Merged
GHMT/PS
CTRL
ROCK inh.
Myosin II inh.

Figure S5

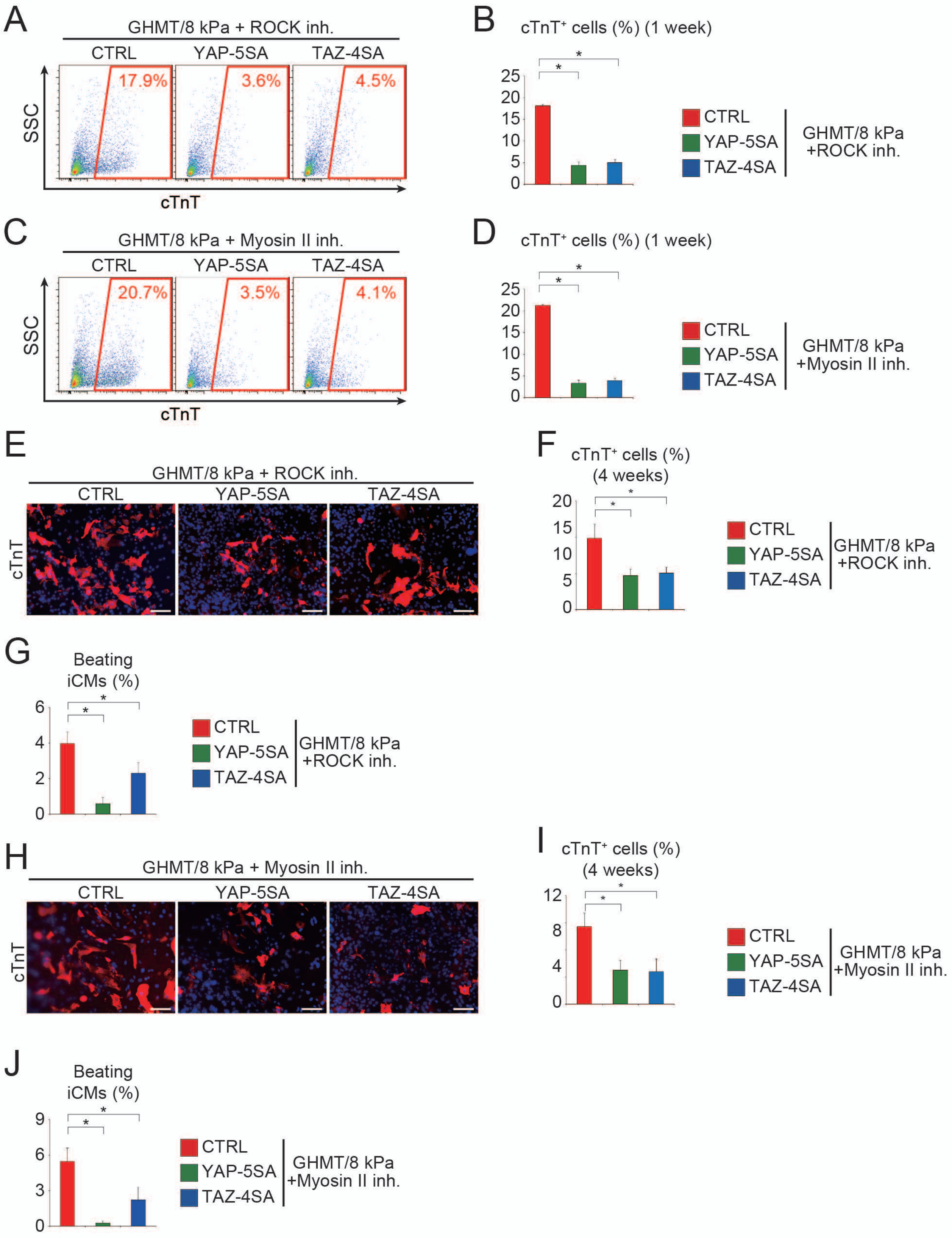

A
GHMT/8 kPa + ROCK inh.
CTRL
YAP-5SA
TAZ-4SA
17.9%
3.6%
4.5%
SSC
cTnT
B
cTnT+ cells (%) (1 week)
CTRL
YAP-5SA
TAZ-4SA
GHMT/8 kPa +ROCK inh.
C
GHMT/8 kPa + Myosin II inh.
20.7%
3.5%
4.1%
D
cTnT+ cells (%) (1 week)
GHMT/8 kPa +Myosin II inh.
E
GHMT/8 kPa + ROCK inh.
F
cTnT+ cells (%) (4 weeks)
G
Beating iCMs (%)
H
GHMT/8 kPa + Myosin II inh.
I
cTnT+ cells (%) (4 weeks)
J
Beating iCMs (%)

Supplemental Information

Supplemental Figure Legends

Figure S1. Soft matrix improves quality of cardiac reprogramming. Related to Figure 1.
(A, B) FACS analysis of cTnT expression in GHMT-transduced fibroblasts cultured on PS and 1 kPa substrates. **(B)** Quantitative data; n = 3 independent triplicate experiments.
(C) Time course of bright-field images of GHMT-transduced fibroblasts cultured on 1 kPa, 8 kPa, and 126 kPa hydrogels. Note that the cell number was decreased under 1kPa hydrogel culture after 15 days.
(D) High-resolution motion capture tracking system for the iCM contraction and relaxation analyses at 4 weeks after GHMT transduction on different substrates. Relative contraction and relaxation peak velocities of the iCMs are shown on the right (n = 3, independent triplicate experiments).
(E) Calcium transients from spontaneously contracting iCMs at 6 weeks after transduction. Fluo-4 intensity trace is shown on the left panel; representative images of maximum and minimum concentrations of Ca^{2+} signals are shown in the middle panels. Quantification of the number of Ca^{2+} oscillation^{+} cells after 6 weeks is depicted on the right; n = 3, independent triplicate experiments.
(F) Relative mRNA expression in the GHMT-transduced cells cultured on different substrates determined by qRT-PCR (n = 3, independent triplicate experiments).
(G) Immunocytochemistry of cTnT and DAPI after 1 week. Quantification of cTnT^{+} cells; n = 3, independent triplicate experiments.
(H) FACS analysis of cTnT expression after 4 weeks. Quantification of cTnT^{+} cells; n = 3, independent triplicate experiments.
All data are presented as mean ± SD. *P < 0.05 versus the corresponding control. NS, not significant; CTRL, control. Scale bars = 100 µm.

Figure S2. shYAP and shTAZ enhanced cardiac reprogramming. Related to Figure 3.
(A) Relative mRNA expression levels of *Yap1* and *Taz (Wwtr1)* were determined by qRT-PCR after 72 h (n = 4 independent triplicate experiments).
(B) Western blot analysis of YAP/p-YAP (left), TAZ/p-TAZ (right), and GAPDH. Fibroblasts were transduced with GHMT, cultured on PS, 8 kPa, and PS, and treated with shYAP, YAP-5SA, shTAZ, and TAZ-4SA. shYAP and shTAZ downregulated YAP and TAZ protein expression, respectively (n = 2, independent biological replicates).
(C, D) FACS analysis of cTnT expression after 1 week. GHMT-transduced fibroblasts were cultured on PS and then transfected with scrambled shRNA, shYAP, and shTAZ. **(D)** Quantification of cTnT^{+} cells; n = 3, independent triplicate experiments.
(E, F) FACS analysis of cTnT expression after 1 week. GHMT-transduced fibroblasts cultured on 126 kPa hydrogels were transduced with Scramble shRNA and YAP and TAZ-specific shRNA (shYAP#2 and shTAZ#2). (F) Quantitative data; n = 3 independent triplicate experiments.
All data are presented as the means ± SD. *P < 0.05 versus the relevant control. NS, not significant.

Figure S3. Activation of YAP and TAZ suppressed soft ECM-mediated cardiac reprogramming. Related to Figure 4.
(A) Quantitative data of the number of spontaneously beating cells after 4 weeks. GHMT-fibroblasts cultured on 8 kPa hydrogels were transduced with YAP-5SA and TAZ-4SA (n = 4 independent triplicate experiments).
(B) FACS analysis of EdU incorporation. GHMT-transduced MEFs were transduced with mock, YAP-5SA, and TAZ-4SA.
(C) Representative images of immunostaining showing cTnT and EdU incorporation into GHMT-transduced MEFs transduced with mock, YAP-5SA, and TAZ-4SA. The majority of cTnT-positive cells were negative for EdU.
All data are presented as the means ± SD. *P < 0.05 versus the relevant control. CTRL, control.

Figure S4. Soft ECM suppressed actomyosin organization and YAP/TAZ acitivity. Related to Figure 5.
(A) Immunocytochemistry of YAP/TAZ, phalloidin, and DAPI after 96 h of transduction. Fibroblasts cultured on 126 and 8 kPa hydrogels were transduced with GHMT. Phalloidin^{+} stress fibre formation and nuclear YAP/TAZ expression were reduced in cells cultured on 8 kPa hydrogels.
(B, C) FACS analysis of cTnT expression in GHMT-transduced MEFs cultured on PS dishes and treated with ROCK or myosin II inhibitor for 1 week. **(C)** Quantification of cTnT^{+} cells; (n = 3, independent triplicate experiments).
(D, E) FACS analysis of cTnT expression in GHMT-transduced MEFs cultured on 8 kPa hydrogels and treated with ROCK or myosin II inhibitor for 1 week. **(E)** Quantification of cTnT^{+} cells; (n = 3 independent triplicate

experiments).
(F, G) EdU incorporation assays for GHMT-transduced MEFs treated with ROCK or myosin II inhibitor. FACS **(F)** and immunostaining **(G)** demonstrated that inhibition of ROCK and myosin II did not enhance cell proliferation of iCMs. Representative images are shown.
All data are presented as the means ± SD. *P < 0.05 versus the relevant control. NS, not significant; CTRL, control; inh, inhibitor. Scale bars = 20 μm (A), 100 μm (G).

Figure S5. Activation of YAP/TAZ suppressed cardiac reprogramming induced by soft ECM and inhibition of ROCK or myosin II. Related to Figure 6.
(A, B) FACS analysis of cTnT expression after 1 week. GHMT-transduced cells cultured on 8 kPa hydrogels and treated with ROCK inhibitor were transduced with YAP-5SA and TAZ-4SA. Activation of YAP/TAZ suppressed ROCK inhibitor-mediated cardiac reprogramming on 8 kPa ECM. **(B)** Quantification of $cTnT^{+}$ cells; n = 3, independent triplicate experiments.
(C, D) FACS analysis of cTnT expression after 1 week. GHMT-transduced cells cultured on 8 kPa hydrogels and treated with myosin II inhibitor were transduced with YAP-5SA and TAZ-4SA. Activation of YAP/TAZ suppressed myosin II inhibitor-mediated cardiac reprogramming on 8 kPa ECM. **(D)** Quantification of $cTnT^{+}$ cells; n = 3, independent triplicate experiments.
(E, F) Immunocytochemistry of cTnT and DAPI after 4 weeks. YAP-5SA and TAZ-4SA suppressed cardiac reprogramming induced by soft ECM and ROCK inhibitor. **(F)** Quantification of $cTnT^{+}$ cells; n = 3, independent triplicate experiments.
(G) Quantification of the number of spontaneously beating cells after 4 weeks (n = 4, independent triplicate experiments). Activation of YAP/TAZ suppressed ROCK inhibitor-mediated cardiac reprogramming on 8 kPa hydrogels.
(H, I) Immunocytochemistry of cTnT and DAPI after 4 weeks. YAP-5SA and TAZ-4SA suppressed cardiac reprogramming induced with soft ECM and myosin II inhibitor. **(I)** Quantification of $cTnT^{+}$ cells; n = 3 independent triplicate experiments.
(J) Quantification of the number of spontaneously beating cells after 4 weeks (n = 4 independent triplicate experiments). Activation of YAP/TAZ suppressed myosin II inhibitor-mediated cardiac reprogramming on 8 kPa hydrogels.
All experiments were performed on 8 kPa hydrogels. All data are presented as the means ± SD. *P < 0.05 versus the relevant control. CTRL, control; inh, inhibitor. Scale bars = 100 μm.

Supplemental Table Legends

Table S1. shRNAs used in this study, related to Methods.

shRNA	Sense strand sequence
Scramble	GCGCGCTTTGTAGGATTCG
shYAP#1	TGAGAACAATGACAACCAATA
shYAP#2	CGGTTGAAACAACAGGAATTA
shTAZ#1	CAGCCGAATCTCGCAATGAAT
shTAZ#2	CCTGCATTTCTGTGGCAGATA

Table S2. TaqMan probes (Applied Biosystems) and qRT-PCR primer sequences for Universal Probe Library (Roche), related to Methods.
(A) TaqMan Gene Expression Assays (Applied Biosystems)

Gene name	Primer/Probe Set
Adrb1	Mm00431701_s1
Col1a1	Mm00801666_g1
Ctgf	Mm01192933_g1
Hcn4	Mm01176086_m1
Itgb2	Mm00434513_m1
Myh6	Mm00440359_m1
Myl2	Mm00440384_m1
Myl7	Mm00491655_m1
Pln	Mm04206542_m1
Serpine1	Mm00435858_m1
Taz (Wwtr1)	Mm01289583_m1
Thy1	Mm00493681_m1

Tnnt2	Mm01290256_m1
Yap	Mm01143263_m1

(B) Universal Probe Library System (Roche)

Gene name	Primer	Universal Probe Library
Gapdh	AGCTTGTCATCAACGGGAAG	#9
	TTTGATGTTAGTGGGGTCTCG	

Table S3. Concentration of acrylamide (AAm) and N,N'-methylenebisacrylamide (BIS) and the corresponding Young's modulus in polyacrylamide hydrogels, related to Methods.

AAm (%)	BIS (%)	ACA (mM)	Young's modulus (kPa)	Notation in text
7.5	0.64	70	126.2	126 kPa
7.5	0.32	90	67.2	67 kPa
7.5	0.16	115	30.1	30 kPa
7.5	0.08	150	13.8	14 kPa
7.5	0.04	190	7.5	8 kPa
7.5	0.02	245	4.0	4 kPa
7.5	0.01	306	2.0	2 kPa
7.5	0.005	392	≦1	1 kPa

Supplemental Movie Legend

Movie S1, related to Figure 1B.
Spontaneously beating GHMT-induced iCMs cultured on 8 kPa hydrogels for 4 weeks.

Supplemental Experimental Procedures

Generation of αMHC-GFP transgenic mice and fibroblast isolation

The Keio and Tsukuba University Ethics Committees for Animal Experiments approved all experiments in this study. Transgenic mice overexpressing GFP under the control of an α-MHC promoter were generated as described previously (Ieda et al., 2010). Mouse fibroblasts were isolated as described previously (Muraoka et al., 2014). In brief, mouse embryos isolated from 12.5-day pregnant mice were washed with phosphate-buffered saline (PBS), followed by careful removal of the head and visceral tissues. The remaining parts of the embryos were washed in fresh PBS, minced using a pair of scissors, transferred to a 0.125% trypsin/EDTA solution (25200-072; Gibco; Thermo Fisher Scientific, Waltham, MA), and incubated at 37 °C for 20 min. After trypsinization, an equal amount of fetal bovine serum (FBS) was added and pipetted several times to allow for tissue dissociation. After centrifugation of the tissue/medium mixture at 5000 × *g* for 5 min, the cells were collected and resuspended for culturing in DMEM (044-29765; Wako, Osaka, Japan) supplemented with 10% FBS at 37 °C in 5% CO_2 or stored in liquid nitrogen. Fibroblasts were plated at a density of 17,500–35,000 cells/cm^2 on PS dishes or hydrogels with various elasticities for virus transduction.

Measuring polyacrylamide gel elasticity

Gel rigidity was determined by the penetration method (Yip et al., 2013). In brief, Young's modulus (E) was obtained using the Hertz sphere model $\mathbf{h = bf^{2/3}}$, where $b = [9/(16\ ER^{1/2})]^{2/3}$ and a value of 1/2 for the Poisson ratio was assumed. The indentation profiles were obtained from fully hydrated 2–3 mm gel samples with a stainless-steel sphere (3 mm radius [R]). The force exerted on gels (f) was measured using a custom-designed electronic balance where gels were placed. Indentation of the sphere (h) was controlled using a micrometer. The Hertz model was then applied to fit the first linear section in the plot $f^{2/3}$ against indentation depth to ensure that the estimation was consistent with the linear approximation.

Retrovirus infection, lentivirus infection, Sendai virus vector infection, and cell culture

For constructing retroviral vectors, we used pMXs retroviral vectors for GFP, Gata4, Mef2c, Tbx5, and Hand2, which were described previously(Ieda et al., 2010). pQCX1H Myc-YAP (5SA) was obtained from Addgene (#33093; Watertown, MA) and pBABE FLAG-TAZ (4SA) was a gift from Dr. Guan (University of California San Diego)(Moroishi et al., 2016; Zhao et al., 2007). The vectors were transfected into Plat-E cells using Polyethylenimine "MAX" (24765-2; Polysciences Inc., Warrington, PA) according to the standard protocol. shRNA constructs were designed to target 21-base pair gene-specific regions in mouse YAP and TAZ. These oligonucleotides or scrambled oligonucleotides were cloned into CS-RfA-CG lentiviral vectors (RIKEN BioResource Center [BRC], Tsukaba, Japan) using the Gateway system (12538-120; Invitrogen, Carlsbad, CA).

To generate lentiviruses, we transfected the vectors into 293T cells with the packaging plasmids pMDL g/pRRE (RIKEN BRC) and pCMV-VSV-G-RSV-Rev (RIKEN BRC) using Lipofectamine 2000 (11668-019; Invitrogen). Retrovirus or lentivirus-containing supernatants were collected after 48 hours, filtered through 0.45-mm pore membranes, mixed with polybrene (TR-1003-G; Millipore, Burlington, MA) to a final concentration of 4 µg/mL, and transduced into fibroblasts. We routinely used suitable controls, pMX-GFP and CS-RfA-CG (containing CMV-GFP), to monitor transduction efficiency; we obtained efficiencies of 95%–100% in this study. The medium was replaced with DMEM/M199 (11150-059; Gibco) supplemented with 20% FBS after 24 h of infection, followed by replacement with FFV medium after 2 weeks, as described previously(Yamakawa et al., 2015). Generation and transduction of Sendai viral (SeV) vectors were performed as described previously (Miyamoto et al., 2018). Briefly, to generate F-deficient temperature-sensitive (TS) SeV vectors (SeV/TSΔF) expressing GMT, a polycistronic Gata4-Mef2c-Tbx5 was inserted into the cDNA of SeV/TSΔF vector (Ban et al., 2011; Li et al., 2000). Fibroblasts were transduced with the SeV vectors and cultured in DMEM/10% FBS at 35 °C in 5% CO_2 for 16 h. The medium was replaced with the FFV medium after 24 h of infection, and the cells were subsequently cultured at 37 °C in 5% CO_2. FFV medium contained StemPro-34 SF medium (10639-011; Gibco), GlutaMAX (10 µL/mL; 35050-061; Gibco), ascorbic acid (50 µg/mL; A-4544; Sigma-Aldrich, St. Louis, MO), recombinant human VEGF165 (5 ng/mL; 293-VE-050; R&D Systems, Minneapolis, MN), recombinant human FGF basic 146 aa (10 ng/mL; 233-FB-025; R&D Systems), and recombinant human FGF10 (50 ng/mL; 345-FG-025; R&D Systems). The medium was changed twice a week. Integrin signaling inhibitors (PF-00562271; S2672, 1 µM; Cayman, Ann Arbor, MI, RGDS peptide; 3498, 100 µM; R&D Systems), ROCK inhibitor (Y-27632; 250-00613, 30 µM; Wako) and Myosin II inhibitor (blebbistatin; 13013; 10 µM; Cayman) were added to DMEM/M199/20% FBS medium and FFV medium as indicated.

FACS analysis
Cells were fixed in 4% paraformaldehyde (PFA) for 15 min and stained with anti-cTnT antibody (MS-295-P1; Thermo Fisher Scientific) followed by incubation with secondary antibody conjugated to Alexa Fluor 647 (A21240; Invitrogen)(Ieda et al., 2010). The cells were resuspended in 5% FBS/PBS including saponin (0.5%; 47036-250G-F; Sigma-Aldrich) and analysed using a FACS instrument (CytoFLEX S; Beckman Coulter, Brea, CA).

Immunocytochemistry
Cells were fixed in 4% PFA for 15 min at room temperature, blocked with 5% Normal Goat Serum Blocking Solution (S-1000; Vector Laboratories, Burlingame, CA), and incubated with primary antibodies against α-actinin, cTnT, GFP (598; MBL, Woburn, MA), and YAP/TAZ (#8418; Cell Signaling Technology, Danvers, MA). The cells were then incubated with secondary antibodies conjugated to Alexa Fluor 488 (A11008; Invitrogen) or Alexa Fluor 546 (A11010, A11003; Invitrogen), followed by counterstaining with DAPI (D1306; Invitrogen). The percentage of GFP, α-actinin, and cTnT-immuno-positive cells was counted in 10–15 randomly selected fields per well in at least three independent experiments with 2000–4000 cells counted in total. The measurements and calculations were conducted in a blinded manner. The percentage of nuclear YAP/TAZ was also counted in 10–15 randomly selected fields per well in at least three independent experiments with 2000–4000 cells counted in total. All experiments were performed using an All-in-One fluorescence microscope (BZ-X810; Keyence, Osaka, Japan)(Yamakawa et al., 2015).

Western blot analysis
Lysates were prepared by homogenization of cells in RIPA buffer (08714-04; Nacalai Tesque) and then run on sodium dodecyl sulphate-polyacrylamide gels to separate proteins prior to immunoblot analyses. After transferring to PVDF membranes, immunodetection was performed using antibodies against GATA4 (sc-25310; Santa Cruz, Dallas, TX), MEF2C (ab197070; Abcam, Cambridge, UK), TBX5 (13178-1-AP; Proteintech, Rosemont, IL), YAP/TAZ (#8418; Cell Signaling Technology), phospho-YAP (Ser127; #13008; Cell Signaling Technology), phospho-TAZ (Ser89; #59971; Cell Signaling Technology), LATS1 (#3477; Cell Signaling Technology), phospho-LATS1 (Thr1079; #8654; Cell Signaling Technology), and GAPDH (#2118; Cell Signaling Technology) followed by treatment with horseradish peroxidase-conjugated anti-rabbit IgG secondary antibody (#7074S; Cell Signaling Technology). The antibody-bound proteins were visualised by Chemi-Lumi One L (#07880; Nacalai Tesque) and imaged using a CCD camera imaging system (ImageQuant LAS500; GE Healthcare, Chicago, IL)(Miyamoto et al., 2018). Signal intensity was measured by Image J software (National Institutes of Health [NIH], Bethesda, MD).

EdU labeling assay
To assess cell proliferation, 10 µM EdU was added to the culture medium 2 weeks after transduction and this concentration was maintained throughout the culture for additional 2 weeks. Cells were fixed with 4% PFA for 15 min, permeabilized, and incubated with anti-cTnT antibody. Next, the cells were incubated with secondary

antibodies conjugated to Alexa Fluor 546 (for immunocytochemistry) or 647 (for FACS), and EdU was detected using the Click-iT EdU Alexa Fluor 488 HCS Assay (C10350; Invitrogen) according to the manufacturer's instructions.

Counting of beating cells

For accurate analyses of the cell count, we used the All-in-One fluorescence(Miyamoto et al., 2018; Yamakawa et al., 2015). Cells were maintained at 37 °C and 5% CO_2 using the controlled chamber within the microscope. The number of spontaneously contracting cells in each field were counted for 25 regions with the 20× phase contrast lens; at least three independent experiments were performed. Individual beating cells were identified based on differences in beating frequency, cell-membrane boundary, and nuclei identified by phase-contrast microscopy. Cardiac reprogramming efficiency was determined as the number of beating cells per total nuclei counts at the time of the assay. Measurements and calculations were conducted in a blinded manner.

Ca^{2+} imaging

Ca^{2+} imaging was performed following standard protocols. Briefly, cells were labeled with Fluo-4 AM solution (F14201; Thermo Scientific, Waltham, MA) for 30 min at room temperature, washed, and incubated for additional 30 min, to allow de-esterification of the dye. Fluo-4-labeled cells were analyzed using an All-in-One fluorescence microscope at 37 °C. Ca^{2+} oscillation$^+$ cells were manually counted in 15 randomly selected fields per well, in three independent experiments. The measurements and quantification of the results were conducted in a blinded manner.

Quantitative RT-PCR

Total RNA was isolated from cells using the ReliaPrep RNA Cell Miniprep System (Z6012; Promega, Madison, WI) and then qRT-PCR was performed on a ViiA7 (Applied Biosystems, Foster City, CA) with TaqMan (Applied Biosystems) or Roche (Basel, Switzerland) probes. Primer and probe details are provided in Table S2. mRNA levels were normalised relative to *Gapdh*.

Gene microarray analyses

Microarray analyses were performed in duplicate, using independent biological samples. RNA was extracted from cells using the ReliaPrep RNA Cell Miniprep System. RNA quality was determined by the RNA Integrity Number (RIN) value using an RNA6000 assay (Agilent Technologies, Santa Clara, CA). Only specimens with RIN > 7.0 were used in this study. Gene expression levels were determined via microarrays (Clariom S Array, Mouse; Affymetrix, Santa Clara, CA) according to manufacturer's instructions. Prior to analysis, all data were normalised using a Single Space Transformation and Robust Multichip Analysis (SST-RMA) algorithm with Affymetrix Expression Console software version 1.4. Heatmaps and Venn diagrams were generated using GeneSpring GX 14.8 software (Agilent Technologies). GO analyses were performed using Database for Annotation, Visualization, and Integrated Discovery (DAVID; http://david.abcc.ncifcrf.gov/). Gene set enrichment analyses (GSEA) were performed using GSEA software (Broad Institute, Cambridge, MA).

Motion analyses of iCMs

To evaluate the functional parameters of iCMs, we used the SI8000 Cell Motion Imaging System (SONY, Tokyo, Japan), a high-resolution motion capture tracking system and a high speed video microscopy to detect and record cell motion. Spontaneous beating activity of iCMs was recorded and the motion of each detection point was converted into a motion vector. Quantitative analyses of the contraction velocity (CV) and the relaxation velocity (RV) of beating iCMs were also performed. The maximum CV and RV correspond to the contractile and diastolic function of iCMs, respectively (Ito et al., 2019; Kitani et al., 2019). Beating iCMs were recorded in 10 randomly selected fields per well. All movies were recorded with the resolutions of 2048 × 2048 pixels at 150 fps. Maximum CV and RV were assessed from the averaged contraction-relaxation waveforms during a 5 s recording.

Statistical analysis

Statistical parameters including the number of samples (n), descriptive statistics (mean and standard deviation), and significance are reported in the Figures and Figure legends. In general, at least n = 3 were used for each time point and experiment. Differences between groups were examined for statistical significance using the Student's *t*-test or one-way analysis of variance (ANOVA) followed by Dunnett's post-hoc test. Differences with P values < 0.05 were regarded as significant.

Supplemental References

Ban, H., Nishishita, N., Fusaki, N., Tabata, T., Saeki, K., Shikamura, M., Takada, N., Inoue, M., Hasegawa, M., Kawamata, S., et al. (2011). Efficient generation of transgene-free human induced pluripotent stem cells (iPSCs)

by temperature-sensitive Sendai virus vectors. Proceedings of the National Academy of Sciences of the United States of America 108, 14234-14239.
Ieda, M., Fu, J.D., Delgado-Olguin, P., Vedantham, V., Hayashi, Y., Bruneau, B.G., and Srivastava, D. (2010). Direct reprogramming of fibroblasts into functional cardiomyocytes by defined factors. Cell 142, 375-386.
Ito, M., Hara, H., Takeda, N., Naito, A.T., Nomura, S., Kondo, M., Hata, Y., Uchiyama, M., Morita, H., and Komuro, I. (2019). Characterization of a small molecule that promotes cell cycle activation of human induced pluripotent stem cell-derived cardiomyocytes. Journal of molecular and cellular cardiology 128, 90-95.
Kitani, T., Ong, S.G., Lam, C.K., Rhee, J.W., Zhang, J.Z., Oikonomopoulos, A., Ma, N., Tian, L., Lee, J., Telli, M.L., et al. (2019). Human-Induced Pluripotent Stem Cell Model of Trastuzumab-Induced Cardiac Dysfunction in Patients With Breast Cancer. Circulation 139, 2451-2465.
Li, H.O., Zhu, Y.F., Asakawa, M., Kuma, H., Hirata, T., Ueda, Y., Lee, Y.S., Fukumura, M., Iida, A., Kato, A., et al. (2000). A cytoplasmic RNA vector derived from nontransmissible Sendai virus with efficient gene transfer and expression. Journal of virology 74, 6564-6569.
Miyamoto, K., Akiyama, M., Tamura, F., Isomi, M., Yamakawa, H., Sadahiro, T., Muraoka, N., Kojima, H., Haginiwa, S., Kurotsu, S., et al. (2018). Direct In Vivo Reprogramming with Sendai Virus Vectors Improves Cardiac Function after Myocardial Infarction. Cell Stem Cell 22, 91-103 e105.
Moroishi, T., Hayashi, T., Pan, W.W., Fujita, Y., Holt, M.V., Qin, J., Carson, D.A., and Guan, K.L. (2016). The Hippo Pathway Kinases LATS1/2 Suppress Cancer Immunity. Cell 167, 1525-1539 e1517.
Yamakawa, H., Muraoka, N., Miyamoto, K., Sadahiro, T., Isomi, M., Haginiwa, S., Kojima, H., Umei, T., Akiyama, M., Kuishi, Y., et al. (2015). Fibroblast Growth Factors and Vascular Endothelial Growth Factor Promote Cardiac Reprogramming under Defined Conditions. Stem Cell Reports 5, 1128-1142.
Yip, A.K., Iwasaki, K., Ursekar, C., Machiyama, H., Saxena, M., Chen, H., Harada, I., Chiam, K.H., and Sawada, Y. (2013). Cellular response to substrate rigidity is governed by either stress or strain. Biophys J 104, 19-29.
Zhao, B., Wei, X., Li, W., Udan, R.S., Yang, Q., Kim, J., Xie, J., Ikenoue, T., Yu, J., Li, L., et al. (2007). Inactivation of YAP oncoprotein by the Hippo pathway is involved in cell contact inhibition and tissue growth control. Genes Dev 21, 2747-2761.
