# Supplementary material for: Soft Matrix Promotes Cardiac Reprogramming via Inhibition of YAP/TAZ and Suppression of Fibroblast Signatures
Source: Stem Cell Reports. 2020 Aug 27;15(3):612–28. doi: 10.1016/j.stemcr.2020.07.022 (PMC7486305; doi:10.1016/j.stemcr.2020.07.022)
Supplement: Document S2. Article plus Supplemental Information [file mmc3.pdf]

Stem Cell Reports
Article

OPEN ACCESS

Soft Matrix Promotes Cardiac Reprogramming via Inhibition of YAP/TAZ and Suppression of Fibroblast Signatures

Shota Kurotsu,[1,7,8] Taketaro Sadahiro,[2,8] Ryo Fujita,[2,3] Hidenori Tani,[1] Hiroyuki Yamakawa,[1] Fumiya Tamura,[1] Mari Isomi,[2] Hidenori Kojima,[1] Yu Yamada,[2] Yuto Abe,[2] Yoshiko Murakata,[2] Tatsuya Akiyama,[4] Naoto Muraoka,[1] Ichiro Harada,[5] Takeshi Suzuki,[6] Keiichi Fukuda,[1] and Masaki Ieda[2,*]

[1]Department of Cardiology, Keio University School of Medicine, 35 Shinanomachi, Shinjuku-ku, Tokyo 160-8582, Japan
[2]Department of Cardiology, Faculty of Medicine, University of Tsukuba, 1-1-1 Tennoudai, Tsukuba City, Ibaraki 305-8575, Japan
[3]Division of Regenerative Medicine, Transborder Medical Research Center, University of Tsukuba, 1-1-1 Tennoudai, Tsukuba City, Ibaraki 305-8575, Japan
[4]Department of Respiratory Medicine, Faculty of Medicine, University of Tsukuba, 1-1-1 Tennoudai, Tsukuba City, Ibaraki 305-8575, Japan
[5]Medical Products Technology Development Center, R&D Headquarters, Canon Inc., 3-30-2 Shimomaruko, Ohta-ku, Tokyo 146-8501, Japan
[6]Division of Basic Biological Sciences, Faculty of Pharmacy, Keio University, 1-5-30 Shibakoen, Minato-ku, Tokyo 105-8512, Japan
[7]Present address: Otsuka Pharmaceutical Co., Ltd., Frontier Sciences Unit, Department of Medical Innovations, New Drug Research Division, 463-10 Kagasuno, Kawauchi-cho, Tokushima 771-0192, Japan
[8]Co-first author
*Correspondence: mieda@md.tsukuba.ac.jp
https://doi.org/10.1016/j.stemcr.2020.07.022

SUMMARY

Direct cardiac reprogramming holds great potential for regenerative medicine. However, it remains inefficient, and induced cardiomyocytes (iCMs) generated *in vitro* are less mature than those *in vivo*, suggesting that undefined extrinsic factors may regulate cardiac reprogramming. Previous *in vitro* studies mainly used hard polystyrene dishes, yet the effect of substrate rigidity on cardiac reprogramming remains unclear. Thus, we developed a Matrigel-based hydrogel culture system to determine the roles of matrix stiffness and mechanotransduction in cardiac reprogramming. We found that soft matrix comparable with native myocardium promoted the efficiency and quality of cardiac reprogramming. Mechanistically, soft matrix enhanced cardiac reprogramming via inhibition of integrin, Rho/ROCK, actomyosin, and YAP/TAZ signaling and suppression of fibroblast programs, which were activated on rigid substrates. Soft substrate further enhanced cardiac reprogramming with Sendai virus vectors via YAP/TAZ suppression, increasing the reprogramming efficiency up to ~15%. Thus, mechanotransduction could provide new targets for improving cardiac reprogramming.

INTRODUCTION

Direct reprogramming generates the desired cell types from fibroblasts without passing through a stem cell state by overexpressing tissue-specific transcription factors (Sadahiro et al., 2015). This new technology has wide applications in disease modeling, drug discovery, and regenerative medicine (Isomi et al., 2019; Sadahiro et al., 2018; Srivastava and DeWitt, 2016). We and others reported the generation of induced cardiomyocytes (iCMs) from fibroblasts using cardiac transcription factors, including GATA4, MEF2C, and TBX5 (GMT) or GMT plus HAND2 (GHMT) (Ieda et al., 2010; Song et al., 2012). *In vivo* cardiac reprogramming by gene transfer of GMT or GHMT into mouse hearts reprogrammed resident cardiac fibroblasts (CFs) into iCMs, improved cardiac function, and reduced fibrosis after myocardial infarction (MI) (Inagawa et al., 2012; Miyamoto et al., 2018; Qian et al., 2012; Song et al., 2012). However, the cardiac reprogramming process remains inefficient and iCMs generated *in vitro* are immature, suggesting that undefined extrinsic factors may regulate the efficiency and quality of cardiac reprogramming (Qian et al., 2012; Song et al., 2012; Srivastava and DeWitt, 2016; Yamakawa et al., 2015; Zhao et al., 2015). Previous cardiac reprogramming studies have mainly used conventional rigid polystyrene dishes (PS; ~GPa) that exhibit distinct physical properties from native myocardium (10–20 kPa) (Berry et al., 2006; Engler et al., 2006); thus, the effects of substrate stiffness and mechanotransduction on cardiac reprogramming remain elusive.

Cells sense underlying matrix rigidity and propagate biochemical and biophysical signals along the cytoskeleton to the nucleus, altering gene expression and cellular function (Crowder et al., 2016; Dupont, 2016; Lyon et al., 2015). Previous studies demonstrated that stiffness of the extracellular matrix (ECM) changes cell adhesion, proliferation, and differentiation (Crowder et al., 2016; Engler et al., 2006, 2008; Lyon et al., 2015; Yalahom-Ronen et al., 2015). Engler et al., 2006 demonstrated that mesenchymal stem cells (MSCs) cultured on polyacrylamide gels differentiated into specific cell types that corresponded to the stiffness of their incorporated tissues; soft matrices specified the MSCs to the adipogenic lineage, while stiff matrices differentiated them toward the osteogenic lineage. Further mechanistic studies revealed that two highly related transcriptional coactivators, Yes-associated protein (YAP) and transcriptional coactivator with PDZ-binding domain (TAZ), were responsible for sensing ECM rigidity and acted as key regulators for mechanotransduction (Dupont, 2016; Dupont et al., 2011). It has been reported that

This is an open access article under the CC BY license (http://creativecommons.org/licenses/by/4.0/).

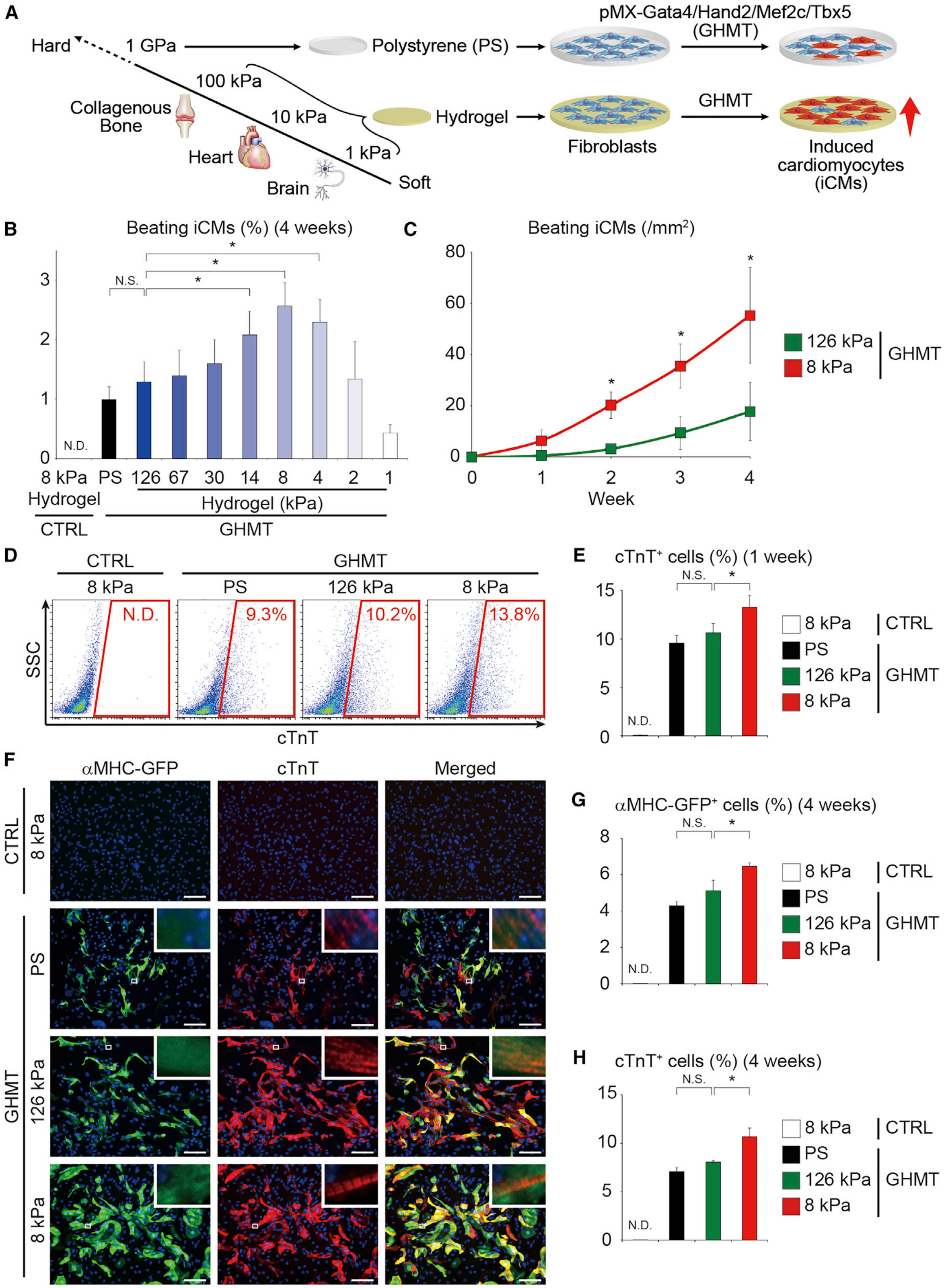

(legend on next page)

YAP activation promotes induced pluripotent stem cell (iPSC) reprogramming (Lian et al., 2010); however, the roles of YAP/TAZ in direct cardiac reprogramming remain unknown. Given that the myocardium becomes stiffer than healthy myocardium after MI via accumulation of ECM and fibrosis, understanding the effects of matrix stiffness and mechanotransduction in cardiac reprogramming would be clinically relevant (Berry et al., 2006; Engler et al., 2008). Therefore, in this study, we developed a Matrigel-based hydrogel culture system to investigate the mechanobiology in cardiac reprogramming and the signaling pathway involved.

RESULTS

Soft ECM Comparable with the Myocardium Promotes Cardiac Reprogramming

Given that tissue elasticity of the myocardium (10–20 kPa) is much softer than that of PS dishes (~GPa), we studied the effect of matrix stiffness on cardiac reprogramming (Berry et al., 2006; Engler et al., 2006). We plated fibroblasts from alpha myosin heavy chain (αMHC)-GFP transgenic mice, in which only cardiomyocytes express GFP (Ieda et al., 2010), on Matrigel-coated PS dishes or Matrigel-based hydrogels with different elasticities ranging from 1 kPa (similar to brain) to 126 kPa (similar to collagenous bone). The following day, we transduced the fibroblasts with the retroviral GHMT (pMX-GHMT) to induce cardiac reprogramming (Figure 1A). To identify the effect of ECM stiffness on cardiac reprogramming, we analyzed the number of spontaneously beating iCMs after 4 weeks. We found that soft substrates (4–14 kPa) resulted in a significantly higher number of beating iCMs with a peak at 8 kPa (comparable with the stiffness of native myocardium; Figure 1B, Video S1). Cardiac reprogramming was not induced without GHMT transduction on 8 kPa hydrogels. Consistent with a previous report (Herum et al., 2017), softer substrates (1–2 kPa) altered cell morphology into rounder and smaller shapes, leading to cell detachment and death (Figures 1B and S1A–S1C). Sequential analyses revealed that iCMs cultured on 8 kPa hydrogels started to contract earlier just after 1 week and that the number of beating iCMs increased by ~3-fold compared with the number of cells on 126 kPa hydrogels after 4 weeks (Figure 1C). Next, we evaluated contraction/relaxation velocities of beating iCMs using the new high-speed video microscopy and motion vector analysis to determine detailed functional properties of iCMs. The contraction/relaxation velocities of iCMs on 8 kPa hydrogels were higher than the velocities on rigid substrates, demonstrating functional maturation of iCMs on soft substrates (Figure S1D). The number of Ca^{2+} transient$^+$ cells on 8 kPa hydrogel also increased by ~2-fold relative to the cells cultured on rigid substrates after 4 weeks (Figure S1E). It was previously reported that all three subtypes of iCMs, such as pacemaker-, atrial-, and ventricular-like cardiomyocytes, were generated by direct cardiac reprogramming (Nam et al., 2014). Therefore, it is possible that soft matrix may induce the pacemaker cells to increase the number of beating cells. qRT-PCR analysis revealed that the expression of *Hcn4*, a marker for the pacemaker cells, was suppressed during culture on 8 kPa hydrogels, suggesting that soft matrix did not increase the induction of pacemaker cells (Figure S1F).

Fluorescence-activated cell sorting (FACS) analyses demonstrated that 8 kPa hydrogels induced more cardiac troponin T (cTnT)$^+$ cells than did PS or 126 kPa substrates after 1 and 4 weeks (Figures 1D, 1E, and S1H). Immunocytochemistry also revealed that, after 1 week, the number of cTnT$^+$ cells increased on 8 kPa substrates relative to that on PS or 126 kPa hydrogels; however, only a few cells exhibited sarcomeric structures at this early stage, consistent with the results of previous studies (Figure S1G) (Ieda et al., 2010; Song et al., 2012). The iCMs showed well-defined sarcomeric structures, and, after 4 weeks, more αMHC-GFP$^+$ and cTnT$^+$ cells were induced on 8 kPa hydrogels than on

Figure 1. Soft Matrices Promote Cardiac Reprogramming

(A) Solid tissues exhibit a range of stiffness, as measured by the elastic modulus. Conventional PS dishes and dishes with Matrigel-based hydrogels of different elasticities (1–126 kPa) were used for cardiac reprogramming. αMHC-GFP mouse fibroblasts were transduced with pMX-GHMT to generate induced cardiomyocytes (iCMs).

(B) Fibroblasts were seeded on PS and hydrogels of various elasticities and transduced with GHMT. The effect of substrate elasticity on cardiac reprogramming was analyzed by counting spontaneously beating cells after 4 weeks; n = 3 independent triplicate experiments.

(C) Time course of cardiac reprogramming on 126 kPa and 8 kPa hydrogel culture; n = 3 independent triplicate experiments.

(D and E) FACS analysis of cTnT expression after 1 week. Fibroblasts were transduced with GHMT and cultured on PS or 126 kPa and 8 kPa substrates. (E) Quantitative data; n = 3 independent triplicate experiments.

(F–H) Immunocytochemistry of αMHC-GFP, cTnT, and DAPI after 4 weeks. Cells were treated as described in (D). High-magnification views, as insets, show sarcomeric organization.

(G and H) Quantitative data; n = 3 independent triplicate experiments.

All data are presented as the means ± SD. *p < 0.05 versus the relevant control. NS, not significant; ND, not detected; CTRL, control. Scale bars represent 100 μm.

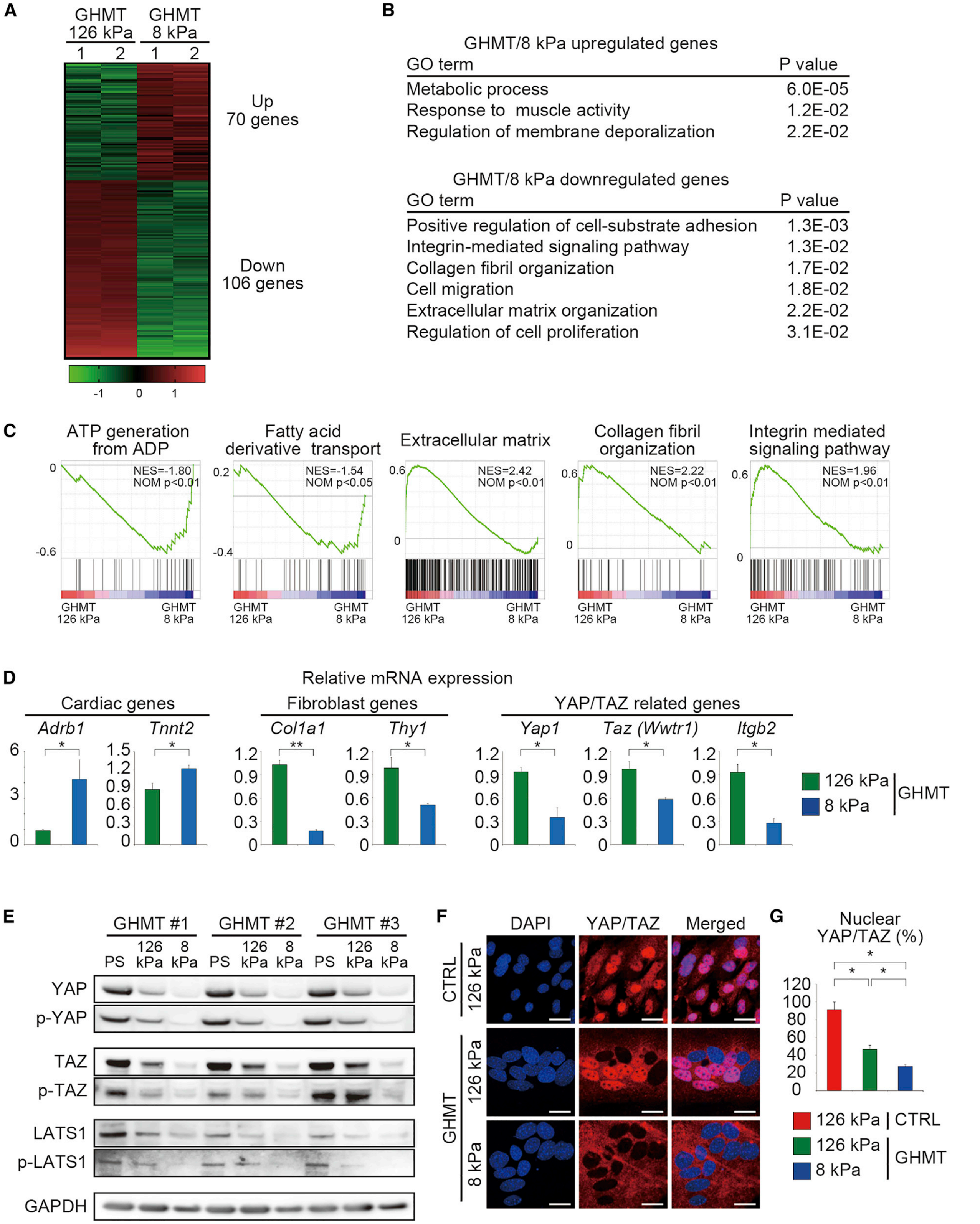

(legend on next page)

PS dishes or 126 kPa hydrogels (Figures 1F–1H). The relative increase in cTnT+ cells was ~1.5-fold, which was less than the increase in beating iCM generation, suggesting that soft substrates promote the degree of iCM maturation. These results demonstrated that soft matrix similar to the myocardial stiffness improved the efficiency and quality of cardiac reprogramming compared with the ECM stiffness of non-cardiac tissue or conventional PS dishes.

Soft ECM Induces Cardiac Programs and Suppresses YAP/TAZ Signaling

Next, to investigate the underlying mechanisms resulting in significant differences in cardiac reprogramming on 8 kPa and 126 kPa hydrogels, we performed microarray analyses to identify differentially expressed genes between the two groups. Differential gene expression analyses revealed that 70 genes were upregulated in GHMT-transduced fibroblasts on 8 kPa hydrogels, while 106 genes were downregulated at least 2-fold compared with those in the 126 kPa group (Figure 2A). Gene ontology (GO) analyses demonstrated that upregulated genes in cells grown on 8 kPa hydrogels were enriched for GO terms associated with cardiac function, while the downregulated genes were enriched for GO terms associated with fibroblast signatures (such as cell substrate adhesion, migration, and proliferation), ECM organization, and integrin-mediated signaling pathway (Figure 2B). Gene set enrichment analysis (GSEA) also demonstrated that genes related to the ATP generation and fatty acid transport were upregulated in GHMT-transduced fibroblasts cultured on 8 kPa hydrogels, whereas fibroblast-related genes were strongly downregulated in cells cultured on 8 kPa compared with those on 126 kPa hydrogels (Figure 2C). qRT-PCR results confirmed that cardiac genes, such as β1-adrenergic receptor (*Adrb1*) and sarcomeric structure (*Tnnt2*), were upregulated, whereas fibroblast genes (*Col1a1*, *Thy1*) were significantly downregulated in the 8 kPa group (Figure 2D). These results suggested that soft matrices induced cardiac gene programs concomitant with repression of fibroblast signatures.

YAP and TAZ are transcriptional coactivators that are imported into the nuclei when cells are cultured on rigid substrates, whereas they are exported to the cytoplasm, phosphorylated, and degraded via the proteasome system on elastic substrates (Dupont, 2016; Dupont et al., 2011; Ohgushi et al., 2015). Western blotting revealed that protein levels of total YAP and TAZ (nuclear and cytoplasmic localization) and phosphorylated YAP and TAZ (cytoplasmic localization) were lower in the GHMT-transduced cells cultured on soft ECM than on rigid substrates (Figure 2E). Consistent with these findings, qRT-PCR showed that gene expression levels of *Yap1*, *Taz (Wwtr1)*, and the YAP/TAZ target gene *Itgb2* were significantly lower in the GHMT-transduced cells cultured on 8 kPa than on 126 kPa hydrogels (Figure 2D). The canonical Hippo pathway, activated by contact inhibition, suppresses YAP and TAZ through the phosphorylation of large tumor suppressor homolog 1 (LATS1) (Zhao et al., 2007). Therefore, if the canonical Hippo signaling was responsible for YAP/TAZ suppression on 8 kPa substrate, LATS1 would be phosphorylated. However, western blotting demonstrated that the levels of both total and phosphorylated LATS1 decreased on 8 kPa hydrogel, suggesting that the canonical Hippo signaling might not be the primary regulator of YAP/TAZ in this particular model (Figure 2E). IHC revealed that most fibroblasts expressed YAP/TAZ in the nuclei on 126 kPa substrates under basal conditions (Figure 2F). After 3 days on 126 kPa substrate and with GHMT transduction, nuclear YAP/TAZ expression was reduced by ~50%, which was further reduced on 8 kPa culture in the presence of GHMT (Figures 2F and 2G). These results suggest that elastic matrices similar to the myocardium promote cardiac reprogramming concomitant with suppression of fibroblast signatures and YAP/TAZ signaling.

Inhibition of YAP/TAZ Promotes Cardiac Reprogramming and Suppresses Fibroblast Signatures

Our results showed that YAP/TAZ signaling was inhibited in soft ECM-mediated cardiac reprogramming; however,

Figure 2. Soft Matrix Suppresses Fibroblast Signature and YAP/TAZ during Cardiac Reprogramming

(A) Heatmap of microarray data illustrating the differentially expressed genes between the GHMT-transduced fibroblasts cultured on 126 kPa (GHMT/126 kPa) and 8 kPa (GHMT/8 kPa) hydrogels for 2 weeks; n = 2 independent biological replicates.

(B) GO term analysis of the upregulated and downregulated genes in GHMT/8 kPa compared with those of GHMT/126 kPa. Cardiac-related genes were upregulated, whereas fibroblast-related genes were downregulated in GHMT/8 kPa.

(C) GSEA for GHMT/8 kPa and GHMT/126 kPa.

(D) qRT-PCR analysis to determine relative mRNA expression of cardiomyocyte-, fibroblast-, and YAP/TAZ-related genes in GHMT/8 kPa-cultured cells compared with the GHMT/126 kPa-cultured cells; n = 3 independent triplicate experiments.

(E) Western blotting of total YAP, TAZ, LATS1, and phosphorylated YAP (pYAP), TAZ (pTAZ), and LATS1 (pLATS1) protein levels in GHMT-transduced fibroblasts on PS dishes or Matrigel-based hydrogels with different elasticities; n = 3 independent biological replicates.

(F and G) Immunocytochemistry of YAP/TAZ and DAPI after 72 h. (G) Quantitative data (n = 3 independent triplicate experiments). Nuclear YAP/TAZ expression was reduced in the presence of GHMT and under 8 kPa culture conditions.

All data are presented as the means ± SD. *$p < 0.05$; **$p < 0.01$ versus the relevant control. Scale bars represent 20 μm.

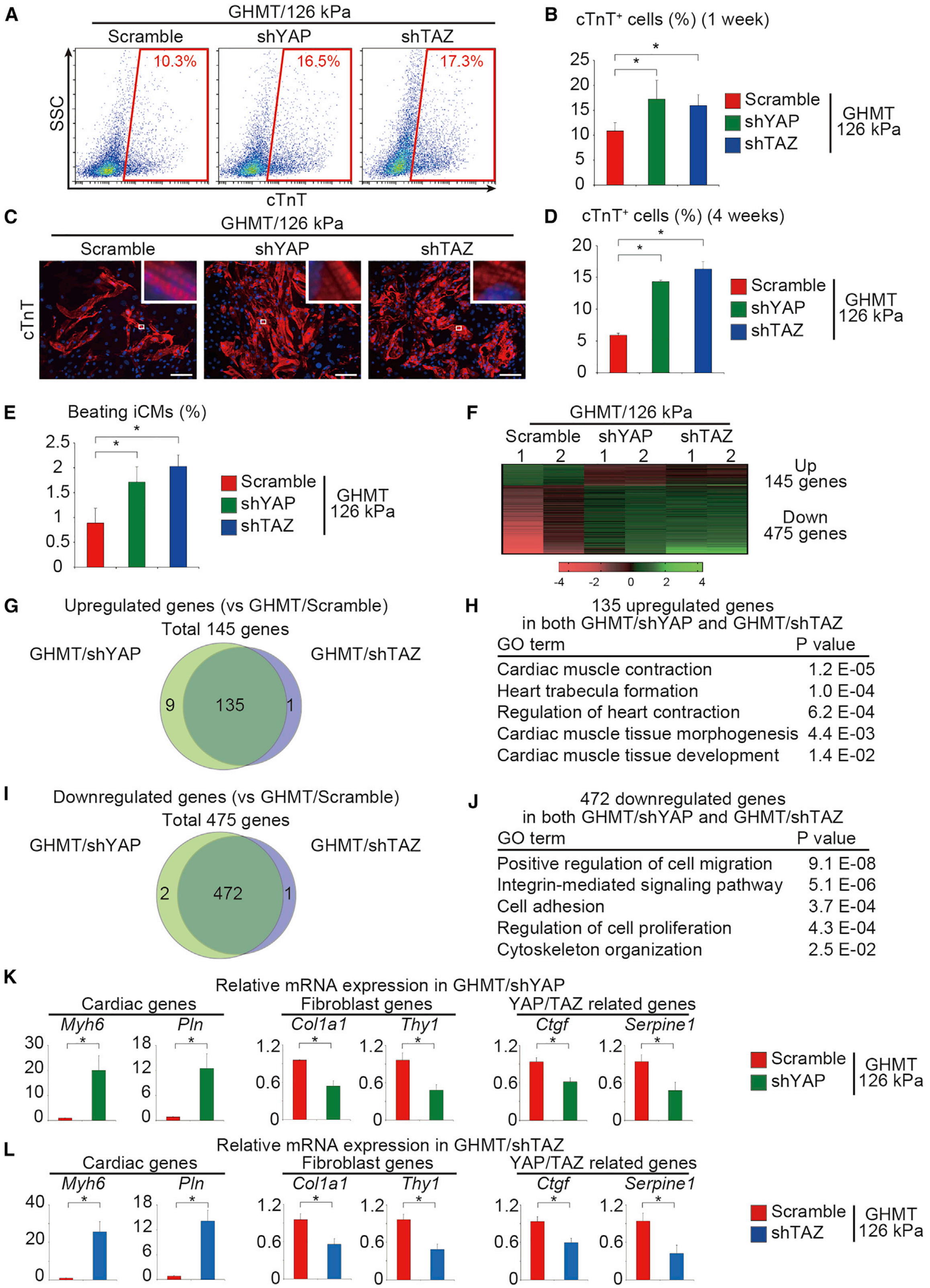

(legend on next page)

it is unknown whether this suppression improves iCM reprogramming. To test this, we suppressed YAP and TAZ expression with specific short hairpin RNAs (shRNAs) (shYAP and shTAZ) in GHMT-transduced fibroblasts on 126 kPa hydrogels (Table S1). Downregulation of YAP and TAZ expression by shYAP and shTAZ, respectively, was confirmed by qRT-PCR and western blotting (Figures S2A and S2B). FACS analyses revealed that induction of $cTnT^+$ cells significantly increased after treatment with shYAP or shTAZ more than with scramble shRNA on 126 kPa and PS dishes after 1 week (Figures 3A, 3B, S2C, and S2D). Knockdown of both YAP and TAZ did not further increase cardiac reprogramming (data not shown). Other shRNAs (shYAP#2 or shTAZ#2) also increased cardiac reprogramming, demonstrating the specific and reproducible effects of shYAP and shTAZ on cardiac reprogramming (Figures S2E and S2F, and Table S1). Immunocytochemistry of cTnT also demonstrated that YAP and TAZ knockdown increased the number of $cTnT^+$ cells with well-defined sarcomeric structures after 4 weeks (Figures 3C and 3D). Moreover, the number of beating iCMs also increased after shYAP and shTAZ treatment (Figure 3E).

We next performed microarray analyses to determine the molecular mechanism of shYAP and shTAZ-mediated cardiac reprogramming on 126 kPa hydrogels (Figure 3F). Compared with scramble shRNA, differential gene expression analyses revealed that, after either shYAP or shTAZ treatment, 145 genes were upregulated and 475 genes were downregulated with significant overlaps between the two shRNA groups, suggesting that shYAP and shTAZ largely target the same pathways (Figures 3F–3J). GO analyses showed that upregulated genes in the shYAP and shTAZ treatments were enriched for GO terms associated with cardiac function and development, while downregulated genes were enriched for GO terms associated with fibroblast signatures (cell migration, adhesion, and proliferation; Figures 3H and 3J). qRT-PCR analysis confirmed that inhibition of YAP and TAZ expression upregulated a panel of cardiac genes and significantly downregulated fibroblast and YAP/TAZ target genes, such as *Ctgf* and *Serpine1* (Figures 3K and 3L) (Dupont et al., 2011). Thus, inhibition of YAP/TAZ promoted cardiac reprogramming concomitant with suppression of fibroblast signatures, recapitulating the effect of soft substrate-mediated cardiac reprogramming.

Activation of YAP/TAZ Suppresses Soft Substrate-Mediated Cardiac Reprogramming

To determine whether soft substrates promote cardiac reprogramming through inhibition of YAP/TAZ, we tested whether activation of YAP/TAZ could counteract the effect of soft ECM-mediated cardiac reprogramming. We overexpressed YAP-5SA and TAZ-4SA, which are constitutive active mutants of YAP and TAZ, in GHMT-transduced fibroblasts plated on soft substrates (8 kPa) (Dupont et al., 2011; Moroishi et al., 2016; Zhao et al., 2007). Western blot analyses confirmed that total YAP and TAZ protein expression was significantly upregulated in the cells transduced with YAP-5SA and TAZ-4SA, respectively, whereas phosphorylated YAP and TAZ expression was unaltered, indicating activation of YAP/TAZ signaling in nuclei with these mutants (Figures 4A, 4B, and S2B). FACS analyses demonstrated that activation of YAP/TAZ strongly decreased the induction of $cTnT^+$ cells on 8 kPa hydrogels after 1 week (Figures 4C and 4D). Immunocytochemistry of αMHC-GFP and cTnT also demonstrated that overexpression of YAP-5SA and TAZ-4SA suppressed the induction of αMHC-GFP^+ and $cTnT^+$ cells after 4 weeks (Figures 4E–4G).

Figure 3. Inhibition of YAP/TAZ Enhances Cardiac Reprogramming and Suppresses Fibroblast Signature

(A and B) FACS analysis of cTnT expression after 1 week. GHMT-transduced fibroblasts cultured on 126 kPa hydrogels were transduced with Scramble shRNA and YAP and TAZ-specific shRNA (shYAP and shTAZ). (B) Quantitative data; n = 3 independent triplicate experiments.

(C and D) Immunocytochemistry of cTnT and DAPI after 4 weeks. Cells were treated as described in (A). High-magnification views, as insets, show sarcomeric organization. (D) Quantitative data; n = 3 independent triplicate experiments.

(E) Quantitative data for the number of spontaneously beating cells after 4 weeks; n = 3 independent triplicate experiments.

(F) Heatmap of microarray data illustrating the global gene expression pattern of fibroblasts 2 weeks after transduction with GHMT/scramble shRNA, GHMT/shYAP, or GHMT/shTAZ cultured on 126 kPa hydrogels. Differentially expressed genes are shown; n = 2 independent biological replicates.

(G and H) Venn diagram showing genes upregulated after GHMT/shYAP and GHMT/shTAZ transduction by more than 2-fold compared with that of GHMT/scramble shRNA. (H) GO analysis of the 135 upregulated genes of the GHMT/shYAP and GHMT/shTAZ groups. Cardiac-related GO terms are shown.

(I and J) Venn diagram showing genes downregulated after GHMT/shYAP and GHMT/shTAZ transduction by more than 2-fold compared with that of GHMT/scramble shRNA. (J) GO analysis of the 472 downregulated genes in both GHMT/shYAP and GHMT/shTAZ groups. Fibroblast-related GO terms are shown.

(K and L) qRT-PCR analysis to determine relative mRNA expression of cardiomyocyte, fibroblast, and YAP/TAZ target genes in the (K) GHMT/shYAP and (L) GHMT/shTAZ groups compared with GHMT/scramble shRNA groups; n = 3 independent triplicate experiments.

All experiments were performed on 126 kPa hydrogels. All data are presented as the means ± SD. $*p < 0.05$ versus the relevant control. Scale bars represent 100 μm.

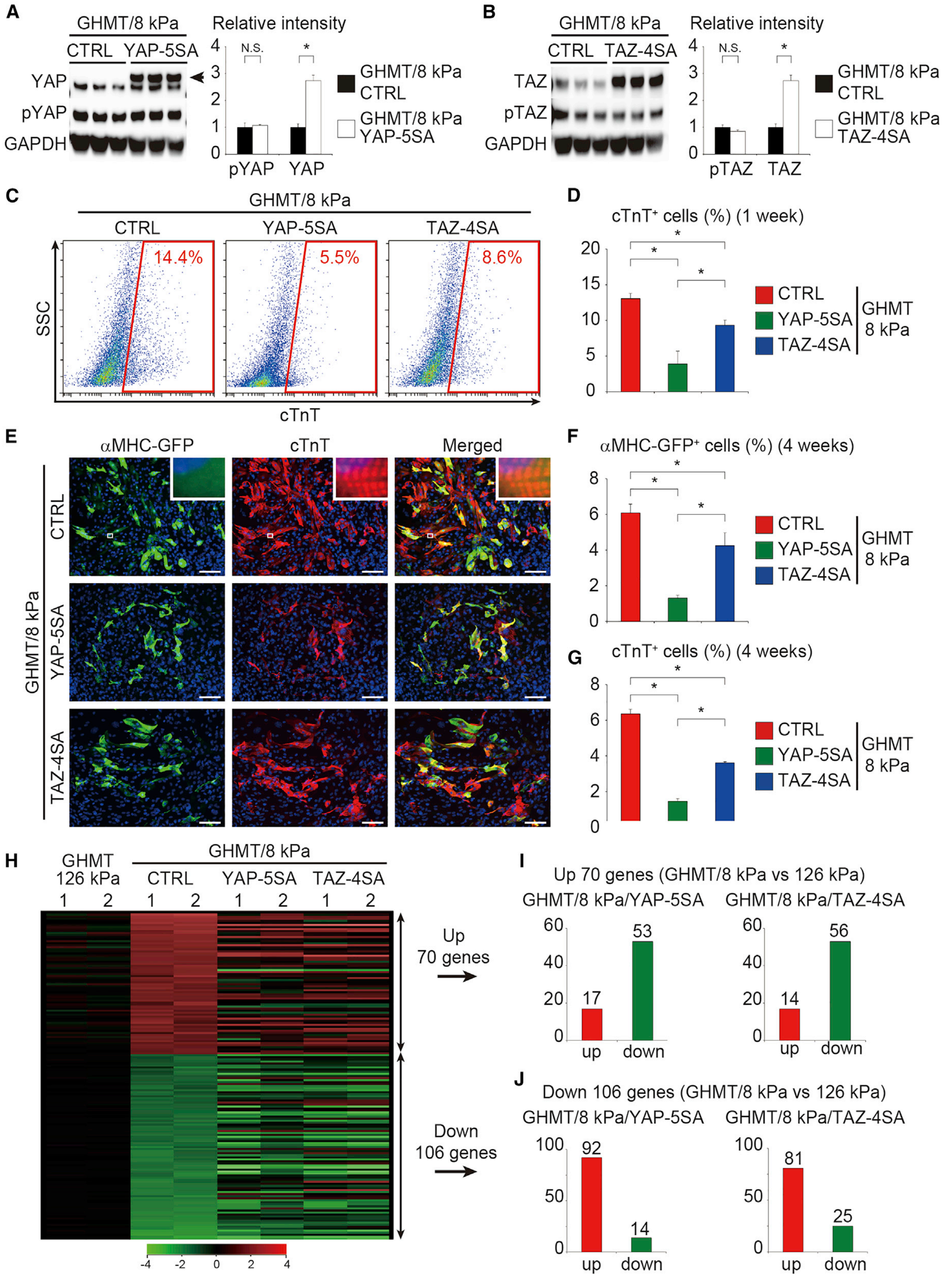

(legend on next page)

Furthermore, expression of activated YAP/TAZ inhibited the generation of beating iCMs after 4 weeks, overriding the positive effects of soft ECM-mediated cardiac reprogramming (Figure S3A). Next, we performed the 5-ethynyl-2′-deoxyuridine (EdU) incorporation assay to determine cell proliferation during cardiac reprogramming. A 2-week pulse labeling with EdU demonstrated that almost all iCMs were post-mitotic and YAP-5SA and TAZ-4SA did not augment iCM proliferation (Figures S3B and S3C). We next examined whether overexpression of YAP-5SA and TAZ-4SA can globally counteract soft ECM-mediated gene regulation. Microarray analyses revealed that 53 and 56 out of 70 genes upregulated by 8 kPa hydrogels were suppressed, while 92 and 81 out of 106 downregulated genes were upregulated by the overexpression of YAP-5SA and TAZ-4SA, respectively (Figures 4H–4J); this suggests that transcriptional changes affected by soft ECM were largely mediated via suppression of YAP/TAZ signaling. These results indicate that soft ECM promotes cardiac reprogramming at least in part via suppression of YAP/TAZ signaling.

Integrin, Rho/ROCK, Actomyosin, and YAP/TAZ Signaling Are Suppressed in iCMs Cultured on Soft Substrates

We next investigated the upstream mechanotransduction pathway regulating YAP/TAZ activity in soft ECM-induced cardiac reprogramming. It has been reported that integrin receptors, Rho/Rho-associated protein kinase (ROCK), actomyosin organization, and YAP/TAZ signaling are activated when cells are cultured on hard substrates, yet are suppressed when cells are cultured on soft substrates (Dupont, 2016; Dupont et al., 2011). Consistent with this, microarray and qRT-PCR analyses revealed that genes related to integrin-mediated signaling and *Itgb2* were downregulated in GHMT-transduced cells cultured on 8 kPa hydrogels more than those on 126 kPa (Figures 2B and 2D). Furthermore, formation of phalloidin$^+$ stress fibers (F-actin, activated actin) was disrupted in cells cultured on 8 kPa hydrogels, concomitant with a reduction of YAP/TAZ nuclear localization (Figure S4A). Thus, integrin signaling, actomyosin organization, and YAP/TAZ activity were suppressed during cardiac reprogramming by culturing the cells on 8 kPa soft ECM.

Next, to determine the role of integrin, Rho/ROCK, and actomyosin signaling in cardiac reprogramming, we treated GHMT-transduced fibroblasts on 126 kPa substrates with specific inhibitors of integrin signaling (RGDS peptide, PF-00562271), ROCK (Y-27632), and myosin II (blebbistatin). Inhibition of integrin signaling with RGDS peptide and PF-00562271 significantly increased the number of cTnT$^+$ cells, as shown by FACS analysis (Figures 5A and 5B). Blebbistatin is a reversible inhibitor of all myosin II isoforms that are expressed in striated muscle (including CMs), smooth muscle, and non-muscle cells (including fibroblasts) (Yahalom-Ronen et al., 2015). Consistent with the critical function of Rho/ROCK and myosin II in cytoskeleton tension, both inhibitors disrupted phalloidin$^+$ stress fibers and led to reduced YAP/TAZ nuclear localization in GHMT-transduced fibroblasts (Figures 5C and 5D). FACS analyses demonstrated that cTnT$^+$ cells were significantly increased with the inhibitor treatment after 1 week, suggesting that inhibition of the Rho/ROCK and actomyosin pathways promotes cardiac reprogramming on 126 kPa and PS substrates (Figures 5E, 5F, S4B, and S4C). In contrast, these inhibitors did not significantly increase cardiac reprogramming on 8 kPa hydrogels, suggesting that the effects of these inhibitors are mainly dependent on matrix stiffness (Figures S4D and S4E). Inhibition of ROCK and myosin II for the first 2 weeks was sufficient to increase the generation of αMHC-GFP$^+$ and cTnT$^+$ cells and beating iCMs by ~2- to 3-fold after 4 weeks on 126 kPa hydrogels (Figures 5G–5I). EdU assay showed that inhibition of ROCK and myosin II did not increase iCM proliferation (Figures S4F and S4G), suggesting that these inhibitors promoted cardiac reprogramming mainly by modifying the iCM differentiation process. Sustained blebbistatin treatment for 4 weeks suppressed the

Figure 4. Activation of YAP/TAZ Inhibits Soft Matrix-Mediated Cardiac Reprogramming

(A and B) Western blot analysis of total YAP and TAZ, pYAP, and pTAZ levels in GHMT-transduced fibroblasts treated with (A) YAP-5SA and (B) TAZ-4SA for 72 h (n = 3 independent triplicate experiments). Arrow indicates exogenous YAP expression. Relative intensity was normalized to the levels in GHMT-transduced fibroblasts cultured on 8 kPa hydrogels.

(C and D) FACS analysis of cTnT expression after 1 week. GHMT-induced fibroblasts cultured on 8 kPa hydrogels were transduced with YAP-5SA and TAZ-4SA. YAP-5SA and TAZ-4SA suppressed the generation of iCMs. (D) Quantitative data; n = 3 independent triplicate experiments.

(E–G) Immunocytochemistry of αMHC-GFP, cTnT, and DAPI after 4 weeks. High-magnification views, as insets, show sarcomeric organization. (F and G) Quantitative data; n = 3 independent triplicate experiments.

(H–J) Heatmap of microarray data illustrating the global gene expression pattern of fibroblasts 2 weeks after transduction with GHMT on 126 kPa (GHMT/126 kPa), GHMT on 8 kPa (GHMT/8 kPa/CTRL), GHMT/YAP-5SA on 8 kPa (GHMT/8 kPa/YAP-5SA), and GHMT/TAZ-4SA on 8 kPa (GHMT/8 kPa/TAZ-4SA) hydrogels (n = 2 independent biological replicates). Gene expression changes induced by culturing on 8 kPa hydrogels were largely reversed by overexpression of (I) YAP-5SA and (J)TAZ-4SA.

All data are presented as the means ± SD. *p < 0.05 versus the relevant control. Scale bars represent 100 μm.

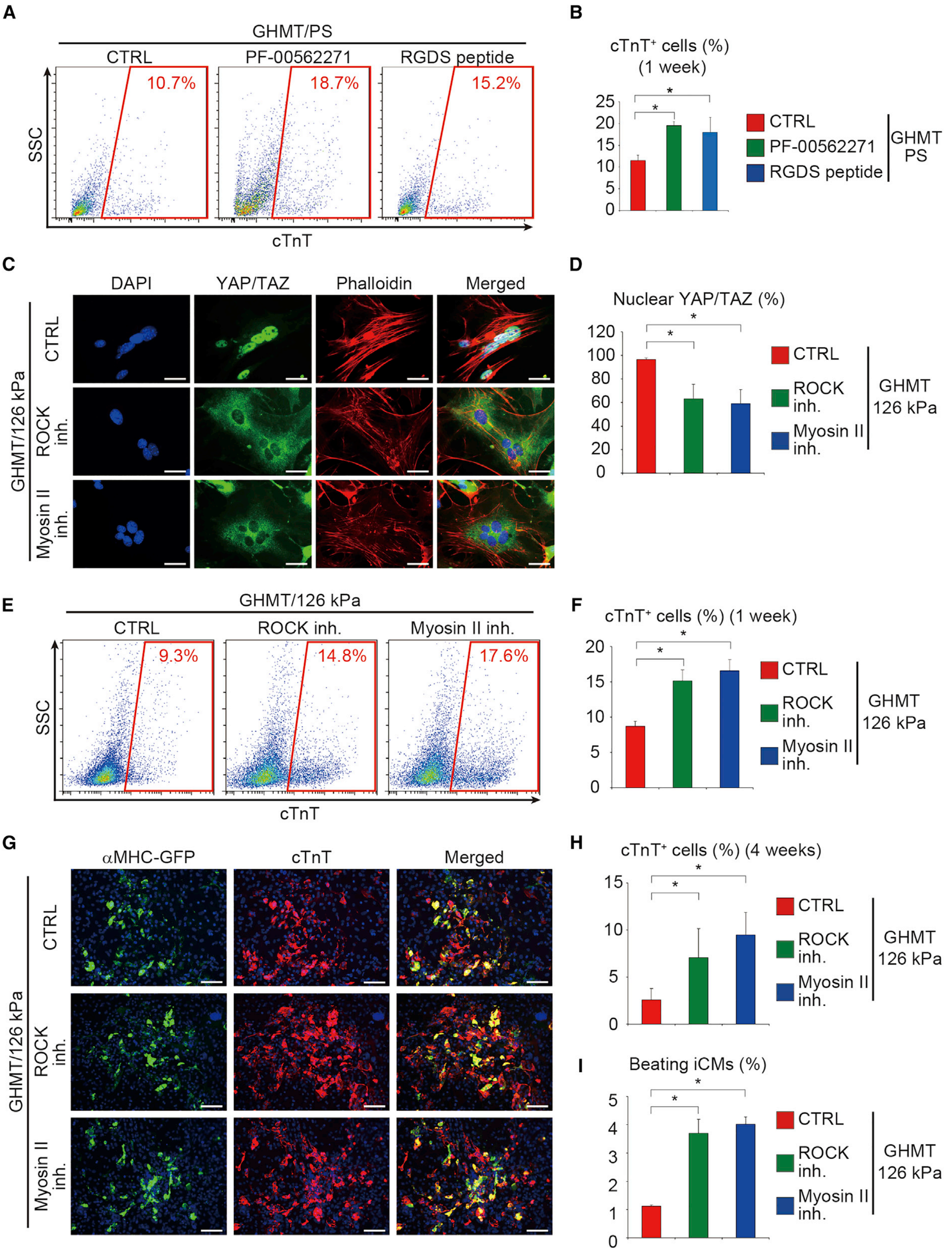

(legend on next page)

generation of beating iCMs, consistent with the fact that cardiomyocyte contraction requires active cardiac myosin II (Lyon et al., 2015; Yahalom-Ronen et al., 2015). Thus, transient blebbistatin treatment targeted non-muscle myosin II in fibroblasts and promoted cardiac reprogramming. These results suggest that inhibition of the integrin, Rho/ROCK, and actomyosin pathway promoted cardiac reprogramming with suppression of YAP/TAZ signaling.

Inhibition of Rho/ROCK and Actomyosin Promotes Cardiac Reprogramming via Suppression of YAP/TAZ

Next, to investigate whether inhibition of Rho/ROCK and actomyosin promotes cardiac reprogramming through suppression of the downstream targets YAP/TAZ, we asked whether constitutive activation of YAP/TAZ overrides the positive effects of ROCK or myosin II inhibitor-induced cardiac reprogramming. FACS analyses demonstrated that expression of activated YAP and TAZ suppressed the generation of cTnT$^+$ cells in either ROCK or myosin II inhibitor-treated cells on 126 kPa after 1 week (Figures 6A–6D). Immunocytochemistry also demonstrated that activation of YAP or TAZ suppressed ROCK or myosin II inhibitor-mediated cardiac reprogramming after 4 weeks (Figures 6E, 6F, 6H, and 6I). Consistently, the number of beating iCMs was also reduced after transduction with YAP-5SA or TAZ-4SA, overriding the positive effects of ROCK and myosin II inhibitors on cardiac reprogramming (Figures 6G and 6J). Expression of the constitutively active YAP/TAZ also inhibited cardiac reprogramming of fibroblasts cultured on 8 kPa hydrogels and treated with ROCK and myosin II inhibitors (Figures S5A–S5J). These results demonstrate that inhibition of the Rho/ROCK and actomyosin pathways promotes cardiac reprogramming via suppression of YAP/TAZ.

Soft ECM Promoted Sendai Virus Vector-Mediated Cardiac Reprogramming via Suppression of YAP/TAZ

Thus far, we demonstrated that soft ECM and suppression of YAP/TAZ promoted cardiac reprogramming induced by the retroviral vectors expressing GHMT. It has been reported that Sendai virus (SeV) vectors reprogrammed fibroblasts on rigid PS dishes into integration-free iCMs more efficiently than the pMX retrovirus vectors (Miyamoto et al., 2018). Therefore, to test the universality of soft substrate effects and YAP/TAZ signaling on cardiac reprogramming, we investigated whether 8 kPa soft ECM would promote cardiac reprogramming using the SeV vector system expressing GMT (SeV-GMT). We found that, on PS dishes, SeV-GMT induced a higher number of spontaneously beating iCMs than pMX-GMT, and the 8 kPa substrate further increased the SeV-mediated cardiac reprogramming efficiency up to ~15% (Figure 7A). Immunocytochemistry also demonstrated that, on PS dishes, SeV-GMT generated more α-actinin$^+$ cells than pMX-GMT, and this increase was further enhanced by culturing on soft ECM (Figures 7B and 7C). Western blotting confirmed that fibroblasts cultured on 8 kPa hydrogels and infected with SeV-GMT had higher GMT protein levels than the cells infected with pMX-GMT. The total and phosphorylated YAP and TAZ protein expression was suppressed in both groups compared with the cells on PS dishes (Figures 2E and 7D). Next, to determine whether soft ECM promoted SeV-GMT-mediated cardiac reprogramming by suppressing YAP/TAZ, we overexpressed YAP-5SA and TAZ-4SA in SeV-GMT-transduced fibroblasts plated on 8 kPa hydrogel. After 4 weeks, constitutively active YAP/TAZ inhibited the generation of beating iCMs, overriding the positive effects of soft ECM-mediated cardiac reprogramming by SeV-GMT (Figure 7E). Immunocytochemistry for α-actinin further confirmed that overexpression of YAP-5SA and TAZ-4SA suppressed the generation of iCMs (Figures 7F and 7G). Therefore, soft ECM promoted SeV-GMT-mediated cardiac programming by suppressing YAP/TAZ signaling.

DISCUSSION

We developed a Matrigel-based hydrogel culture system that recapitulates the stiffness of native tissues to dissect

Figure 5. Inhibition of Integrin Signaling, ROCK, and Myosin II Promotes Cardiac Reprogramming on Rigid Substrates

(A and B) FACS analysis of cTnT expression after 1 week of culture. GHMT-induced fibroblasts cultured on PS dishes were treated with integrin signaling inhibitors. (B) Quantification of data presented in (A); n = 3 independent triplicate experiments.

(C and D) Immunocytochemistry of YAP/TAZ, phalloidin, and DAPI. GHMT-transduced fibroblasts were treated with ROCK and myosin II inhibitors, cultured on 126 kPa hydrogels, and analyzed 3 h after inhibitor treatment. (D) Quantitative data; n = 4 independent triplicate experiments.

(E and F) FACS analysis of cTnT expression after 1 week. Cells were treated as described in (C). (F) Quantitative data; n = 3 independent triplicate experiments.

(G and H) Immunocytochemistry of αMHC-GFP, cTnT, and DAPI after 4 weeks. The ROCK inhibitor Y-27632 was used to supplement the medium for 4 weeks, whereas the myosin II inhibitor blebbistatin was used to supplement the medium for the first 2 weeks. (H) Quantitative data; n = 3 independent triplicate experiments.

(I) Quantitative data of the number of spontaneously beating cells after 4 weeks. Cells were treated as described in (C); n = 4 independent triplicate experiments.

All data are presented as the means ± SD. *p < 0.05 versus the relevant control. inh, inhibitor. Scale bars represent 20 μm (C), 100 μm (G).

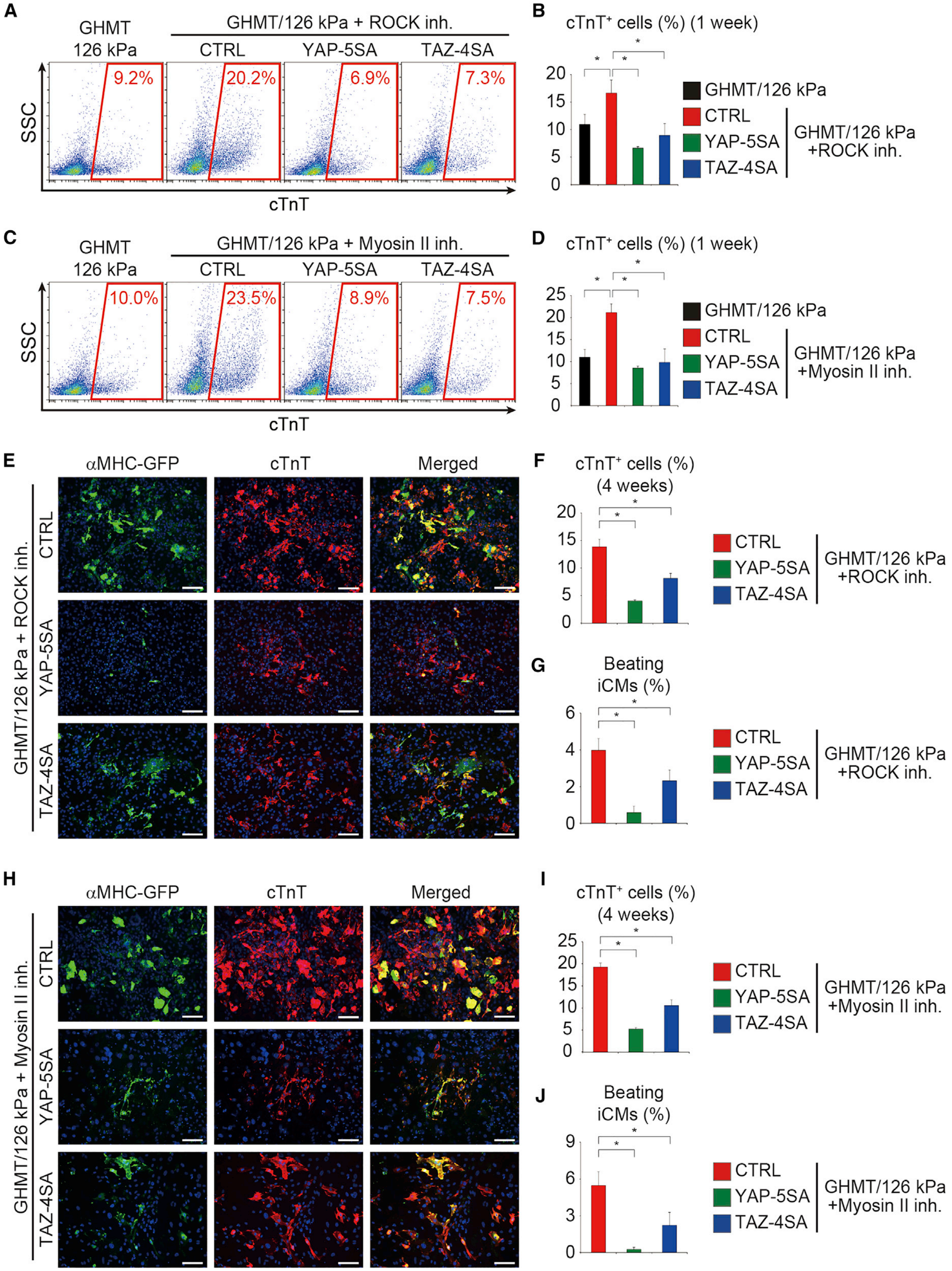

(legend on next page)

the role of biophysical cues in cardiac reprogramming. Our results demonstrated, for the first time, that soft matrices comparable with native myocardium improved cardiac reprogramming by suppressing the YAP/TAZ signaling and silencing fibroblast gene programs, which were activated by conventional culture on rigid substrates. Thus, we identified a new link between mechanobiology and direct cardiac reprogramming, which may advance our understanding of cellular biology and optimize biomaterials for clinical translation.

Previous *in vitro* cardiac reprogramming studies mostly used PS dishes, which were much stiffer than native myocardium (Addis et al., 2013; Ieda et al., 2010; Ifkovits et al., 2014; Jayawardena et al., 2012; Mohamed et al., 2017; Muraoka et al., 2014; Sadahiro et al., 2015; Song et al., 2012; Srivastava and Ieda, 2012; Wada et al., 2013; Yamakawa et al., 2015; Zhao et al., 2015; Zhou et al., 2015). Thus, our hydrogel culture system was necessary for identifying optimal matrix stiffness and revealing the roles of biophysical factors in cardiac reprogramming. The preferred modulus for cardiac reprogramming identified—8 kPa—is near the value of the elastic modulus of adult rat heart (~10 kPa) (Berry et al., 2006; Engler et al., 2008; Herum et al., 2017), perhaps indicating that physiologically relevant substrate is most appropriate for cardiac reprogramming. Indeed, softer substrates (1–2 kPa) detached the GHMT-transduced iCMs from culture dishes, whereas harder substrates (~100 kPa) suppressed cardiac reprogramming via activation of YAP/TAZ and fibroblastic programs (Herum et al., 2017). We anticipate that our culture system will be equally useful in identifying optimal elasticity and key factors for other direct lineage conversions from fibroblasts, such as hepatocytes, neurons, and chondrocytes (Sadahiro et al., 2015; Srivastava and DeWitt, 2016).

Mechanistically, we found that soft substrates promoted cardiac reprogramming, at least in part, through inhibition of integrin, Rho/ROCK, actomyosin, and YAP/TAZ signaling and subsequent suppression of fibroblast programs. We also found that the canonical Hippo/LATS cascade does not appear to be involved in regulating YAP/TAZ activity in soft ECM-mediated cardiac reprogramming. Nuclear YAP/TAZ form complexes with the major transcription factor partner TEADs (the TEAD/TEF family transcription factors) to coactivate gene expression (Lin et al., 2017). Consistent with our results, a recent genome-wide study revealed that chromatin signatures at TEADs-binding loci rapidly changed from open to closed status during cardiac reprogramming (Stone et al., 2019), suggesting that the YAP/TAZ and TEADs pathway is suppressed during cardiac reprogramming. The experiments using SeV vectors revealed that soft ECM improved the efficiency of cardiac reprogramming up to ~15%, in part by suppressing YAP/TAZ, demonstrating the universality of the effects of soft ECM and YAP/TAZ signaling on cardiac reprogramming. Robust transgene expression also contributed to the efficient cardiac reprogramming using the SeV vectors, suggesting that the balance between the cardiogenic and fibroblast programs determines the efficiency of cardiac reprogramming. It is also possible that mechanical stimulation, actomyosin tension, and Rho/ROCK signaling regulate cardiac reprogramming not only via YAP/TAZ but also through other mechanisms, including myocardin-related transcription factors/serum response factor (SRF) and epigenetic remodeling (Crowder et al., 2016; Dupont et al., 2011; Olson and Nordheim, 2010; Sia et al., 2016). Further studies may provide new insights into the mechanism of iCM generation and improve the efficiency and quality of cardiac reprogramming.

A recent paper showed that, compared with GMT alone, delivery of small molecules (transforming growth factor β and Wnt inhibitors) in the presence of GMT improved *in vivo* cardiac reprogramming and enhanced cardiac repair after MI in mice (Mohamed et al., 2017). The stiffness of infarcted myocardium becomes several-fold harder (~50 kPa) than healthy myocardium due to the

Figure 6. Activation of YAP/TAZ Abolished the Effects of ROCK and Myosin II Inhibitors on Cardiac Reprogramming

(A and B) FACS analysis of cTnT expression after 1 week. GHMT-transduced cells cultured on 126 kPa hydrogels and treated with ROCK inhibitor were transduced with YAP-5SA and TAZ-4SA. Activation of YAP/TAZ suppressed ROCK inhibitor-mediated cardiac reprogramming. (B) Quantitative data; n = 3 independent triplicate experiments.

(C and D) FACS analysis of cTnT expression after 1 week. GHMT-transduced cells cultured on 126 kPa hydrogels and treated with myosin II inhibitor were transduced with YAP-5SA and TAZ-4SA. Activation of YAP/TAZ suppressed myosin II inhibitor-mediated cardiac reprogramming. (D) Quantitative data; n = 3 independent triplicate experiments.

(E, F, H, and I) Immunocytochemistry of αMHC-GFP, cTnT, and DAPI after 4 weeks. GHMT-fibroblasts cultured on 126 kPa hydrogels and treated with ROCK inhibitor for 4 weeks (E and F) and myosin II inhibitor for first 2 weeks (H and I) were transduced with YAP-5SA and TAZ-4SA. (F and I) Quantitative data; n = 3 independent triplicate experiments.

(G and J) Quantitative data of the number of spontaneously beating cells after 4 weeks (n = 4 independent triplicate experiments). Activation of YAP/TAZ suppressed ROCK (G) and myosin II inhibitor (J)-mediated cardiac reprogramming.

All experiments were performed on 126 kPa hydrogels. All data are presented as the means ± SD. $^*p < 0.05$ versus the relevant control. Scale bars represent 100 μm.

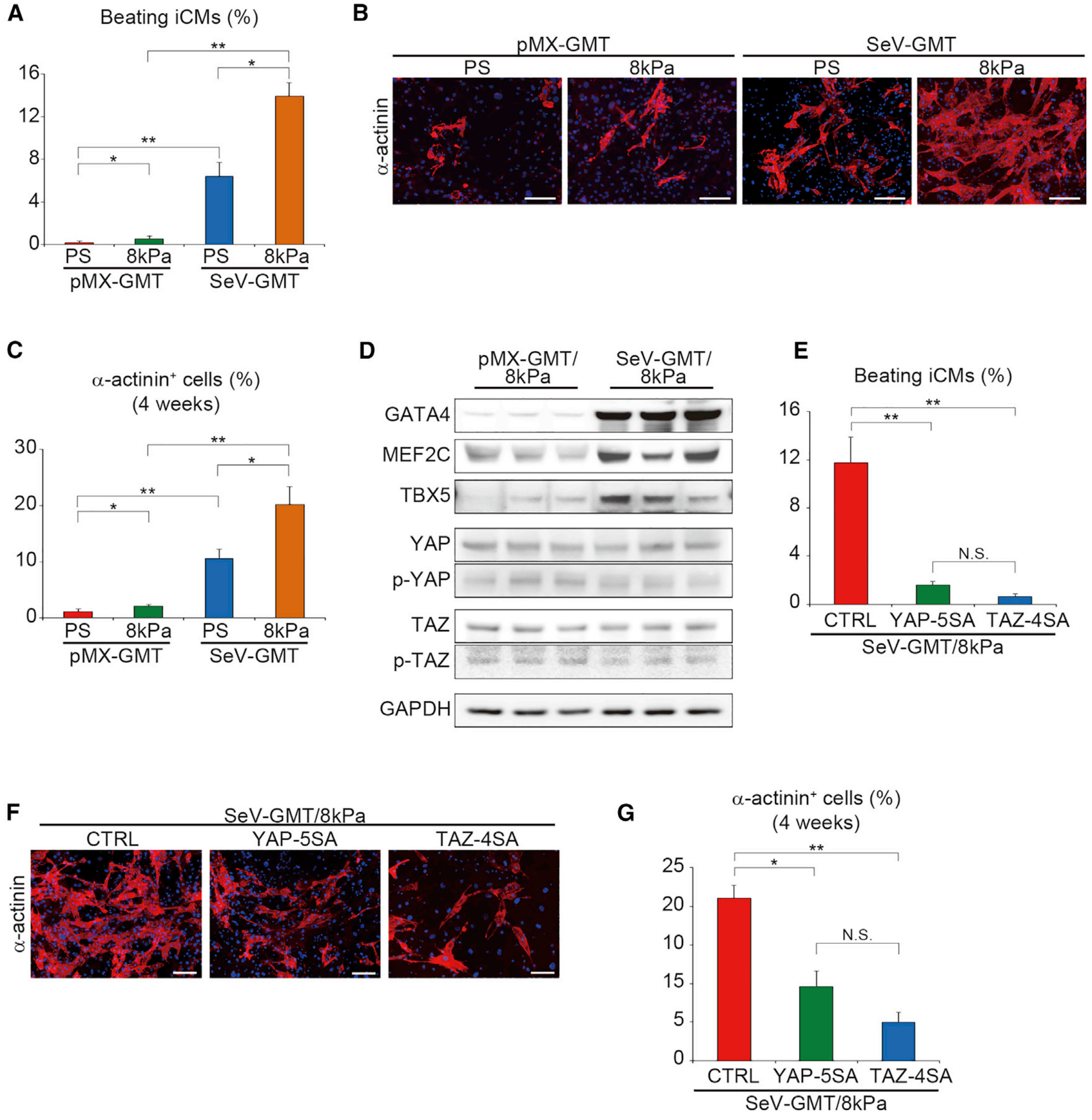

Figure 7. Soft Matrix Promoted SeV-Mediated Cardiac Reprogramming via YAP/TAZ Suppression

(A) Quantification of the spontaneously beating iCMs after 4 weeks of culture (n = 4 independent triplicate experiments). Fibroblasts were seeded on PS or 8 kPa hydrogels and transduced with pMX-GMT or SeV-GMT. Soft substrate enhanced SeV-mediated cardiac reprogramming.
(B) Immunocytochemistry of α-actinin and DAPI after 4 weeks. Cell were treated as shown in (A).
(C) Quantification of data presented in (B); n = 3 independent triplicate experiments.
(D) Western blot analysis for GMT, YAP/TAZ, and pYAP/pTAZ protein expression in pMX-GMT or SeV-GMT-transduced fibroblasts cultured on 8 kPa hydrogels; n = 3 independent biological replicates.
(E) Quantification of the number of spontaneously beating cells after 4 weeks; n = 4 independent triplicate experiments. Activation of YAP/TAZ suppressed SeV-mediated cardiac reprogramming.
(F) Immunocytochemistry of α-actinin and DAPI after 4 weeks.
(G) Quantification of data presented in (F); n = 3 independent triplicate experiments.
All data are presented as the mean ± SD. $^{*}p < 0.05$; $^{**}p < 0.01$ versus the corresponding control. Scale bars represent 100 μm.

accumulation of newly synthesized ECM and fibrosis (Berry et al., 2006; Engler et al., 2008). Our results suggest that a matrix stiffer than 8 kPa may inhibit cardiac reprogramming. Thus, it would be interesting to determine whether inhibition of Rho/ROCK-actomyosin-YAP/TAZ signaling and fibrolytic treatment may promote cardiac reprogramming *in vivo* and improve cardiac function after MI.

In summary, we developed a Matrigel-based hydrogel culture system to determine the effects of physical cues and mechanotransduction on cardiac reprogramming. Recapitulating *in vivo* environments using biomaterials will allow for identification of new targets to advance cardiac reprogramming technology toward clinical applications.

EXPERIMENTAL PROCEDURES

Matrigel-Based Hydrogel Culture System

Polyacrylamide gel solutions were prepared with different concentrations of acrylamide (AAm; 00809-85; Nacalai Tesque, Kyoto, Japan) and N,N′-methylenebisacrylamide (BIS; 22402-02; Nacalai Tesque) to vary elasticity(Yip et al., 2013). For conjugation of Matrigel (356230; Corning Inc., Corning, NY) components, 6-acrylamidohexanoic acid (ACA; A1896; Tokyo Chemical Industry, Tokyo, Japan) solution (500 mM, pH 7) was added to the AAm-BIS mixture to a final concentration of 100 mM. AAm and BIS concentrations and the corresponding Young's modulus are shown in Table S3. Polymerization was initiated with 0.05% ammonium peroxodisulfate (APS; 02627-21; Nacalai Tesque) and catalyzed with 0.2% N,N,N′,N′-tetramethylethylenediamine (TEMED; 33401-72; Nacalai Tesque). The polymerizing solutions (1 mL) were gently poured into the gap of slide glasses (76 mm × 52 mm; S092240; Matsunami; Osaka, Japan) spaced with a 0.3-mm silicone membrane (SR-50; Tigers Polymer Association, Osaka, Japan). After polymerization, the gels were fully hydrated in 0.1 M 2-(N-morpholino) ethanesulfonic acid (MES) buffer (pH 6.1; 21623-26; Nacalai Tesque) overnight. Next, the gels were cut to the same size as PS dish bottoms. To conjugate the Matrigel components to the gel surface, carboxyl groups of the copolymerized ACA were activated with 0.5 M N-hydroxysuccinimide (NHS; 18948-44; Nacalai Tesque) and 0.2 M 1-ethyl-3-(3-dimethylaminopropyl) carbodiimide hydrochloride (EDAC; 346-03632; Wako) in 0.1 M MES buffer (pH 6.1) for 30 min at room temperature, washed with cold 60% methanol/PBS for 2 h at 4°C, and reacted overnight with 0.05 mg/mL Matrigel diluted in PBS at 4°C. After washing three times with cold PBS overnight at 4°C, the gels were placed on the bottom of PS dishes and exposed to UV light in a sterile hood for 30 min, during which the gels were tightly attached to the dish bottoms. Before plating the cells, gel-attached dishes were equilibrated in DMEM supplemented with 10% fetal bovine serum for at least 30 min at 37°C. Conventional PS dishes were coated with Matrigel according to manufacturer's instructions.

Data and Code Availability

The data that support the findings of this study are available from the corresponding author upon request. The microarray data reported in this paper have been uploaded to the NCBI GEO under the accession number GSE125811.

SUPPLEMENTAL INFORMATION

Supplemental Information can be found online at https://doi.org/10.1016/j.stemcr.2020.07.022.

AUTHOR CONTRIBUTIONS

S.K., T. Sadahiro, and M. Ieda designed the experiments. S.K., T. Sadahiro, R.F., H.T., H.Y., F.T., M. Isomi, H.K., Y.Y., Y.A., Y.M., T.A., and N.M. carried out the experiments. S.K., T. Sadahiro, I.H., T. Suzuki, and K.F. analyzed the data. S.K., T. Sadahiro, and M. Ieda wrote the paper.

CONFLICTS OF INTERESTS

S.K. is the employee of Otsuka Pharmaceutical Co., Ltd. I.H. is the employee of Canon Inc. M. Ieda holds a patent related to this work: U.S. Patent 9,517,250 entitled "Methods for Generating Cardiomyocytes," issued on October 19, 2012. Inventors: Deepak Srivastava and Masaki Ieda.

ACKNOWLEDGMENTS

M. Ieda was supported by research grants from the Advanced Research & Development Programs for Medical Innovation (solotype; JP18gm5810002), the Research Center Network for Realization of Regenerative Medicine (JP20bm0704030), the Japan Agency for Medical Research and Development (AMED), the Japan Society for the Promotion of Science (JSPS; 19K22613), and the Takeda Science Foundation. S.K. was supported by a research grant from the Japan Society for the Promotion of Science (JSPS; 17J06637). T. Sadahiro was supported by research grants from JSPS (18K08114), SENSHIN Medical Research Foundation, The Uehara Memorial Foundation, Mochida Memorial Foundation, and The Kanae Foundation. R.F. was supported by Leading Initiative for Excellent Young Researchers (MEXT), H.Y. was supported by The Vehicle Racing Commemorative Foundation and Japan Society for the Promotion of Science (JSPS; 18K08047).

Received: October 1, 2019
Revised: July 28, 2020
Accepted: July 28, 2020
Published: August 27, 2020

REFERENCES

Addis, R.C., Ifkovits, J.L., Pinto, F., Kellam, L.D., Esteso, P., Rentschler, S., Christoforou, N., Epstein, J.A., and Gearhart, J.D. (2013). Optimization of direct fibroblast reprogramming to cardiomyocytes using calcium activity as a functional measure of success. J. Mol. Cell. Cardiol. *60*, 97–106.

Berry, M.F., Engler, A.J., Woo, Y.J., Pirolli, T.J., Bish, L.T., Jayasankar, V., Morine, K.J., Gardner, T.J., Discher, D.E., and Sweeney, H.L. (2006). Mesenchymal stem cell injection after myocardial infarction improves myocardial compliance. Am. J. Physiol. Heart Circ. Physiol. *290*, H2196–H2203.

Crowder, S.W., Leonardo, V., Whittaker, T., Papathanasiou, P., and Stevens, M.M. (2016). Material cues as potent regulators of epigenetics and stem cell function. Cell Stem Cell *18*, 39–52.

Dupont, S. (2016). Role of YAP/TAZ in cell-matrix adhesion-mediated signaling and mechanotransduction. Exp. Cell Res. *343*, 42–53.

Dupont, S., Morsut, L., Aragona, M., Enzo, E., Giulitti, S., Cordenonsi, M., Zanconato, F., Le Digabel, J., Forcato, M., Bicciato, S., et al. (2011). Role of YAP/TAZ in mechanotransduction. Nature *474*, 179–183.

Engler, A.J., Sen, S., Sweeney, H.L., and Discher, D.E. (2006). Matrix elasticity directs stem cell lineage specification. Cell *126*, 677–689.

Engler, A.J., Carag-Krieger, C., Johnson, C.P., Raab, M., Tang, H.Y., Speicher, D.W., Sanger, J.W., Sanger, J.M., and Discher, D.E. (2008). Embryonic cardiomyocytes beat best on a matrix with heart-like elasticity: scar-like rigidity inhibits beating. J. Cell Sci. *121*, 3794–3802.

Herum, K.M., Choppe, J., Kumar, A., Engler, A.J., and McCulloch, A.D. (2017). Mechanical regulation of cardiac fibroblast profibrotic phenotypes. Mol. Biol. Cell *28*, 1871–1882.

Ieda, M., Fu, J.D., Delgado-Olguin, P., Vedantham, V., Hayashi, Y., Bruneau, B.G., and Srivastava, D. (2010). Direct reprogramming of fibroblasts into functional cardiomyocytes by defined factors. Cell *142*, 375–386.

Ifkovits, J.L., Addis, R.C., Epstein, J.A., and Gearhart, J.D. (2014). Inhibition of TGFbeta signaling increases direct conversion of fibroblasts to induced cardiomyocytes. PLoS One *9*, e89678.

Inagawa, K., Miyamoto, K., Yamakawa, H., Muraoka, N., Sadahiro, T., Umei, T., Wada, R., Katsumata, Y., Kaneda, R., Nakade, K., et al. (2012). Induction of cardiomyocyte-like cells in infarct hearts by gene transfer of Gata4, Mef2c, and Tbx5. Circ. Res. *111*, 1147–1156.

Isomi, M., Sadahiro, T., and Ieda, M. (2019). Progress and challenge of cardiac regeneration to treat heart failure. J. Cardiol. *73*, 97–101.

Jayawardena, T.M., Egemnazarov, B., Finch, E.A., Zhang, L., Payne, J.A., Pandya, K., Zhang, Z., Rosenberg, P., Mirotsou, M., and Dzau, V.J. (2012). MicroRNA-mediated in vitro and in vivo direct reprogramming of cardiac fibroblasts to cardiomyocytes. Circ. Res. *110*, 1465–1473.

Lian, I., Kim, J., Okazawa, H., Zhao, J., Zhao, B., Yu, J., Chinnaiyan, A., Israel, M.A., Goldstein, L.S., Abujarour, R., et al. (2010). The role of YAP transcription coactivator in regulating stem cell self-renewal and differentiation. Genes Dev. *24*, 1106–1118.

Lin, K.C., Park, H.W., and Guan, K.L. (2017). Regulation of the Hippo pathway transcription factor TEAD. Trends Biochem. Sci. *42*, 862–872.

Lyon, R.C., Zanella, F., Omens, J.H., and Sheikh, F. (2015). Mechanotransduction in cardiac hypertrophy and failure. Circ. Res. *116*, 1462–1476.

Miyamoto, K., Akiyama, M., Tamura, F., Isomi, M., Yamakawa, H., Sadahiro, T., Muraoka, N., Kojima, H., Haginiwa, S., Kurotsu, S., et al. (2018). Direct in vivo reprogramming with Sendai virus vectors improves cardiac function after myocardial infarction. Cell Stem Cell *22*, 91–103.e5.

Mohamed, T.M., Stone, N.R., Berry, E.C., Radzinsky, E., Huang, Y., Pratt, K., Ang, Y.S., Yu, P., Wang, H., Tang, S., et al. (2017). Chemical enhancement of in vitro and in vivo direct cardiac reprogramming. Circulation *135*, 978–995.

Moroishi, T., Hayashi, T., Pan, W.W., Fujita, Y., Holt, M.V., Qin, J., Carson, D.A., and Guan, K.L. (2016). The Hippo pathway kinases LATS1/2 suppress cancer immunity. Cell *167*, 1525–1539.e17.

Muraoka, N., Yamakawa, H., Miyamoto, K., Sadahiro, T., Umei, T., Isomi, M., Nakashima, H., Akiyama, M., Wada, R., Inagawa, K., et al. (2014). MiR-133 promotes cardiac reprogramming by directly repressing Snai1 and silencing fibroblast signatures. EMBO J. *33*, 1565–1581.

Nam, Y.J., Lubczyk, C., Bhakta, M., Zang, T., Fernandez-Perez, A., McAnally, J., Bassel-Duby, R., Olson, E.N., and Munshi, N.V. (2014). Induction of diverse cardiac cell types by reprogramming fibroblasts with cardiac transcription factors. Development *141*, 4267–4278.

Ohgushi, M., Minaguchi, M., and Sasai, Y. (2015). Rho-signaling-directed YAP/TAZ activity underlies the long-term survival and expansion of human embryonic stem cells. Cell Stem Cell *17*, 448–461.

Olson, E.N., and Nordheim, A. (2010). Linking actin dynamics and gene transcription to drive cellular motile functions. Nat. Rev. Mol. Cell Biol. *11*, 353–365.

Qian, L., Huang, Y., Spencer, C.I., Foley, A., Vedantham, V., Liu, L., Conway, S.J., Fu, J.D., and Srivastava, D. (2012). In vivo reprogramming of murine cardiac fibroblasts into induced cardiomyocytes. Nature *485*, 593–598.

Sadahiro, T., Isomi, M., Muraoka, N., Kojima, H., Haginiwa, S., Kurotsu, S., Tamura, F., Tani, H., Tohyama, S., Fujita, J., et al. (2018). Tbx6 induces nascent mesoderm from pluripotent stem cells and temporally controls cardiac versus somite lineage diversification. Cell Stem Cell *23*, 382–395.e5.

Sadahiro, T., Yamanaka, S., and Ieda, M. (2015). Direct cardiac reprogramming: progress and challenges in basic biology and clinical applications. Circ. Res. *116*, 1378–1391.

Sia, J., Yu, P., Srivastava, D., and Li, S. (2016). Effect of biophysical cues on reprogramming to cardiomyocytes. Biomaterials *103*, 1–11.

Song, K., Nam, Y.J., Luo, X., Qi, X., Tan, W., Huang, G.N., Acharya, A., Smith, C.L., Tallquist, M.D., Neilson, E.G., et al. (2012). Heart repair by reprogramming non-myocytes with cardiac transcription factors. Nature *485*, 599–604.

Srivastava, D., and DeWitt, N. (2016). In vivo cellular reprogramming: the next generation. Cell *166*, 1386–1396.

Srivastava, D., and Ieda, M. (2012). Critical factors for cardiac reprogramming. Circ. Res. *111*, 5–8.

Stone, N.R., Gifford, C.A., Thomas, R., Pratt, K.J.B., Samse-Knapp, K., Mohamed, T.M.A., Radzinsky, E.M., Schricker, A., Ye, L., Yu, P., et al. (2019). Context-specific transcription factor functions regulate epigenomic and transcriptional dynamics during cardiac reprogramming. Cell Stem Cell *25*, 87–102.e9.

Wada, R., Muraoka, N., Inagawa, K., Yamakawa, H., Miyamoto, K., Sadahiro, T., Umei, T., Kaneda, R., Suzuki, T., Kamiya, K., et al. (2013). Induction of human cardiomyocyte-like cells from fibroblasts by defined factors. Proc. Natl. Acad. Sci. U S A *110*, 12667–12672.

Yahalom-Ronen, Y., Rajchman, D., Sarig, R., Geiger, B., and Tzahor, E. (2015). Reduced matrix rigidity promotes neonatal cardiomyocyte dedifferentiation, proliferation and clonal expansion. eLife *4*, e07455.

Yamakawa, H., Muraoka, N., Miyamoto, K., Sadahiro, T., Isomi, M., Haginiwa, S., Kojima, H., Umei, T., Akiyama, M., Kuishi, Y., et al. (2015). Fibroblast growth factors and vascular endothelial growth factor promote cardiac reprogramming under defined conditions. Stem Cell Reports *5*, 1128–1142.

Yip, A.K., Iwasaki, K., Ursekar, C., Machiyama, H., Saxena, M., Chen, H., Harada, I., Chiam, K.H., and Sawada, Y. (2013). Cellular response to substrate rigidity is governed by either stress or strain. Biophys. J. *104*, 19–29.

Zhao, B., Wei, X., Li, W., Udan, R.S., Yang, Q., Kim, J., Xie, J., Ikenoue, T., Yu, J., Li, L., et al. (2007). Inactivation of YAP oncoprotein by the Hippo pathway is involved in cell contact inhibition and tissue growth control. Genes Dev. *21*, 2747–2761.

Zhao, Y., Londono, P., Cao, Y., Sharpe, E.J., Proenza, C., O'Rourke, R., Jones, K.L., Jeong, M.Y., Walker, L.A., Buttrick, P.M., et al. (2015). High-efficiency reprogramming of fibroblasts into cardiomyocytes requires suppression of pro-fibrotic signaling. Nat. Commun. *6*, 8243.

Zhou, H., Dickson, M.E., Kim, M.S., Bassel-Duby, R., and Olson, E.N. (2015). Akt1/protein kinase B enhances transcriptional reprogramming of fibroblasts to functional cardiomyocytes. Proc. Natl. Acad. Sci. U S A *112*, 11864–11869.

Stem Cell Reports, Volume *15*

Supplemental Information

Soft Matrix Promotes Cardiac Reprogramming via Inhibition of YAP/ TAZ and Suppression of Fibroblast Signatures

Shota Kurotsu, Taketaro Sadahiro, Ryo Fujita, Hidenori Tani, Hiroyuki Yamakawa, Fumiya Tamura, Mari Isomi, Hidenori Kojima, Yu Yamada, Yuto Abe, Yoshiko Murakata, Tatsuya Akiyama, Naoto Muraoka, Ichiro Harada, Takeshi Suzuki, Keiichi Fukuda, and Masaki Ieda

Figure S1

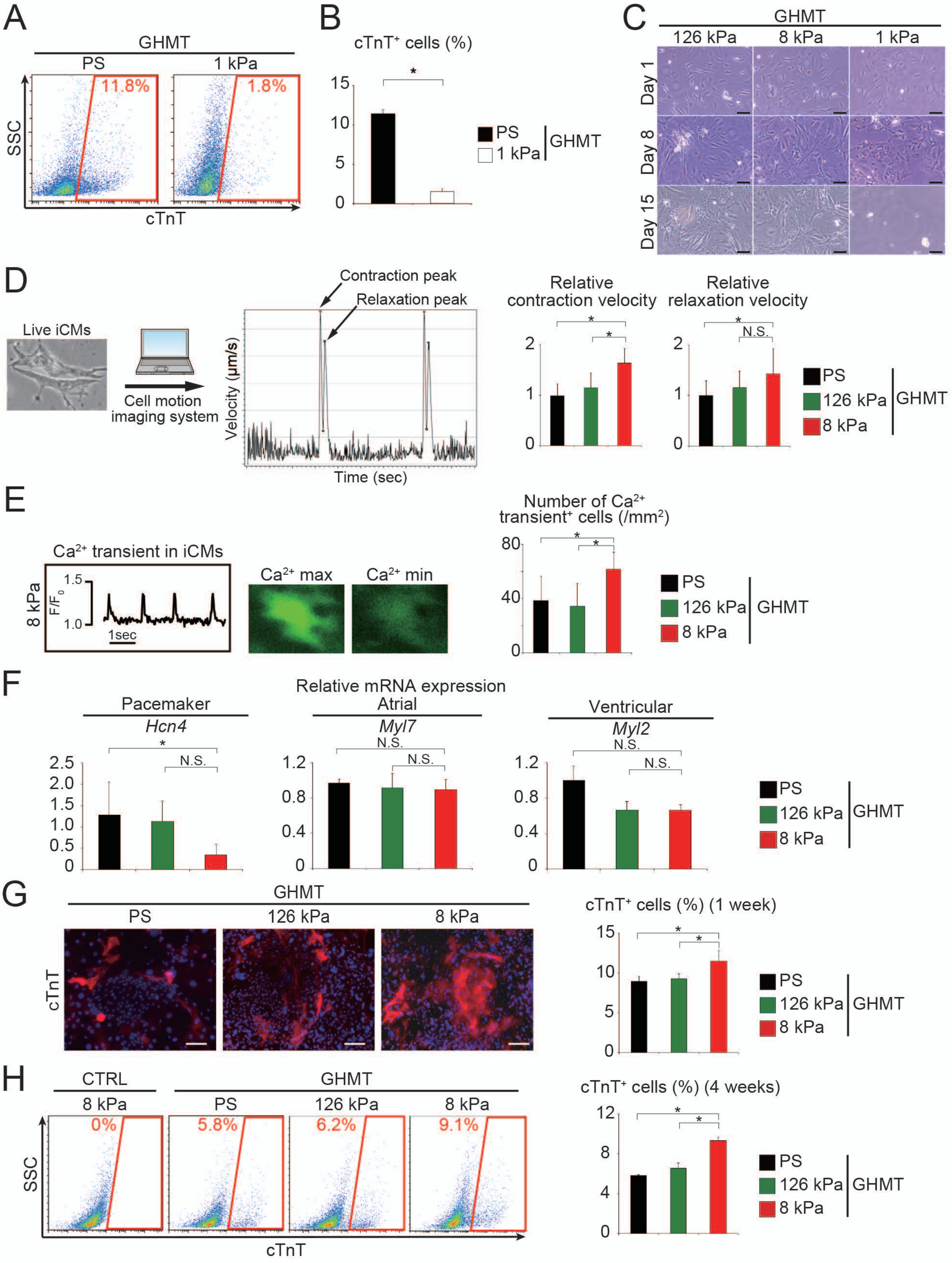

A
GHMT
PS
1 kPa
11.8%
1.8%
SSC
cTnT
B
cTnT+ cells (%)
15
10
5
0
*
PS
1 kPa
GHMT
C
GHMT
126 kPa
8 kPa
1 kPa
Day 1
Day 8
Day 15
D
Live iCMs
Cell motion imaging system
Contraction peak
Relaxation peak
Velocity (μm/s)
Time (sec)
Relative contraction velocity
Relative relaxation velocity
N.S.
PS
126 kPa
8 kPa
GHMT
E
Ca2+ transient in iCMs
8 kPa
F/F0
1.5
1.0
1sec
Ca2+ max
Ca2+ min
Number of Ca2+ transient+ cells (/mm2)
80
40
0
F
Relative mRNA expression
Pacemaker
Hcn4
Atrial
Myl7
Ventricular
Myl2
N.S.
2.5
2.0
1.5
1.0
0.5
1.2
0.8
0.4
G
GHMT
PS
126 kPa
8 kPa
cTnT
cTnT+ cells (%) (1 week)
H
CTRL
8 kPa
GHMT
PS
126 kPa
8 kPa
0%
5.8%
6.2%
9.1%
SSC
cTnT
cTnT+ cells (%) (4 weeks)
12
8
4
0

Figure S2

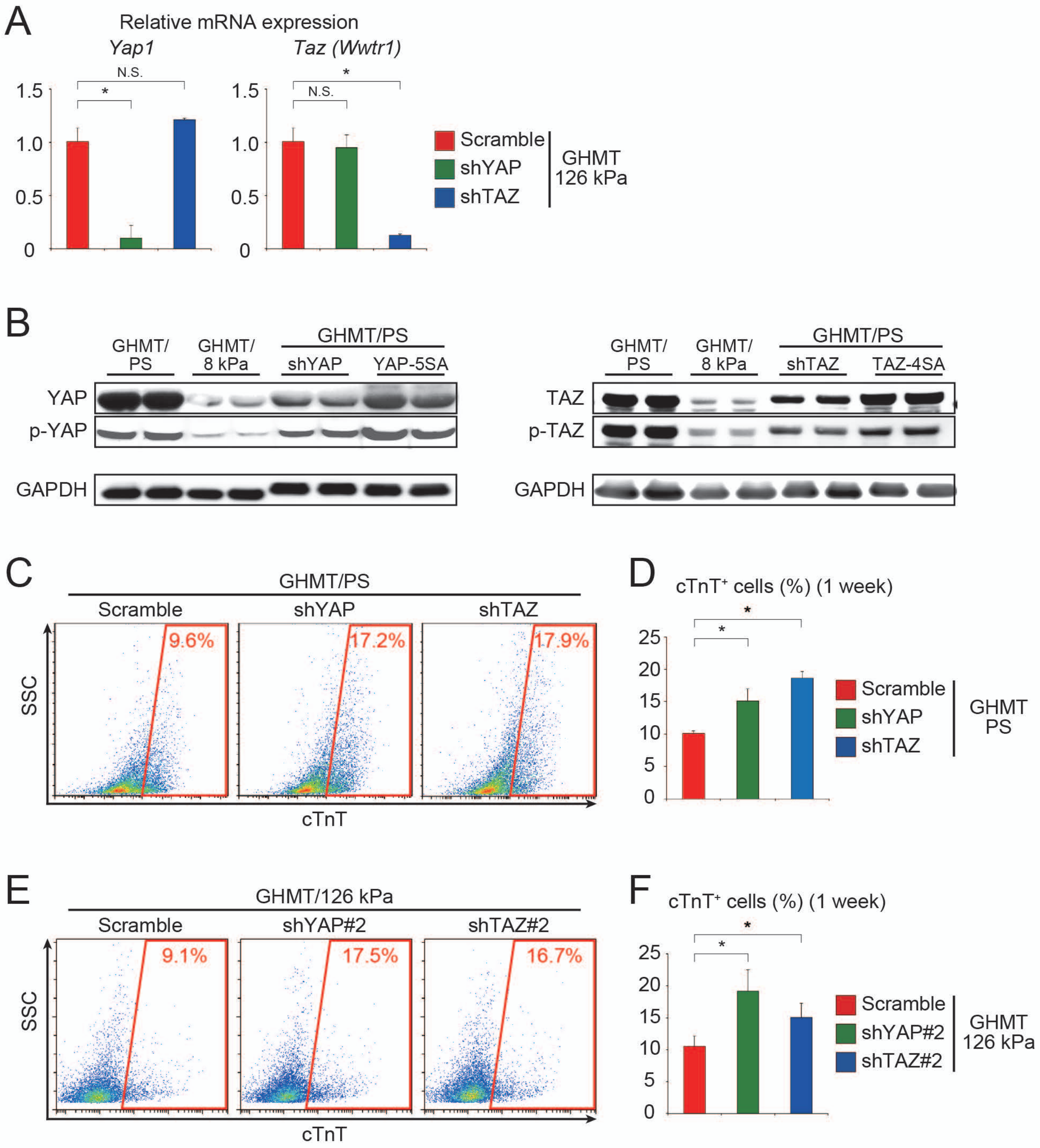

A
Relative mRNA expression
Yap1
Taz (Wwtr1)
N.S.
*
1.5
1.0
0.5
0
Scramble
shYAP
shTAZ
GHMT
126 kPa
B
GHMT/
PS
GHMT/
8 kPa
GHMT/PS
shYAP
YAP-5SA
YAP
p-YAP
GAPDH
shTAZ
TAZ-4SA
TAZ
p-TAZ
C
GHMT/PS
Scramble
shYAP
shTAZ
9.6%
17.2%
17.9%
SSC
cTnT
D
cTnT+ cells (%) (1 week)
25
20
15
10
5
0
GHMT
PS
E
GHMT/126 kPa
shYAP#2
shTAZ#2
9.1%
17.5%
16.7%
F

Figure S3

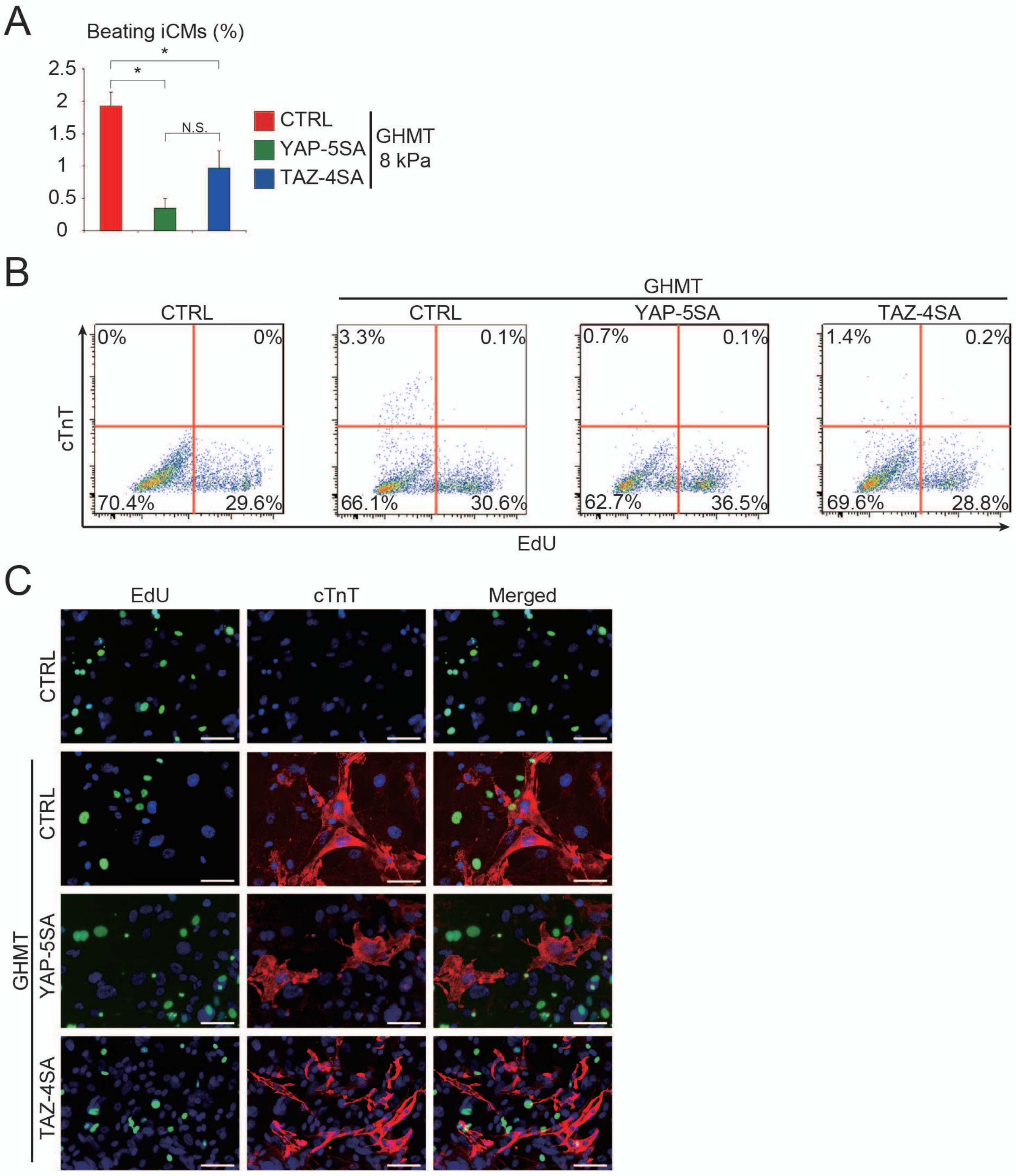
A
Beating iCMs (%)
2.5
2
1.5
1
0.5
0
*
*
N.S.
CTRL
YAP-5SA
TAZ-4SA
GHMT
8 kPa
B
GHMT
CTRL
CTRL
YAP-5SA
TAZ-4SA
cTnT
EdU
0%
0%
70.4%
29.6%
3.3%
0.1%
66.1%
30.6%
0.7%
0.1%
62.7%
36.5%
1.4%
0.2%
69.6%
28.8%
C
EdU
cTnT
Merged
CTRL
GHMT
CTRL
YAP-5SA
TAZ-4SA

Figure S4

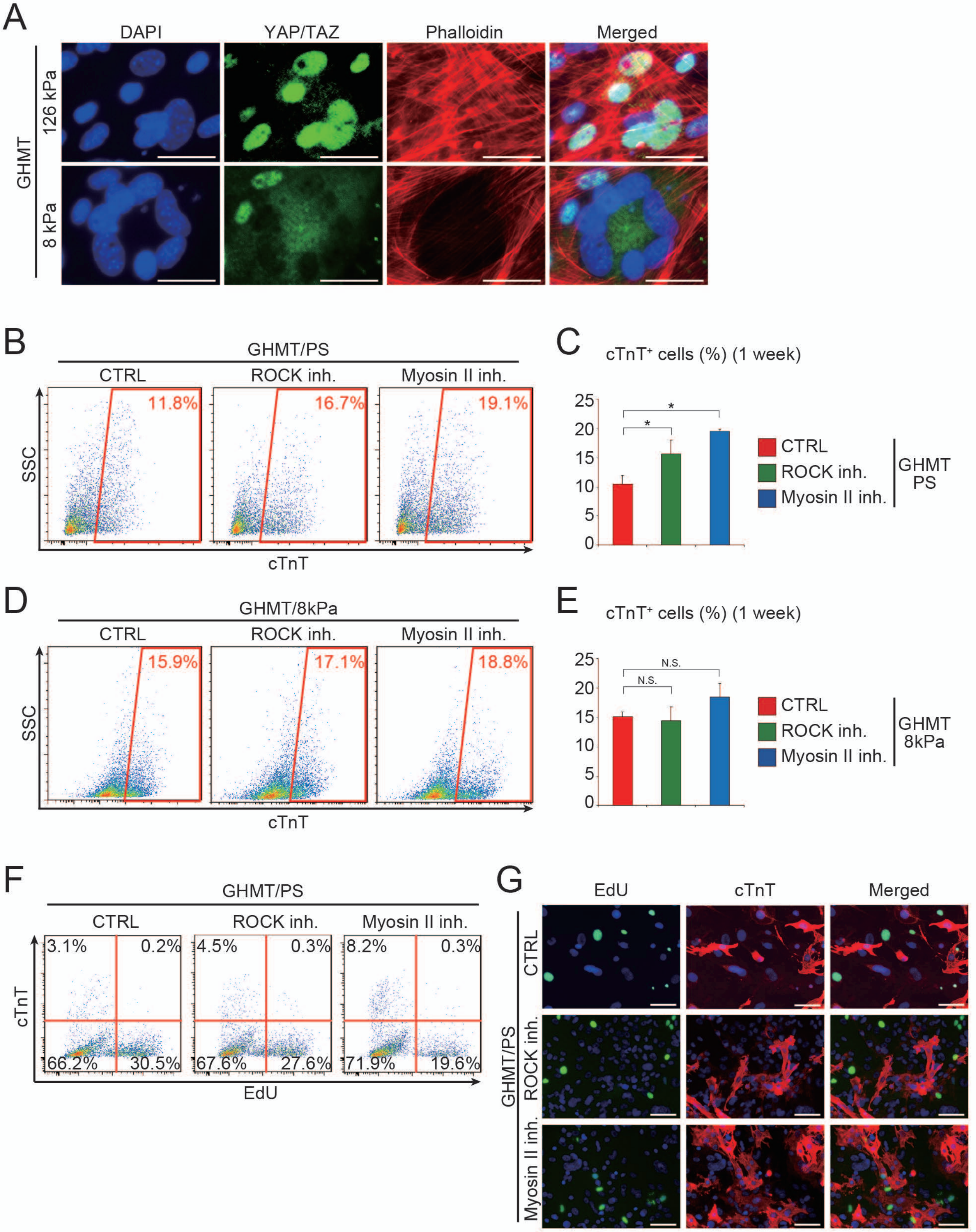
A
DAPI
YAP/TAZ
Phalloidin
Merged
GHMT
126 kPa
8 kPa
B
GHMT/PS
CTRL
ROCK inh.
Myosin II inh.
11.8%
16.7%
19.1%
SSC
cTnT
C
cTnT+ cells (%) (1 week)
25
20
15
10
5
0
*
*
CTRL
ROCK inh.
Myosin II inh.
GHMT
PS
D
GHMT/8kPa
CTRL
ROCK inh.
Myosin II inh.
15.9%
17.1%
18.8%
SSC
cTnT
E
cTnT+ cells (%) (1 week)
25
20
15
10
5
0
N.S.
N.S.
CTRL
ROCK inh.
Myosin II inh.
GHMT
8kPa
F
GHMT/PS
CTRL
ROCK inh.
Myosin II inh.
3.1%
0.2%
4.5%
0.3%
8.2%
0.3%
66.2%
30.5%
67.6%
27.6%
71.9%
19.6%
cTnT
EdU
G
EdU
cTnT
Merged
GHMT/PS
CTRL
ROCK inh.
Myosin II inh.

Figure S5

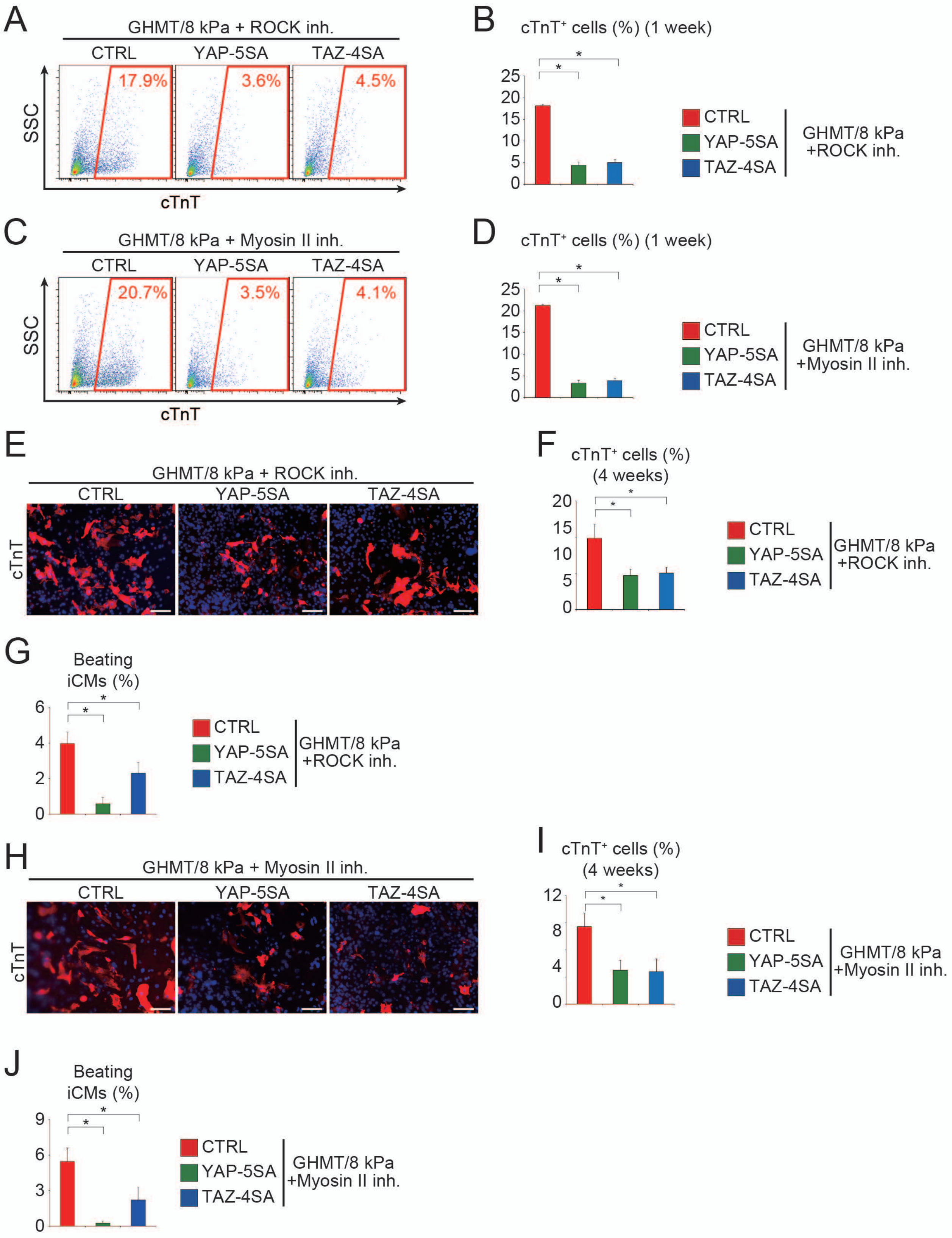

A
GHMT/8 kPa + ROCK inh.
CTRL
YAP-5SA
TAZ-4SA
17.9%
3.6%
4.5%
SSC
cTnT
B
cTnT+ cells (%) (1 week)
25
20
15
10
5
0
CTRL
YAP-5SA
TAZ-4SA
GHMT/8 kPa
+ROCK inh.
C
GHMT/8 kPa + Myosin II inh.
CTRL
YAP-5SA
TAZ-4SA
20.7%
3.5%
4.1%
SSC
cTnT
D
cTnT+ cells (%) (1 week)
CTRL
YAP-5SA
TAZ-4SA
GHMT/8 kPa
+Myosin II inh.
E
GHMT/8 kPa + ROCK inh.
CTRL
YAP-5SA
TAZ-4SA
cTnT
F
cTnT+ cells (%)
(4 weeks)
CTRL
YAP-5SA
TAZ-4SA
GHMT/8 kPa
+ROCK inh.
G
Beating
iCMs (%)
CTRL
YAP-5SA
TAZ-4SA
GHMT/8 kPa
+ROCK inh.
H
GHMT/8 kPa + Myosin II inh.
CTRL
YAP-5SA
TAZ-4SA
cTnT
I
cTnT+ cells (%)
(4 weeks)
CTRL
YAP-5SA
TAZ-4SA
GHMT/8 kPa
+Myosin II inh.
J
Beating
iCMs (%)
CTRL
YAP-5SA
TAZ-4SA
GHMT/8 kPa
+Myosin II inh.

Supplemental Information

Supplemental Figure Legends

Figure S1. Soft matrix improves quality of cardiac reprogramming. Related to Figure 1.
(A, B) FACS analysis of cTnT expression in GHMT-transduced fibroblasts cultured on PS and 1 kPa substrates. **(B)** Quantitative data; n = 3 independent triplicate experiments.
(C) Time course of bright-field images of GHMT-transduced fibroblasts cultured on 1 kPa, 8 kPa, and 126 kPa hydrogels. Note that the cell number was decreased under 1kPa hydrogel culture after 15 days.
(D) High-resolution motion capture tracking system for the iCM contraction and relaxation analyses at 4 weeks after GHMT transduction on different substrates. Relative contraction and relaxation peak velocities of the iCMs are shown on the right (n = 3, independent triplicate experiments).
(E) Calcium transients from spontaneously contracting iCMs at 6 weeks after transduction. Fluo-4 intensity trace is shown on the left panel; representative images of maximum and minimum concentrations of Ca^{2+} signals are shown in the middle panels. Quantification of the number of Ca^{2+} oscillation^{+} cells after 6 weeks is depicted on the right; n = 3, independent triplicate experiments.
(F) Relative mRNA expression in the GHMT-transduced cells cultured on different substrates determined by qRT-PCR (n = 3, independent triplicate experiments).
(G) Immunocytochemistry of cTnT and DAPI after 1 week. Quantification of cTnT^{+} cells; n = 3, independent triplicate experiments.
(H) FACS analysis of cTnT expression after 4 weeks. Quantification of cTnT^{+} cells; n = 3, independent triplicate experiments.
All data are presented as mean ± SD. *P < 0.05 versus the corresponding control. NS, not significant; CTRL, control. Scale bars = 100 µm.

Figure S2. shYAP and shTAZ enhanced cardiac reprogramming. Related to Figure 3.
(A) Relative mRNA expression levels of *Yap1* and *Taz (Wwtr1)* were determined by qRT-PCR after 72 h (n = 4 independent triplicate experiments).
(B) Western blot analysis of YAP/p-YAP (left), TAZ/p-TAZ (right), and GAPDH. Fibroblasts were transduced with GHMT, cultured on PS, 8 kPa, and PS, and treated with shYAP, YAP-5SA, shTAZ, and TAZ-4SA. shYAP and shTAZ downregulated YAP and TAZ protein expression, respectively (n = 2, independent biological replicates).
(C, D) FACS analysis of cTnT expression after 1 week. GHMT-transduced fibroblasts were cultured on PS and then transfected with scrambled shRNA, shYAP, and shTAZ. **(D)** Quantification of cTnT^{+} cells; n = 3, independent triplicate experiments.
(E, F) FACS analysis of cTnT expression after 1 week. GHMT-transduced fibroblasts cultured on 126 kPa hydrogels were transduced with Scramble shRNA and YAP and TAZ-specific shRNA (shYAP#2 and shTAZ#2). (F) Quantitative data; n = 3 independent triplicate experiments.
All data are presented as the means ± SD. *P < 0.05 versus the relevant control. NS, not significant.

Figure S3. Activation of YAP and TAZ suppressed soft ECM-mediated cardiac reprogramming. Related to Figure 4.
(A) Quantitative data of the number of spontaneously beating cells after 4 weeks. GHMT-fibroblasts cultured on 8 kPa hydrogels were transduced with YAP-5SA and TAZ-4SA (n = 4 independent triplicate experiments).
(B) FACS analysis of EdU incorporation. GHMT-transduced MEFs were transduced with mock, YAP-5SA, and TAZ-4SA.
(C) Representative images of immunostaining showing cTnT and EdU incorporation into GHMT-transduced MEFs transduced with mock, YAP-5SA, and TAZ-4SA. The majority of cTnT-positive cells were negative for EdU.
All data are presented as the means ± SD. *P < 0.05 versus the relevant control. CTRL, control.

Figure S4. Soft ECM suppressed actomyosin organization and YAP/TAZ acitivity. Related to Figure 5.
(A) Immunocytochemistry of YAP/TAZ, phalloidin, and DAPI after 96 h of transduction. Fibroblasts cultured on 126 and 8 kPa hydrogels were transduced with GHMT. Phalloidin^{+} stress fibre formation and nuclear YAP/TAZ expression were reduced in cells cultured on 8 kPa hydrogels.
(B, C) FACS analysis of cTnT expression in GHMT-transduced MEFs cultured on PS dishes and treated with ROCK or myosin II inhibitor for 1 week. **(C)** Quantification of cTnT^{+} cells; (n = 3, independent triplicate experiments).
(D, E) FACS analysis of cTnT expression in GHMT-transduced MEFs cultured on 8 kPa hydrogels and treated with ROCK or myosin II inhibitor for 1 week. **(E)** Quantification of cTnT^{+} cells; (n = 3 independent triplicate

experiments).
(F, G) EdU incorporation assays for GHMT-transduced MEFs treated with ROCK or myosin II inhibitor. FACS **(F)** and immunostaining **(G)** demonstrated that inhibition of ROCK and myosin II did not enhance cell proliferation of iCMs. Representative images are shown.
All data are presented as the means ± SD. *P < 0.05 versus the relevant control. NS, not significant; CTRL, control; inh, inhibitor. Scale bars = 20 µm (A), 100 µm (G).

Figure S5. Activation of YAP/TAZ suppressed cardiac reprogramming induced by soft ECM and inhibition of ROCK or myosin II. Related to Figure 6.
(A, B) FACS analysis of cTnT expression after 1 week. GHMT-transduced cells cultured on 8 kPa hydrogels and treated with ROCK inhibitor were transduced with YAP-5SA and TAZ-4SA. Activation of YAP/TAZ suppressed ROCK inhibitor-mediated cardiac reprogramming on 8 kPa ECM. **(B)** Quantification of $cTnT^+$ cells; n = 3, independent triplicate experiments.
(C, D) FACS analysis of cTnT expression after 1 week. GHMT-transduced cells cultured on 8 kPa hydrogels and treated with myosin II inhibitor were transduced with YAP-5SA and TAZ-4SA. Activation of YAP/TAZ suppressed myosin II inhibitor-mediated cardiac reprogramming on 8 kPa ECM. **(D)** Quantification of $cTnT^+$ cells; n = 3, independent triplicate experiments.
(E, F) Immunocytochemistry of cTnT and DAPI after 4 weeks. YAP-5SA and TAZ-4SA suppressed cardiac reprogramming induced by soft ECM and ROCK inhibitor. **(F)** Quantification of $cTnT^+$ cells; n = 3, independent triplicate experiments.
(G) Quantification of the number of spontaneously beating cells after 4 weeks (n = 4, independent triplicate experiments). Activation of YAP/TAZ suppressed ROCK inhibitor-mediated cardiac reprogramming on 8 kPa hydrogels.
(H, I) Immunocytochemistry of cTnT and DAPI after 4 weeks. YAP-5SA and TAZ-4SA suppressed cardiac reprogramming induced with soft ECM and myosin II inhibitor. **(I)** Quantification of $cTnT^+$ cells; n = 3 independent triplicate experiments.
(J) Quantification of the number of spontaneously beating cells after 4 weeks (n = 4 independent triplicate experiments). Activation of YAP/TAZ suppressed myosin II inhibitor-mediated cardiac reprogramming on 8 kPa hydrogels.
All experiments were performed on 8 kPa hydrogels. All data are presented as the means ± SD. *P < 0.05 versus the relevant control. CTRL, control; inh, inhibitor. Scale bars = 100 µm.

Supplemental Table Legends

Table S1. shRNAs used in this study, related to Methods.

shRNA	Sense strand sequence
Scramble	GCGCGCTTTGTAGGATTCG
shYAP#1	TGAGAACAATGACAACCAATA
shYAP#2	CGGTTGAAACAACAGGAATTA
shTAZ#1	CAGCCGAATCTCGCAATGAAT
shTAZ#2	CCTGCATTTCTGTGGCAGATA

Table S2. TaqMan probes (Applied Biosystems) and qRT-PCR primer sequences for Universal Probe Library (Roche), related to Methods.
(A) TaqMan Gene Expression Assays (Applied Biosystems)

Gene name	Primer/Probe Set
Adrb1	Mm00431701_s1
Col1a1	Mm00801666_g1
Ctgf	Mm01192933_g1
Hcn4	Mm01176086_m1
Itgb2	Mm00434513_m1
Myh6	Mm00440359_m1
Myl2	Mm00440384_m1
Myl7	Mm00491655_m1
Pln	Mm04206542_m1
Serpine1	Mm00435858_m1
Taz (Wwtr1)	Mm01289583_m1
Thy1	Mm00493681_m1

Tnnt2	Mm01290256_m1	
Yap	Mm01143263_m1	

(B) Universal Probe Library System (Roche)

Gene name	Primer	Universal Probe Library
Gapdh	AGCTTGTCATCAACGGGAAG	#9
	TTTGATGTTAGTGGGGTCTCG	

Table S3. Concentration of acrylamide (AAm) and N,N'-methylenebisacrylamide (BIS) and the corresponding Young's modulus in polyacrylamide hydrogels, related to Methods.

AAm (%)	BIS (%)	ACA (mM)	Young's modulus (kPa)	Notation in text
7.5	0.64	70	126.2	126 kPa
7.5	0.32	90	67.2	67 kPa
7.5	0.16	115	30.1	30 kPa
7.5	0.08	150	13.8	14 kPa
7.5	0.04	190	7.5	8 kPa
7.5	0.02	245	4.0	4 kPa
7.5	0.01	306	2.0	2 kPa
7.5	0.005	392	≦1	1 kPa

Supplemental Movie Legend

Movie S1, related to Figure 1B.
Spontaneously beating GHMT-induced iCMs cultured on 8 kPa hydrogels for 4 weeks.

Supplemental Experimental Procedures

Generation of αMHC-GFP transgenic mice and fibroblast isolation
The Keio and Tsukuba University Ethics Committees for Animal Experiments approved all experiments in this study. Transgenic mice overexpressing GFP under the control of an α-MHC promoter were generated as described previously (Ieda et al., 2010). Mouse fibroblasts were isolated as described previously (Muraoka et al., 2014). In brief, mouse embryos isolated from 12.5-day pregnant mice were washed with phosphate-buffered saline (PBS), followed by careful removal of the head and visceral tissues. The remaining parts of the embryos were washed in fresh PBS, minced using a pair of scissors, transferred to a 0.125% trypsin/EDTA solution (25200-072; Gibco; Thermo Fisher Scientific, Waltham, MA), and incubated at 37 °C for 20 min. After trypsinization, an equal amount of fetal bovine serum (FBS) was added and pipetted several times to allow for tissue dissociation. After centrifugation of the tissue/medium mixture at 5000 × *g* for 5 min, the cells were collected and resuspended for culturing in DMEM (044-29765; Wako, Osaka, Japan) supplemented with 10% FBS at 37 °C in 5% CO_2 or stored in liquid nitrogen. Fibroblasts were plated at a density of 17,500–35,000 cells/cm^2 on PS dishes or hydrogels with various elasticities for virus transduction.

Measuring polyacrylamide gel elasticity
Gel rigidity was determined by the penetration method (Yip et al., 2013). In brief, Young's modulus (E) was obtained using the Hertz sphere model $\mathbf{h = bf^{2/3}}$, where $b = [9/(16\ ER^{1/2})]^{2/3}$ and a value of 1/2 for the Poisson ratio was assumed. The indentation profiles were obtained from fully hydrated 2–3 mm gel samples with a stainless-steel sphere (3 mm radius [R]). The force exerted on gels (f) was measured using a custom-designed electronic balance where gels were placed. Indentation of the sphere (h) was controlled using a micrometer. The Hertz model was then applied to fit the first linear section in the plot $f^{2/3}$ against indentation depth to ensure that the estimation was consistent with the linear approximation.

Retrovirus infection, lentivirus infection, Sendai virus vector infection, and cell culture
For constructing retroviral vectors, we used pMXs retroviral vectors for GFP, Gata4, Mef2c, Tbx5, and Hand2, which were described previously(Ieda et al., 2010). pQCX1H Myc-YAP (5SA) was obtained from Addgene (#33093; Watertown, MA) and pBABE FLAG-TAZ (4SA) was a gift from Dr. Guan (University of California San Diego)(Moroishi et al., 2016; Zhao et al., 2007). The vectors were transfected into Plat-E cells using Polyethylenimine "MAX" (24765-2; Polysciences Inc., Warrington, PA) according to the standard protocol. shRNA constructs were designed to target 21-base pair gene-specific regions in mouse YAP and TAZ. These oligonucleotides or scrambled oligonucleotides were cloned into CS-RfA-CG lentiviral vectors (RIKEN BioResource Center [BRC], Tsukaba, Japan) using the Gateway system (12538-120; Invitrogen, Carlsbad, CA).

To generate lentiviruses, we transfected the vectors into 293T cells with the packaging plasmids pMDL g/pRRE (RIKEN BRC) and pCMV-VSV-G-RSV-Rev (RIKEN BRC) using Lipofectamine 2000 (11668-019; Invitrogen). Retrovirus or lentivirus-containing supernatants were collected after 48 hours, filtered through 0.45-mm pore membranes, mixed with polybrene (TR-1003-G; Millipore, Burlington, MA) to a final concentration of 4 µg/mL, and transduced into fibroblasts. We routinely used suitable controls, pMX-GFP and CS-RfA-CG (containing CMV-GFP), to monitor transduction efficiency; we obtained efficiencies of 95%–100% in this study. The medium was replaced with DMEM/M199 (11150-059; Gibco) supplemented with 20% FBS after 24 h of infection, followed by replacement with FFV medium after 2 weeks, as described previously(Yamakawa et al., 2015). Generation and transduction of Sendai viral (SeV) vectors were performed as described previously (Miyamoto et al., 2018). Briefly, to generate F-deficient temperature-sensitive (TS) SeV vectors (SeV/TSΔF) expressing GMT, a polycistronic Gata4-Mef2c-Tbx5 was inserted into the cDNA of SeV/TSΔF vector (Ban et al., 2011; Li et al., 2000). Fibroblasts were transduced with the SeV vectors and cultured in DMEM/10% FBS at 35 °C in 5% CO_2 for 16 h. The medium was replaced with the FFV medium after 24 h of infection, and the cells were subsequently cultured at 37 °C in 5% CO_2. FFV medium contained StemPro-34 SF medium (10639-011; Gibco), GlutaMAX (10 µL/mL; 35050-061; Gibco), ascorbic acid (50 µg/mL; A-4544; Sigma-Aldrich, St. Louis, MO), recombinant human VEGF165 (5 ng/mL; 293-VE-050; R&D Systems, Minneapolis, MN), recombinant human FGF basic 146 aa (10 ng/mL; 233-FB-025; R&D Systems), and recombinant human FGF10 (50 ng/mL; 345-FG-025; R&D Systems). The medium was changed twice a week. Integrin signaling inhibitors (PF-00562271; S2672, 1 µM; Cayman, Ann Arbor, MI, RGDS peptide; 3498, 100 µM; R&D Systems), ROCK inhibitor (Y-27632; 250-00613, 30 µM; Wako) and Myosin II inhibitor (blebbistatin; 13013; 10 µM; Cayman) were added to DMEM/M199/20% FBS medium and FFV medium as indicated.

FACS analysis
Cells were fixed in 4% paraformaldehyde (PFA) for 15 min and stained with anti-cTnT antibody (MS-295-P1; Thermo Fisher Scientific) followed by incubation with secondary antibody conjugated to Alexa Fluor 647 (A21240; Invitrogen)(Ieda et al., 2010). The cells were resuspended in 5% FBS/PBS including saponin (0.5%; 47036-250G-F; Sigma-Aldrich) and analysed using a FACS instrument (CytoFLEX S; Beckman Coulter, Brea, CA).

Immunocytochemistry
Cells were fixed in 4% PFA for 15 min at room temperature, blocked with 5% Normal Goat Serum Blocking Solution (S-1000; Vector Laboratories, Burlingame, CA), and incubated with primary antibodies against α-actinin, cTnT, GFP (598; MBL, Woburn, MA), and YAP/TAZ (#8418; Cell Signaling Technology, Danvers, MA). The cells were then incubated with secondary antibodies conjugated to Alexa Fluor 488 (A11008; Invitrogen) or Alexa Fluor 546 (A11010, A11003; Invitrogen), followed by counterstaining with DAPI (D1306; Invitrogen). The percentage of GFP, α-actinin, and cTnT-immuno-positive cells was counted in 10–15 randomly selected fields per well in at least three independent experiments with 2000–4000 cells counted in total. The measurements and calculations were conducted in a blinded manner. The percentage of nuclear YAP/TAZ was also counted in 10–15 randomly selected fields per well in at least three independent experiments with 2000–4000 cells counted in total. All experiments were performed using an All-in-One fluorescence microscope (BZ-X810; Keyence, Osaka, Japan)(Yamakawa et al., 2015).

Western blot analysis
Lysates were prepared by homogenization of cells in RIPA buffer (08714-04; Nacalai Tesque) and then run on sodium dodecyl sulphate-polyacrylamide gels to separate proteins prior to immunoblot analyses. After transferring to PVDF membranes, immunodetection was performed using antibodies against GATA4 (sc-25310; Santa Cruz, Dallas, TX), MEF2C (ab197070; Abcam, Cambridge, UK), TBX5 (13178-1-AP; Proteintech, Rosemont, IL), YAP/TAZ (#8418; Cell Signaling Technology), phospho-YAP (Ser127; #13008; Cell Signaling Technology), phospho-TAZ (Ser89; #59971; Cell Signaling Technology), LATS1 (#3477; Cell Signaling Technology), phospho-LATS1 (Thr1079; #8654; Cell Signaling Technology), and GAPDH (#2118; Cell Signaling Technology) followed by treatment with horseradish peroxidase-conjugated anti-rabbit IgG secondary antibody (#7074S; Cell Signaling Technology). The antibody-bound proteins were visualised by Chemi-Lumi One L (#07880; Nacalai Tesque) and imaged using a CCD camera imaging system (ImageQuant LAS500; GE Healthcare, Chicago, IL)(Miyamoto et al., 2018). Signal intensity was measured by Image J software (National Institutes of Health [NIH], Bethesda, MD).

EdU labeling assay
To assess cell proliferation, 10 µM EdU was added to the culture medium 2 weeks after transduction and this concentration was maintained throughout the culture for additional 2 weeks. Cells were fixed with 4% PFA for 15 min, permeabilized, and incubated with anti-cTnT antibody. Next, the cells were incubated with secondary

antibodies conjugated to Alexa Fluor 546 (for immunocytochemistry) or 647 (for FACS), and EdU was detected using the Click-iT EdU Alexa Fluor 488 HCS Assay (C10350; Invitrogen) according to the manufacturer's instructions.

Counting of beating cells

For accurate analyses of the cell count, we used the All-in-One fluorescence(Miyamoto et al., 2018; Yamakawa et al., 2015). Cells were maintained at 37 °C and 5% CO_2 using the controlled chamber within the microscope. The number of spontaneously contracting cells in each field were counted for 25 regions with the 20× phase contrast lens; at least three independent experiments were performed. Individual beating cells were identified based on differences in beating frequency, cell-membrane boundary, and nuclei identified by phase-contrast microscopy. Cardiac reprogramming efficiency was determined as the number of beating cells per total nuclei counts at the time of the assay. Measurements and calculations were conducted in a blinded manner.

Ca^{2+} imaging

Ca^{2+} imaging was performed following standard protocols. Briefly, cells were labeled with Fluo-4 AM solution (F14201; Thermo Scientific, Waltham, MA) for 30 min at room temperature, washed, and incubated for additional 30 min, to allow de-esterification of the dye. Fluo-4-labeled cells were analyzed using an All-in-One fluorescence microscope at 37 °C. Ca^{2+} oscillation$^+$ cells were manually counted in 15 randomly selected fields per well, in three independent experiments. The measurements and quantification of the results were conducted in a blinded manner.

Quantitative RT-PCR

Total RNA was isolated from cells using the ReliaPrep RNA Cell Miniprep System (Z6012; Promega, Madison, WI) and then qRT-PCR was performed on a ViiA7 (Applied Biosystems, Foster City, CA) with TaqMan (Applied Biosystems) or Roche (Basel, Switzerland) probes. Primer and probe details are provided in Table S2. mRNA levels were normalised relative to *Gapdh*.

Gene microarray analyses

Microarray analyses were performed in duplicate, using independent biological samples. RNA was extracted from cells using the ReliaPrep RNA Cell Miniprep System. RNA quality was determined by the RNA Integrity Number (RIN) value using an RNA6000 assay (Agilent Technologies, Santa Clara, CA). Only specimens with RIN > 7.0 were used in this study. Gene expression levels were determined via microarrays (Clariom S Array, Mouse; Affymetrix, Santa Clara, CA) according to manufacturer's instructions. Prior to analysis, all data were normalised using a Single Space Transformation and Robust Multichip Analysis (SST-RMA) algorithm with Affymetrix Expression Console software version 1.4. Heatmaps and Venn diagrams were generated using GeneSpring GX 14.8 software (Agilent Technologies). GO analyses were performed using Database for Annotation, Visualization, and Integrated Discovery (DAVID; http://david.abcc.ncifcrf.gov/). Gene set enrichment analyses (GSEA) were performed using GSEA software (Broad Institute, Cambridge, MA).

Motion analyses of iCMs

To evaluate the functional parameters of iCMs, we used the SI8000 Cell Motion Imaging System (SONY, Tokyo, Japan), a high-resolution motion capture tracking system and a high speed video microscopy to detect and record cell motion. Spontaneous beating activity of iCMs was recorded and the motion of each detection point was converted into a motion vector. Quantitative analyses of the contraction velocity (CV) and the relaxation velocity (RV) of beating iCMs were also performed. The maximum CV and RV correspond to the contractile and diastolic function of iCMs, respectively (Ito et al., 2019; Kitani et al., 2019). Beating iCMs were recorded in 10 randomly selected fields per well. All movies were recorded with the resolutions of 2048 × 2048 pixels at 150 fps. Maximum CV and RV were assessed from the averaged contraction-relaxation waveforms during a 5 s recording.

Statistical analysis

Statistical parameters including the number of samples (n), descriptive statistics (mean and standard deviation), and significance are reported in the Figures and Figure legends. In general, at least n = 3 were used for each time point and experiment. Differences between groups were examined for statistical significance using the Student's *t*-test or one-way analysis of variance (ANOVA) followed by Dunnett's post-hoc test. Differences with P values < 0.05 were regarded as significant.

Supplemental References

Ban, H., Nishishita, N., Fusaki, N., Tabata, T., Saeki, K., Shikamura, M., Takada, N., Inoue, M., Hasegawa, M., Kawamata, S., et al. (2011). Efficient generation of transgene-free human induced pluripotent stem cells (iPSCs)

by temperature-sensitive Sendai virus vectors. Proceedings of the National Academy of Sciences of the United States of America 108, 14234-14239.
Ieda, M., Fu, J.D., Delgado-Olguin, P., Vedantham, V., Hayashi, Y., Bruneau, B.G., and Srivastava, D. (2010). Direct reprogramming of fibroblasts into functional cardiomyocytes by defined factors. Cell 142, 375-386.
Ito, M., Hara, H., Takeda, N., Naito, A.T., Nomura, S., Kondo, M., Hata, Y., Uchiyama, M., Morita, H., and Komuro, I. (2019). Characterization of a small molecule that promotes cell cycle activation of human induced pluripotent stem cell-derived cardiomyocytes. Journal of molecular and cellular cardiology 128, 90-95.
Kitani, T., Ong, S.G., Lam, C.K., Rhee, J.W., Zhang, J.Z., Oikonomopoulos, A., Ma, N., Tian, L., Lee, J., Telli, M.L., et al. (2019). Human-Induced Pluripotent Stem Cell Model of Trastuzumab-Induced Cardiac Dysfunction in Patients With Breast Cancer. Circulation 139, 2451-2465.
Li, H.O., Zhu, Y.F., Asakawa, M., Kuma, H., Hirata, T., Ueda, Y., Lee, Y.S., Fukumura, M., Iida, A., Kato, A., et al. (2000). A cytoplasmic RNA vector derived from nontransmissible Sendai virus with efficient gene transfer and expression. Journal of virology 74, 6564-6569.
Miyamoto, K., Akiyama, M., Tamura, F., Isomi, M., Yamakawa, H., Sadahiro, T., Muraoka, N., Kojima, H., Haginiwa, S., Kurotsu, S., et al. (2018). Direct In Vivo Reprogramming with Sendai Virus Vectors Improves Cardiac Function after Myocardial Infarction. Cell Stem Cell 22, 91-103 e105.
Moroishi, T., Hayashi, T., Pan, W.W., Fujita, Y., Holt, M.V., Qin, J., Carson, D.A., and Guan, K.L. (2016). The Hippo Pathway Kinases LATS1/2 Suppress Cancer Immunity. Cell 167, 1525-1539 e1517.
Yamakawa, H., Muraoka, N., Miyamoto, K., Sadahiro, T., Isomi, M., Haginiwa, S., Kojima, H., Umei, T., Akiyama, M., Kuishi, Y., et al. (2015). Fibroblast Growth Factors and Vascular Endothelial Growth Factor Promote Cardiac Reprogramming under Defined Conditions. Stem Cell Reports 5, 1128-1142.
Yip, A.K., Iwasaki, K., Ursekar, C., Machiyama, H., Saxena, M., Chen, H., Harada, I., Chiam, K.H., and Sawada, Y. (2013). Cellular response to substrate rigidity is governed by either stress or strain. Biophys J 104, 19-29.
Zhao, B., Wei, X., Li, W., Udan, R.S., Yang, Q., Kim, J., Xie, J., Ikenoue, T., Yu, J., Li, L., et al. (2007). Inactivation of YAP oncoprotein by the Hippo pathway is involved in cell contact inhibition and tissue growth control. Genes Dev 21, 2747-2761.
